# Computational study of associations between histone modification and protein-DNA binding in yeast genome by integrating diverse information

**DOI:** 10.1186/1471-2164-12-172

**Published:** 2011-04-01

**Authors:** Junbai Wang

**Affiliations:** 1 Department of Pathology, The Norwegian Radium Hospital, Oslo University Hospital, Montebello 0310 Oslo, Norway

## Abstract

**Background:**

In parallel with the quick development of high-throughput technologies, *in vivo (vitro) *experiments for genome-wide identification of protein-DNA interactions have been developed. Nevertheless, a few questions remain in the field, such as how to distinguish true protein-DNA binding (functional binding) from non-specific protein-DNA binding (non-functional binding). Previous researches tackled the problem by integrated analysis of multiple available sources. However, few systematic studies have been carried out to examine the possible relationships between histone modification and protein-DNA binding. Here this issue was investigated by using publicly available histone modification data in yeast.

**Results:**

Two separate histone modification datasets were studied, at both the open reading frame (ORF) and the promoter region of binding targets for 37 yeast transcription factors. Both results revealed a distinct histone modification pattern between the functional protein-DNA binding sites and non-functional ones for almost half of all TFs tested. Such difference is much stronger at the ORF than at the promoter region. In addition, a protein-histone modification interaction pathway can only be inferred from the functional protein binding targets.

**Conclusions:**

Overall, the results suggest that histone modification information can be used to distinguish the functional protein-DNA binding from the non-functional, and that the regulation of various proteins is controlled by the modification of different histone lysines such as the protein-specific histone modification levels.

## Background

The binding of transcription factors (TF) to DNA sequences is an essential step in genome regulation. In parallel with the quick development of high-throughput methods for measuring genome-wide protein-DNA interaction (e.g., ChIP-chip [1], ChIP-Seq [2], DamID [3], and protein binding microarray [4]). Many state-of-art computer programs (e.g., MEME [5], MatrixReduce [6], and MDScan [7]) have been developed to identify TF binding motifs. Nevertheless, several questions remain in the field, such as how to distinguish true TF-DNA binding (functional TF binding sites) from non-specific TF-DNA binding (non-functional ones). Here the functional TF binding site is defined as the promoter region of a gene that, bound by a TF, is a true regulatory target (e.g., a strong correlation between the inferred TF activity and mRNA expression of a gene that is bound by the TF [8,9]); the non-functional TF binding site refers to a non-specific TF-DNA binding such as a TF that is bound to the promoter region of a gene but does not regulate the gene expression. Finding the true regulatory targets of a TF based on the present technology is a challenge [10], which has inspired many researchers over the past several years to seek help from computational solutions such as integrative modeling of mRNA expression data and ChIP-chip data [8], biophysical modeling of orthologous promoter sequences [11], predicting of functionality of protein-DNA interactions [9], and distinguishing direct versus indirect TF-DNA interactions [12] by integrating diverse information.

Although some of the previous studies considered the effect of nucleosomes on TF-DNA interactions (e.g., nucleosome occupancy affects transcription by decreasing the accessibility of DNA to protein binding [13]), most of them ignored an important aspect that is also closely associated with functional TF binding, that is, changes in chromatin structure are affected by histone modifications such as methylation and acetylation [14,15]. In a few recent papers [9,16], the effect of histone modifications on protein-DNA interactions was emphasized. Especially, several excellent bioinformatics studies revealed importance of considering histone modification information, in computational algorithms, for identifying new regulatory elements [17] and predicting promoters and enhancers in the human and mouse genomes [18,19]. However, no conclusive remarks were made to address the associations between histone modification and functional TF binding. This may be due to the ongoing debate on models of the functions of histone modification [2]. Currently, three major models have been proposed to explain the role of histone modification in genome regulation: 1) charge neutralization [20], by which histone modification can relax chromatin structure because of neutralizing positive charges on DNA; 2) histone code [21], by which combinatory histone modifications can regulate downstream gene functions; and 3) signaling pathway [22,23], by which multiple histone modifications can provide bi-stability and robustness through feedback loops. Motivated by this unsolved question, a systematic study of associations between TF-DNA binding and histone modification in yeast was carried out by integrative analysis of diverse datasets [8,9,24-27].

## Methods

### Pre-processing of datasets

ChIP-chip experimental data in rich medium conditions of 203 yeast TFs was obtained from the work of Harbison et al. [24]. Yeast nucleosome occupancy in normal condition was taken from Lee et al. [25]. The histone acetylation dataset was from Kurdistani et al. [27]; the dataset contained acetylation levels on 11 histone lysines in both yeast promoter and the open reading frame (ORF) (H2aK7, H2bK11, and 16; H3K9, 14, 18, 23, and 27; H4K8, 12, and 16). Because the measured histone modifications in any given promoter are affected by the rate of that region being occupied by nucleosome [26], the 11 acetylation levels were normalized by the nucleosome occupancy (H3 and H4) measured by Lee et al. [25]. More specifically, the average of H3 and H4 histone levels was computed within each probe then the histone acetylation level of that probe was divided by the corresponding mean nucleosome occupancy. Additionally, histone modification data from Pokholok et al. [26] was used, which included acetylation levels on three histone lysines (H4; H3K9 and 14), methylation levels on five histone lysines (H3K4me1, 4me2, 4me3; H3K36me3; H3K79me3), nucleosome occupancy (H3 and H4), and histone acetyltransferase (ESA1 and GCN5) occupancy data under normal condition. Here the histone modification signals were also normalized by the local nucleosome occupancy as described in the previous dataset. Since array difference in genome-wide coverage (e.g. data from Kurdistani et al. contains only ~1580 promoters and ~2384 ORFs; but a high-resolution microarray data from Pokholok et al. includes ~5522 ORFs and ~5504 promoters), the above-mentioned two histone modifications datasets were separately analyzed. All datasets were transformed to Z-scores before further data analysis was performed.

### Gene assignment, putative functional binding target and data analysis

Based on the original gene annotation tables from [25-27], an in-house Perl script file was used to map nucleosome occupancy and histone modification levels to gene and the corresponding promoter region, in which if multiple probes are assigned to the same gene or promoter region then we use their mean value. Information on computationally inferred functional TF binding sites and non-functional ones for 37 yeast TFs at normal condition was taken from publication by Gao et al. [8]. Here TFs with less than five probes overlapped between the binding data and the histone modification data were excluded. To examine possible correlations between histone modifications and transcription factor binding, a two-tailed t-test was used to quantify the difference in mean between the TF binds and the histone modification [28] for both the functional binding probes (bind and couple) and non-functional ones (bind but not couple), respectively. In general, the t-test was used to score the difference between average TF binding affinity (histone modification level) of predefined groups of probes (e.g. functional binding probes), and that of all other probes on the array. Subsequently, the t-values were clustered [29] and visualized [30] in a color-coded heat map to uncover TF binds (histone modification) enriched in the probed regions forming a given group. The same procedure was successfully applied in a number of earlier studies [28,31]. Finally, to evaluate the robustness of the t-test, the rank-sum test was applied on the same datasets, and then the log10 transformed p-values were displayed in the heat map.

### Protein-histone modification interaction networks

In order to investigate possible correlation between the histone modification at ORF and the TF binding to the corresponding promoter, a computational strategy was used to build a protein-histone modification interaction network: 1) for the binding targets of each of 32 TFs from [8], enrichment of proteins (total 203 yeast TFs [24]) binding to the promoter was tested, such as by performing a two-tailed t-test for selected functional (or non-functional) binding sites versus the rest of the binding sites in the yeast genome [28]; 2) then, for the binding targets of the above-mentioned 32 TFs, the same t-tests were used to evaluate the histone modification changes (total 8 histone modifications [26]) at the corresponding ORF; 3) subsequently, the t-values from the previous tests were combined together, more specifically, the histone modifications at the ORF of functional (or non-functional) binding sites were combined with the enrichment of TF binding at the corresponding promoter; 4) in each of above two newly complied datasets, one for functional binding sites and the other for non-functional ones, proteins (203 TFs and 8 histone modifications) were grouped into 18 clusters by using a published computational approach [32] that combines the stress function, neuron gas algorithm and K-nearest neighbour method, where the number of protein clusters was automatically estimated by the stress function; 5) Finally, Gaussian Graphical Models [33,34] were applied on the centers of 18 clusters for inferring the protein-histone modification interaction network. In predicted network, the nodes represent 18 protein clusters and the edges indicate associations between a pair of nodes, where the strength of interactions is stated by the partial correlation coefficient. For every node that is connected to the network, its representative proteins are labeled.

### Bayesian Neural Networks

Binary Bayesian Neural Classification Networks is a supervised neural network, the output of which is a function *y *of the input *x *and of the parameters *w*; the architecture of net is denoted by *A*. The output function *y*(*x; w, A*) is bound between 0 and 1 such as a probability *P*(*t *= 1 | *x,w,A*), where *t *are the targets in a dataset which are binary classification labels (0, 1). Here we trained the network with one hidden layer to perform classification tasks. The non-linear 'sigmoid' function at the hidden layer gives the neural networks greater computational flexibility than a standard linear regression model [35]. The objective function of the networks is(1)

where(2)(3)

*E*_*w *_is regularization, and *i *and *m *are the number of parameters and the number of input data, respectively. Based on Bayes' theorem, a posterior distribution of the model parameters *w *is(4)

where *H *represents the model hypothesis space such as network structure and regularization, *M *is a probability framework of the objective function described in equation (1), and *Z*_*M *_is a normalization factor. By using a Gaussian approximation to the posterior probability, we minimize objective function (1) and determine the re-estimation formulas for hyperparameter α according to the weight assumptions *E*_*w*_. A detailed description of computational implementation of Bayesian Neural Networks by using the Gaussian approximation for the posterior distribution is available in previous publications [35-37].

### Using Bayesian Neural Networks to find functional protein-DNA binding

As already discussed in the previous section, Bayesian Neural Networks is a supervised non-linear model, which has several advantages [35] when applied to classification tasks: 1) the computational algorithm is robust [38], 2) it can learn from the data without any pre-assumption, 3) its non-linear feature can be applied to model any real-world complex relationships. Thus, Bayesian Neural Networks was used to classify functional and non-functional binding sites [8] based on histone modification levels at the ORFs. First, we trained a classifier on the training data for each TF via Bayesian Neural Networks [38] (one hidden layer with two hidden neurons), then the trained classifier was applied on independent test data for recording the percentage of correct classifications and total number of correct classifications. To avoid the bias that may be introduced by the selection of training and test data, we randomly divided the half-available binding sites into the training and the test set, respectively. The random splitting was repeated 10 times for each TF, and the reported classification accuracy is the mean of percentage of correct classifications (MPCC) of 10 randomly selected test datasets. A corresponding 10-fold cross validation was also performed.

## Results

### Histone acetylation (Kurdistani et al.)

**Figure 1:**
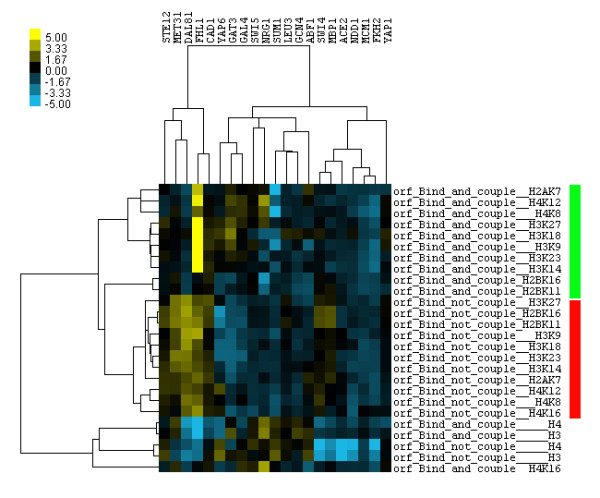
**Hierarchical clustering of t-values obtained from the t-test of acetylation levels on 11 histone lysines (Kurdistani SK et al.) in the coding region (ORF)**. For 21 yeast TFs in both functional binding sites (bind and couple) and non-functional binding sites (bind not couple).

Figure 1 and Additional file 1 Figure S1 show the hierarchical clustering of t-values (histone modification activities) at ORF (total 21 TFs) and promoter (total 12 TFs), respectively. From both figures, a distinct histone acetylation pattern separating the functional binding sites and the non-functional ones was observed for several TFs (e.g. MET31, DAL81, FHL1, CAD1 and NRG1 etc.). In particular, the difference is clearer at the ORF than that at the promoter region. Based on the same data, we also computed log10 transformed p-values of both t-test and rank-sum test, then displayed them in heat maps. The heat maps of the two different statistical tests matched each other well and also confirmed the above finding (please refer to Additional file 2).

Among the non-functional binding sites in Figure 1, there is an almost constant acetylation level across all histone lysines, which may be either high or low; for the functional binding sites, however, the equilibrium of the histone acetylation levels on the different lysines is broken, which results in a TF-specific perturbation of acetylation levels. For example, the functional binding sites of FHL1 (a transcriptional activator) show very high acetylation levels on H3K9, 14, 18, 23 and 27, but relatively no acetylation changes on H2K11 and 16; on the other hand, the functional binding sites of NRG1 (a transcriptional repressor) display high acetylation levels on H4K8 and 12 but low acetylation levels on H3K9, 14, 23, and 18, and H2K11 and 16, respectively. From the literature [39], we know that the effect of histone acetylation is dependent on the specific histone lysines that may initiate different downstream functions, such as the binding of additional histone acetyltransferases (HATs), modification of the chromatin structure, and recruitment of a particular transcription factor or nucleosome remodeling complex.

Nevertheless, Figure 1 also shows that the discriminative power of histone modifications is much less clear for other TFs (e.g. NDD1, MCM1, FKH2, ACE2, YAP1, SUM1 etc.). Of the above-mentioned six TFs, the first four are related to the yeast cell cycle and the other two are usually not functional under growth conditions [37]. It suggests that for certain TFs, we need to consider more diverse histone modification information, such as histone decetylation and methylation levels, in order to distinguish the functional binding sites from the non-functional ones. Taken together, the results indicate the TF-specific histone acetylation at yeast ORF might be used as a biomarker of functional protein binding.

### Histone modification (Pokholok et al.)

**Figure 2:**
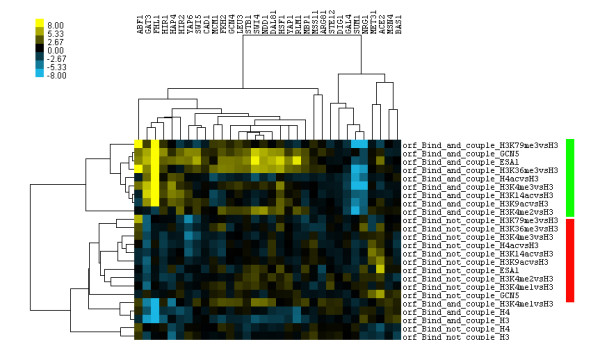
**Hierarchical clustering of t-values obtained from the t-test of 8 histone modifications (Pokholok et al.) in the gene coding region (ORF)**. For 32 yeast TFs in both functional binding sites (bind and couple) and non-functional binding sites (bind not couple).

In the above analysis, only ~1580 promoters and ~2384 ORFs were studied. Next the t-test was performed on a high-resolution microarray data [26] that tiled the entire yeast genome (~260-bp per probe), which contains histone modification data of ~5522 ORFs and ~5504 promoters. After removing TFs with less than 20 binding targets, 32 of the 37 TFs from Gao et al. [8] were used in the study. For those selected TFs, a search of motif similarity matches, by using STAMP tool [40], shows proteins do not bind to the same sites (Additional file 1, Figure S2 and Additional file 3). Figure 2 and Additional file 1 Figure S3 present the clustering of the t-values (histone modification activities) for ORF and promoter, respectively. Both figures indicate a clear separation of histone modification levels, as well as of HATs occupancy (e.g., ESA1 and GCN5) between the functional binding sites and the non-functional ones for almost half of all TFs tested (e.g., ABF1, GAT3, FHL1, HAP4, HIR2, YAP6, SWI4, NDD1, DAL81, SUM1, NRG1 and MET31 etc.). Such effect may be more easily seen at the ORF than that at the promoter. Based on the same data, a similar test (functional binding sites versus non-functional ones) was also performed with both t-test and rank-sum test. The log10 transformed p-values of the above two tests are displayed in heap maps, which show that histone modification levels do differ between the functional binding sites and the non-functional ones (please refer to Additional file 2).

In Figure 2, acetylation levels on histone lysines of both FHL1 and NRG1 bear a similar trend as those observed in Figure 1. For the above-mentioned two TFs, a similar variation at three methylation levels on the histone lysines (H3K4me3, 36me3, and 79me3) was also observed, and the three methylation sites on the histones are involved in the activation of transcription [21] as usually the acetylation on the histone lysines is. Interestingly, for both cell cycle related TFs (e.g. MCM1, FKH2, NDD1 and ACE2) and TFs that are not functional under growth conditions (e.g. YAP1 and SUM1), the discriminative power of histone modifications in Figure 2 is much stronger than that in Figure 1. This may be caused by histone acetyltransferase and methylation levels (e.g. H3K79me3, H3K36me3, H3K4me3 and H3K4me2) in Figure 2. Thus, the new results support our previous hypothesis from Figure 1: the functional regulation of different TFs is controlled by the histone modifications on different lysines; the difference in histone modifications is much stronger at the ORF than at the promoter; in particular, the more diverse histone modification information, the stronger discriminative power it has.

### A protein-histone modification interaction network (Pokholok et al.)

**Figure 3:**
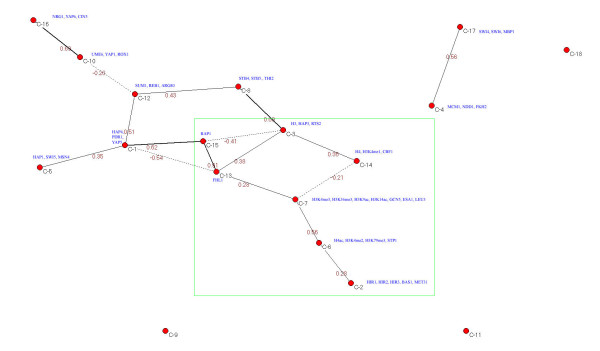
**A protein-histone modification interaction network was predicted by applying Gaussian graphical models (significance level p < 0.003) on the centers of 18 protein clusters**. Each cluster represents a group of proteins that share similar t-values (results of t-tests of functional binging sites versus the rest of binding sites in the genome, for binding of 203 yeast TFs in promoter and variation of 8 histone modifications in ORF - Pokholok et al.) across 32 yeast TFs: blue colored texts are representative proteins of each cluster, red colored number on each edge is partial correlation coefficient between two vertices.

Having shown a difference in histone modification patterns between functional binding sites and non-functional ones at yeast ORF (Figures 1 and 2, respectively) for almost half of all TFs tested, we investigated whether there is a correlation between the histone modifications at the ORF and the TF bindings to the corresponding promoter. This was achieved by a computational strategy that is detailed in the method section. In brief, first protein activity profiles (t-values for 203 TFs and 8 histone modifications) were computed, at both functional and non-functional binding sites of 32 selected TFs [8]; the protein activities were then grouped into 18 clusters (a protein cluster represents a group of proteins with similar activity profiles across 32 TFs [28]; detailed information on the 18 clusters is available in Additional files 4 and 5) by a published algorithm [32]; subsequently, Gaussian Graphical Models [33,34] were applied on the centers of 18 clusters for inferring the protein-histone modification interaction network. Figure 3 and Additional file 1 Figure S4 show the inferred protein-histone modification interaction networks (i.e. significance level p < 0.003) for the functional TF binding sites and the non-functional ones, respectively.

In Figure 3, the functional TF binding, several interesting correlations were found between protein binding to the promoter and histone modification at the corresponding ORF: for example, 1) at the center of the network, two clusters (clusters 13 and 15 with one protein in each, FHL1 and RAP1, respectively) are strongly connected to each other (partial correlation coefficient equals 0.91), while being also associated with three other clusters (clusters 1, 3, and 7) that contain histone H3 and histone acetyltransferase; 2) cluster 15 (RAP1) is negatively correlated (partial correlation coefficient equals -0.41) with cluster 3 (histone H3), but cluster 13 (FHL1) is positively associated with both cluster 3 and cluster 14 (histone H4 and H3K4me1). Additionally, much histone crosstalk was observed: for instance, 1) cluster 14 (H4 and H3K4me1) is negatively correlated with cluster 7 (histone acetyltransferase - ESA1 and GCN5; H3K4me3, H3K46me3; H3K9ac, and H3K14ac), but cluster 13 (FHL1) is positively associated with the same cluster; 2) cluster 7 is also correlated with cluster 6 where we found both histone methylation and histone acetylation (e.g., H3K4me2, H3K79me3, H4ac); 3) cluster 6 is connected with cluster 2 where three of five proteins are a chromatin remodeling complex (e.g., hir1, hir2, hir3) that contributes to both nucleosome formation and regulation of histone gene transcription. In summary, the inferred network reveals a number of interesting findings, such as evidence for histone crosstalk, data suggesting that different proteins are affected by different histone modifications, and data supporting that histone modifications are negatively correlated with nucleosome density, while being positively associated with both the chromatin remodeling complex and the binding of FHL1 and RAP1 to the promoter.

In Additional file 1 Figure S4, the non-functional TF binding, all histone modifications plus the nucleosome (H3 and H4) and HATs (ESA1 and GCN5) occupancies are grouped in the same cluster (cluster 7). In other words, there is no difference in histone modification changes across the 32 yeast TFs when TF binds to DNA but does not function. Particularly, many interesting protein-histone modification interactions in Figure 3 are not present at here: for example, cluster 7 neither directly interacts with the chromatin remodeling complex nor is it associated with the binding of RAP1 and FHL1 to the promoter, although the two proteins are still highly connected to each other (clusters 8 and 15). This suggests that the majority of protein-histone modification interactions will disappear if the protein binds to the promoter region of a gene but does not regulate the gene expression.

### Classification of functional and non-functional binding sites by using measured histone modifications at the ORFs

The above data has so far shown that histone modification at ORFs may be used to predict the functional binding sites at promoters (Figures 1 and 2), and that the TF-specific modification of histone lysines disappears at the non-functional binding sites (Figure 3 and Additional file 1, Figure S4). Thus, it was deemed interesting to investigate how reliable the histone modifications at ORFs are in distinguishing functional binding sites from non-functional ones at the promoters. The hypothesis was tested on two datasets: one containing only 11 acetylation levels from Kurdistani et al (21 yeast TFs in Figure 1) with nucleosome occupancy from Lee et al. at ORFs, the other including more diverse histone modifications from Pokholok et al. (32 yeast TFs in Figure 2). Based on the above-mentioned two datasets, Bayesian Neural Networks was used to perform the classification and the MPCC on the test data sets (the half-available binding sites) was reported, Table 1 and Additional file 1 Table S1a. For results of 10-fold cross validations, please refer to Additional file 1, Tables S1b, S2 and Figures S5b, S6b.

**Table 1:** Mean percentage of correct classifications and mean total number of correct classifications for 32 yeast TFs.

Rank	TF Name	Mean percentage of correct classifications in 10 test datasets	Mean total number of correct classifications in 10 test datasets
1	*NRG1*	90	29
2	**FHL1**	87	70
3	**GAT3**	83	9
4	*RLM1*	82	14
5	**HIR1**	80	9
6	**GAL4**	77	7
7	*MSS11*	75	7
8	*SUM1*	73	19
9	**BAS1**	72	13
10	*GCN4*	72	25
11	*DAL81*	71	12
12	**NDD1**	71	29
13	*YAP1*	71	15
14	**MSN4**	70	6
15	**HAP4**	69	17
16	**HIR2**	69	5
17	*HSF1*	69	16
18	**SWI4**	68	37
19	**SWI5**	68	27
20	*YAP6*	65	24
21	**MBP1**	64	31
22	**MET31**	64	9
23	*ARG81*	63	5
24	*CAD1*	63	11
25	*ABF1*	62	81
26	**ACE2**	60	17
27	**FKH2**	60	30
28	**STE12**	60	13
29	*LEU3*	56	5
30	**STB1**	56	6
31	**MCM1**	55	21
32	**DIG1**	51	7

The calculations are completed by applying Bayesian Neural Networks on 10 randomly selected test datasets (the half-available binding sites), which include diverse histone modifications (e.g. 3 histone acetylation, 5 histone methylation levels, nucleosome occupancy, and 2 histone acetyltransferase; Pokholok et al.); TF name with bold text represents yeast cell cycle related TFs [54], and TF rank order is based on mean percentage of correct classification in 10 test datasets.

For the first dataset, Additional file 1 Table S1a shows MPCC of 10 randomly selected test datasets, with 5 TFs (~24%) showing a good prediction rate on the test set (MPCC > = 70%) but with the other 13 TFs (~62%) classifying poorly (MPCC <60%). Among the poorly classified TFs, ~69% (9 TFs) are associated with yeast cell cycle. For the second dataset (Table 1), a clear improvement of the classification accuracy is observed: for example, 14 of the total 32 TFs (~44%) had MPCC > = 70% and the trained classifier only tested poorly on 4 TFs (~13%; MPCC <60%). Here ~67% of the TFs with lower classification accuracies (18 TFs with MPCC <70%) are TFs related to the yeast cell cycle. In brief, for the first training data that contains only histone acetylation information, good classification accuracy was achieved for around one third of all TFs tested (e.g. Table S1a); however, for the second dataset that includes both histone methylation and histone acetlylation features, almost half of all TFs tested were well classified by histone modifications (e.g. Table 1). Additional file 1 Figures S5a and S6a show the mean confusion matrix and the mean classification performance (prediction compared with true target), respectively, of 10 randomly selected test datasets [26,27].

**Figure 4:**
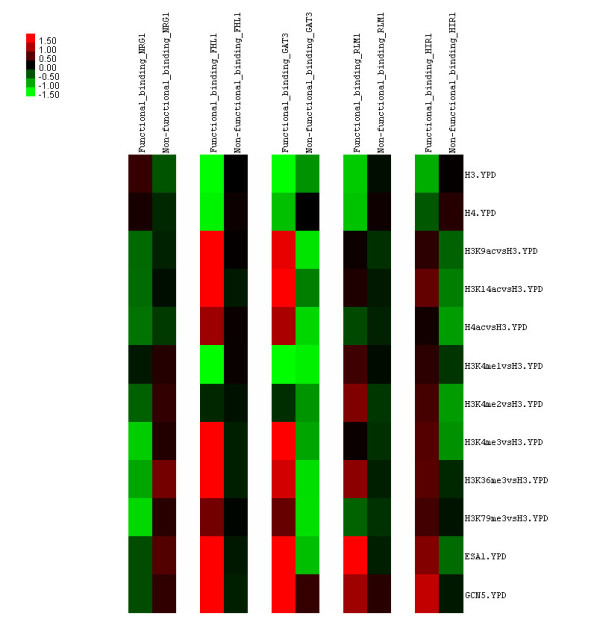
**Mean histone modification levels at coding region (ORF) of either functional or non-functional binding targets (Pokholok et al.)**. For top 5 ranked TFs (NRG1, FHL1, GAT3, RLM1 and HIR1; mean percentage of correct classifications ≥ 80) from Table 1: the color scale represents the mean of normalized log2 transformed ChIP-chip ratios (Z-scores) in functional and non-functional TF binding sites, respectively.

Through a closer look (Figure 4) at the mean histone modification levels at the ORFs of the top 5 ranked TFs (Table 1), two similar regulation mechanisms as previously been seen in Figure 2 were identified. First, NRG1, a transcriptional repressor, shows higher nucleosome density at the ORFs of functional binding targets than at those of non-functional ones, although it has much lower histone modification levels at the ORFs of functional binding sites than at those of non-functional ones; in contrast, the other four TFs (FHL1, GAT3, RLM1, and HIR1, most of them transcriptional activators) show opposite histone modification activities. Second, H4 acetylation levels and H3K79me3 are only depleted in NRG1 and RLM1, while H3K4me1 and H3K4me2 are only enriched in RLM1 and HIR1. Overall, this suggests that there are TF-specific histone modifications at the ORFs of functional binding sites, and that the regulation of different TFs is controlled by the histone modifications on different lysines.

## Discussion and Conclusions

Two histone modification datasets [26,27] were investigated here. Both results confirm there is a distinct pattern of histone modifications between functional TF binding sites and non-functional ones for almost half of all TFs tested (Figures 1 and 2, respectively). For example, 1) for the functional TF binding sites, different TFs modify acetylation (methylation) levels on the different histone lysines; 2) for the non-functional TF binding sites, the acetylation (methylation) levels on different histone lysines are almost constant; 3) the difference in histone modifications between the functional TF binding sites and the non-functional ones is stronger at the ORF region than that at the promoters, which is also becoming clear when we directly compare the mean histone modification changes between the two groups (Additional file 1, Figure S7); and 4) a protein-histone modification interaction network can only be inferred from the functional protein binding targets. In summary, both the histone crosstalk and protein-histone modification interactions may play important roles in functional TF binding since many of them disappear under non-functional conditions.

In particular, the discriminative power of histone modifications is much greater with histone modifications at ORFs than at the promoter. The finding is backed by several lines of evidence in the literature. First, in yeast, the methylation levels on histone lysines are either positively or negatively correlated with transcription rates, and the main peaks of enrichment for methylation are often within the ORFs (e.g., H3K4me1, 4me2, 4me3; H3K36me3 and H3K79me3) [23,41]. Second, although acetylation at many sites correlates with transcription rate, some of them (e.g., H4K16ac, H4K8ac, H2BK11ac and H2BK16ac) at yeast intergenic regions do not correlate well with transcription [41]. Third, in different human cell types, histone modification levels and gene expression are very well correlated, and the main peaks of enrichment for those important modifications are within the ORFs (e.g., H3K4me3, H3K79me1, H4K20me1, and H3K27ac) [42]. Finally, in both the yeast and fruit fly genomes, experimental observations have shown that the enrichment of H3K36me3 levels at the ORFs can be used to distinguish different chromatin types [13,43]. Thus, the high levels of ORF enrichment for histone modification, especially the methylation levels, could potentially reflect the activity of protein-DNA binding in the promoter region [44]. In general, all the above-mentioned molecular mechanisms support the hypothesis that ORF histone modification data are better associated with TF binding at the promoter than the promoter histone modification data, further investigation is still needed to determine and verify the underlying mechanism.

In addition, Bayesian Neural Networks was used to train a classifier from the training histone data, and then the trained classifier was applied to an independent set of histone data in order to predict the functional TF binding sites. The results are encouraging (Table 1) because almost half of the tested TFs could reach a prediction accuracy of ~70%, although only eight histone modifications were considered in the training set. In Table 1, especially, among the top 5 ranked TFs, we observed TF-specific histone modification at the ORFs of functional binding sites (Figure 4), which suggests that the functional regulation of different TFs is controlled by the histone modifications on different lysines (Figure 2). However, most of the currently examined histone modifications are associated with transcriptional activation [45], and there is no information about the histone modifications of transcriptional silencing/repression (e.g., histone decetylation [41] and methylation of H3K27me and H4K20me [23,46]) in the training data. Therefore, the lack of information on certain histone modifications may cause the poor prediction rate for some TFs. For instance, in Table 1, ~67% of TFs with MPCC <70% are TFs related to yeast cell cycle; the cell cycle TFs are often associated with Rpd3 target genes, and the Rpd3 protein belongs to yeast histone deacetylases (HDACs) that may play an important role in yeast cell cycle regulation [47]; after excluding the cell cycle TFs from Figure 1 and 2, clustering analysis was performed again but the clustering patterns were not dramatically changed, Additional file 1 Figures S8 and S9, respectively. Thus, results indicate that if the training data include more post-translational modifications of the histones (e.g., the above mentioned HDACS, H3K27me, and H4K20me, as well as phosphorylation and ubiquitylation [45]), then the trained classifier will achieve a better prediction accuracy in the test data.

Below is a brief description of the possible protein-histone modification interaction network from Figure 3. First, for correlations between protein binding to the promoter and histone modification at the corresponding ORF, cluster 15 (RAP1) has a strong negative association with cluster 3 (histone H3, etc.), but cluster 13 (FHL1) has a strong positive interaction with the same cluster, and cluster 14 (histone H4, H3Kme1, etc.) and cluster 3 are positively correlated to each other. The above mentioned interactions are consistent with the literature: for example, RAP1 (a general transcription factor) opens chromatin [48] to facilitate binding by other TFs such as GCN4, and then the bound TF recruits HATs (e.g. GCN5 and ESA1), resulting in histone acetylation [41]; FHL1 has been thought to interact with the histone acetylase ESA1 and to activate transcription of proteins [49]; by searching the BioGRID database [50], direct protein-protein interaction between RAP1 and FHL1 was found, as well as interactions between RAP1 and nine other proteins associated with HATs (e.g., SAS4, SAS5, RTT109; for detailed information, please refer to Additional file 6), although FHL1 only interacted with one HAT (EAF6). Additionally, both FHL1 and RAP1 are known to actively participate in modifying chromatin structure [48,51] and regulating acetylation/methylation levels on histone lysines [27,49,52]. Thus, current literature supports the view that RAP1 and FHL1 complement each other to control the chromatin-open and HAT recruitment activities (a bi-stability of chromatin state).

Second, cluster 13 (FHL1) is positively correlated with cluster 7 but cluster 14 (histone occupancy) is negatively associated with the same cluster. In cluster 7, both HATs (GCN5 and ESA1) and histone modifications for active genes (e.g., H3K4me3, H3K36me3, H3K9ac, and H3K14ac [26]) are found. Therefore, the gene transcription rate is negatively correlated with the nucleosome density but positively associated with the binding of FHL1 (may recruit HATs) to the promoter region.

Third, for histone crosstalk, cluster 6 is only connected with cluster 7 and cluster 2. In cluster 6, there are three histone modifications (H4ac, H3K4me2, H3K79me3), which are less associated with the transcription rate than H3K9ac and H3K14ac [26]. In cluster 2, three of five proteins are subunits of a HIR complex (e.g., HIR1, HIR2, HIR3), a nucleosome assembly complex that contributes to nucleosome formation. Based on the BioGRID database, HIR complexes directly interact with at least 25 proteins (e.g., IES3, ASF1, ARP8, SNF5, SWI3) that are involved in chromatin remodeling, and 9 another proteins that are involved in histone modification (e.g., LEO1, ESA1, SAS2; for detailed information please refer to Additional file 6). Thus, at the end of a protein-histone modification interaction network, protein involved in chromatin remodeling, such as ATP-dependent chromatin remodelers, may play a key role in generating new histone modifications, e.g., modification in the chromatin structure that may influence gene activity either positively or negatively [14].

**Figure 5:**
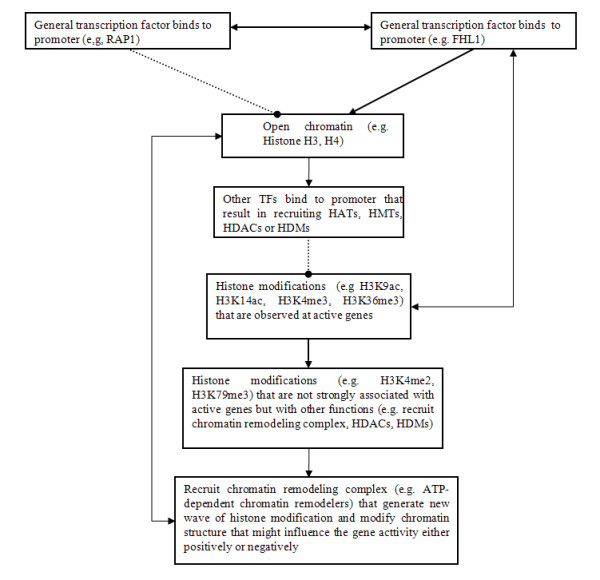
**A simplified diagram of a protein-histone modification interaction network for functional TF binding**. Black smooth line with arrow is positive interaction, black dashed line with circle is negative interaction; histone acetyltransferases - HATs, histone deacetylases - HDACs, histone methyltransferases - HMTs, histone demethylases - HDMs.

Finally, from the inferred networks (Figure 3), a simplified diagram for protein-histone modification interactions can be drawn (Figure 5), where there are several feedback loops which match the description of providing bi-stability and robustness in signaling pathway rather than the regulation by combinatory histone modifications [53]. Thus, the data suggests that a signaling pathway model [22] (Figure 5) may be more suited than the histone code [45] to interpret the function of histone modifications in genome regulation. Overall, the present study opens a new avenue for studying protein-DNA interactions; histone modification levels at the ORFs under different conditions may tell us whether a true protein-DNA binding has occurred [41], which differs significantly with the previous works [8,9,11].

## Competing interests

The author declares he has no competing interests.

## Authors' contributions

JW conceived and designed the study, implemented program, performed data analysis, interpreted results and drafted manuscript.

## Supplementary Material

Additional file 1 **AddFile1_supplementary.doc **Word files. Supplementary information to the paper. Here we provide all supplementary figures and tables to the paper. Click here for file

Additional file 2 **AddFile2_results_of_T-test_vs_RanksSum_test.xls **Excel files. Results of t-test and rank-sum test. Here contains heat-maps of log10 transformed P-values computed by both student T-test and Rank-sum test based on the same datasets (e.g. 11 histone acetylation levels from Kurdistani SK et al. for probes binding and coupling verse probes binding but not coupling). Click here for file

Additional file 3 **AddFile3_STAMP_output.pdf **Acrobat files. Motif analysis by using STAMP. Here contains motif similarity matches by using STAMP tool, and 28 of 32 yeast TFs from Figure 2 are compared because of the availability of their consensus sequence motifs in SGD database. From the comparison, we find the consensus sequences of majority TFs are dissimilar. For example, only 6 (GCN4, BAS1; CAD1, YAP6; STE12, DIG1) out of 28 TFs may have a similar binding motif (E value <0.00001) within the same dataset. However, the length of the similar consensus sequence motifs is quite different, which suggest that *in vivo *binding affinities of those TFs are different. This is because the variation of a nucleotide in either TF recognition sequence or flanking sites could result in a dramatic change in TF binding energy. It is more clearly illustrated by Additional file 1 Figure S2, in which for a pair of TFs with similar consensus sequence motif there are different genome-wide binding patterns (e.g. clustered yeast ChIP-chip ratios). Click here for file

Additional file 4 **AddFile4_18clusters_orf_functional.zip **ZIP files. Protein clustering for functional binding target. Here contains results (18clusters_orf_function.html) of 18 clusters for functional binding sites. Click here for file

Additional file 5 **AddFile5_18clusters_orf_nonfunctional.zip **ZIP files. Protein clustering for non-functional binding target. Here contains results (8clusters_orf_unfunction.html) of 18 clusters for non-functional binding sites. Click here for file

Additional file 6 **AddFile6_Clusters_of_functionalBindingTF_BioGRid_protein_protein_interactions.xls **Excel files. Results from BioGrid database. Here contains protein-protein interactions that extracted from BioGrid database for clusters of functional binding target. Click here for file
