# Supplementary material for: Computational study of associations between histone modification and protein-DNA binding in yeast genome by integrating diverse information
Source: BMC Genomics. 2011 Apr 1;12:172. doi: 10.1186/1471-2164-12-172 (PMC3082246; doi:10.1186/1471-2164-12-172)
Supplement: Additional file 1 — AddFile1_supplementary.doc Word files. Supplementary information to the paper. Here we provide all supplementary figures and tables to the paper. [file 1471-2164-12-172-S1.DOC]

**Supplementary information to “Computational study of associations between histone modification and protein-DNA binding in yeast genome by integrating diverse information”**

**Junbai Wang**

**Supplementary figure S1. Hierarchical clustering of t-values that obtained from the t-test of acetylation levels on 11 histone lysines (Kurdistani SK et al.) in the promoter region (intergenic region).** For 12 yeast TFs in both functional binding sites (bind and couple) and nonfunctional binding sites (bind not couple).

**
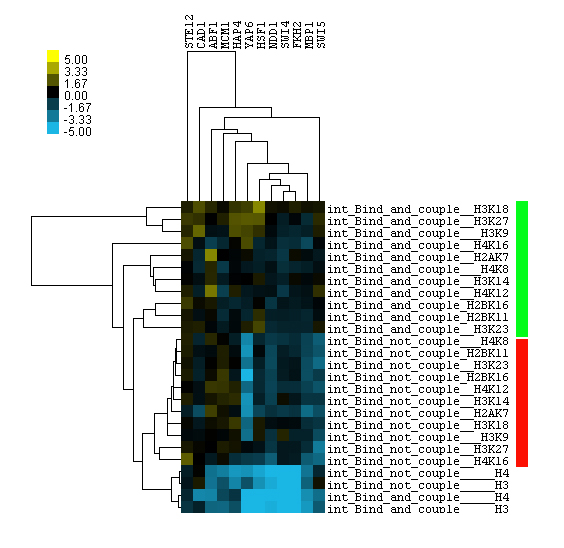
**

**Supplementary figure S2. Hierarchical clustering of log2 transformed genome-wide ChIP-chip ratios^4^ for 6 yeast TFs.** Here each pair of TFs with a similar consensus sequence motif (E value <0.00001) were identified by STAMP tool (results from Additional file 3).


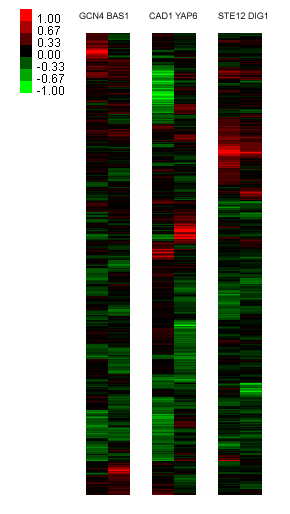


**Supplementary figure S3. Hierarchical clustering of t-values that obtained from the t-test of 8 histone modifications (Pokholok et al.) in the promoter region (intergenic region).** For 32 yeast TFs in both functional binding sites (bind and couple) and nonfunctional binding sites (bind not couple).

**
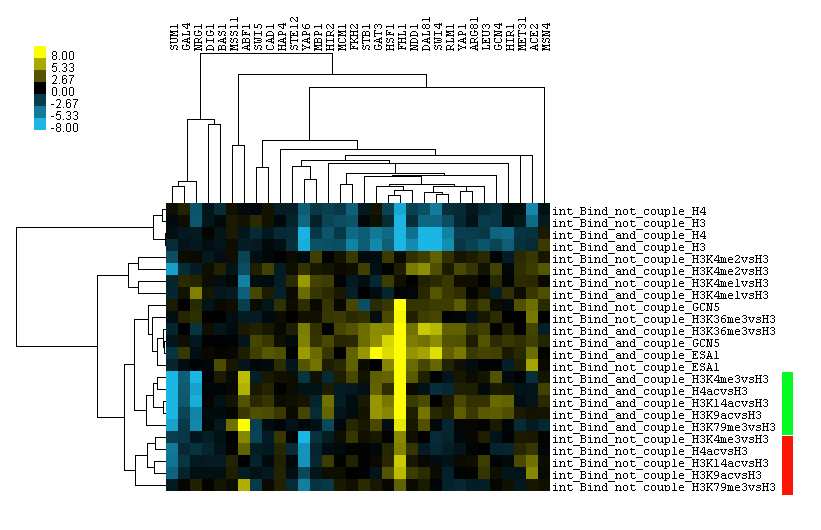
**

**Supplementary figure S4. A protein-histone modification interaction network was predicted by applying Gaussian graphical models (p<0.003) on the centers of 18 clusters.** Each cluster represents a group of proteins that share similar t-values (results of t-tests of nonfunctional binging sites vs. the rest of binding sites for 203 yeast TFs in promoter plus 8 histone modifications in ORF - Pokholok et al.) across 32 yeast TFs: blue colored texts are representative proteins of each cluster, red colored number on each edge is partial correlation coefficient between two vertexes.

**
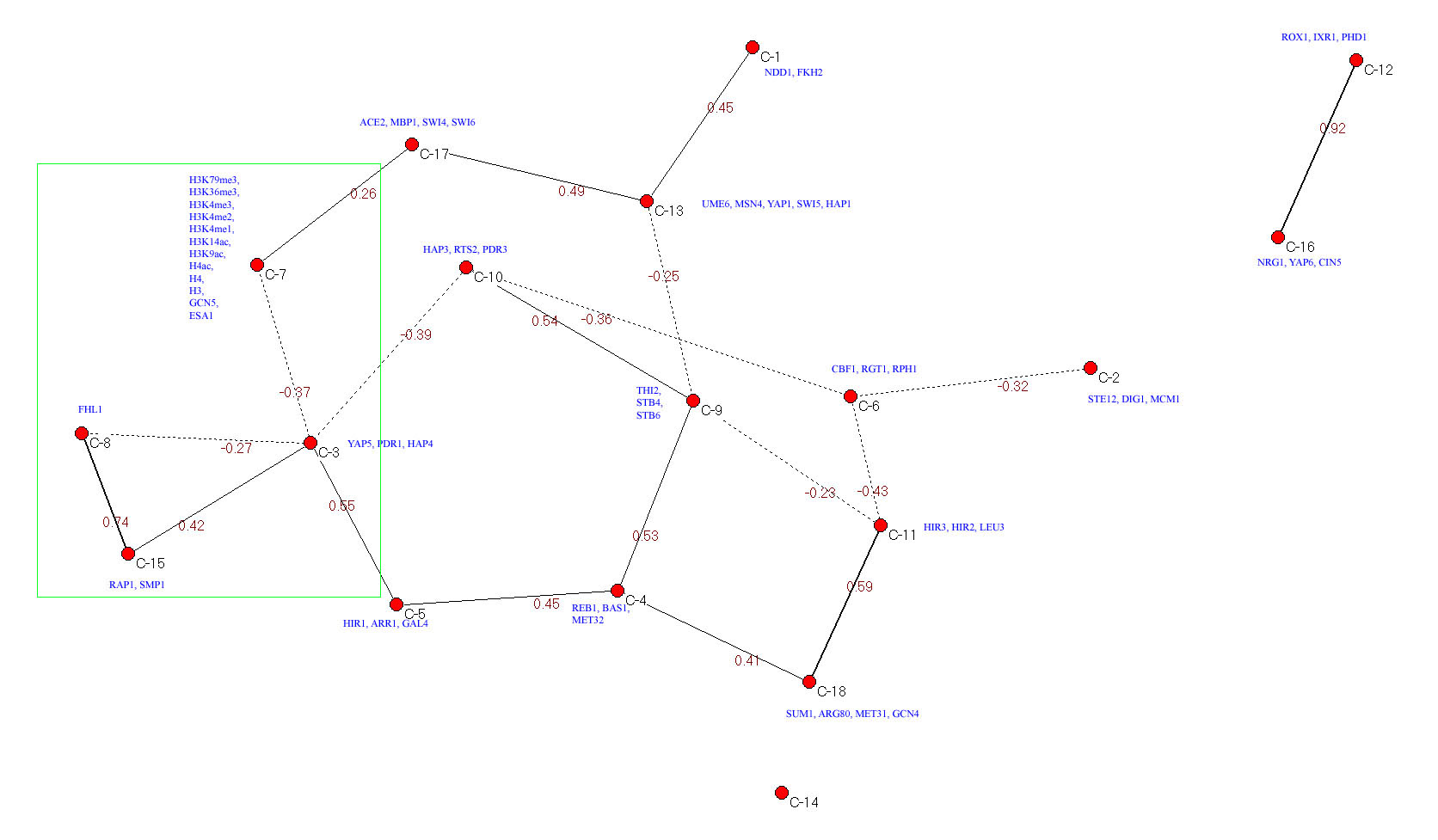
**

**Supplementary figure S5a. Mean confusion matrix and mean percentage of correct classifications for 21 yeast TFs.** The calculations are completed by applying Bayesian Neural Networks on 10 randomly selected test datasets (the half-available binding sites), which include only 11 histone acetylation levels and nucleosome occupancy (Kurdistani et al.)

**
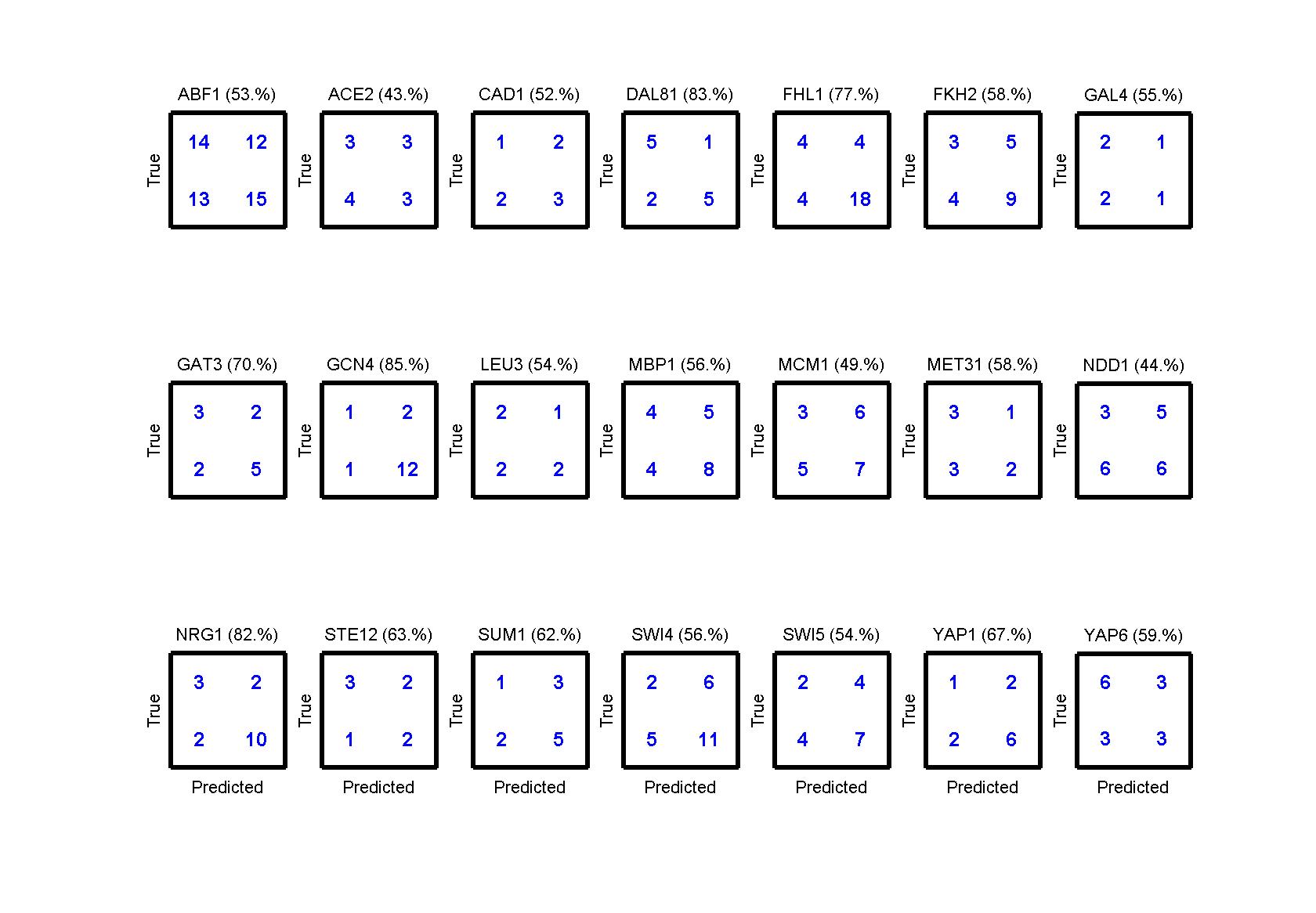
**

**Supplementary figure S5b. Mean confusion matrix and mean percentage of correct classifications for 11 yeast TFs**. Here Bayesian Neural Networks was applied on 11 histone acetylation levels (Kurdistani et al.) and nucleosome occupancy that have sufficient test data (e.g. at least 10 probes for both functional and non-functional binding targets) for performing the 10-fold cross validation analysis.

**
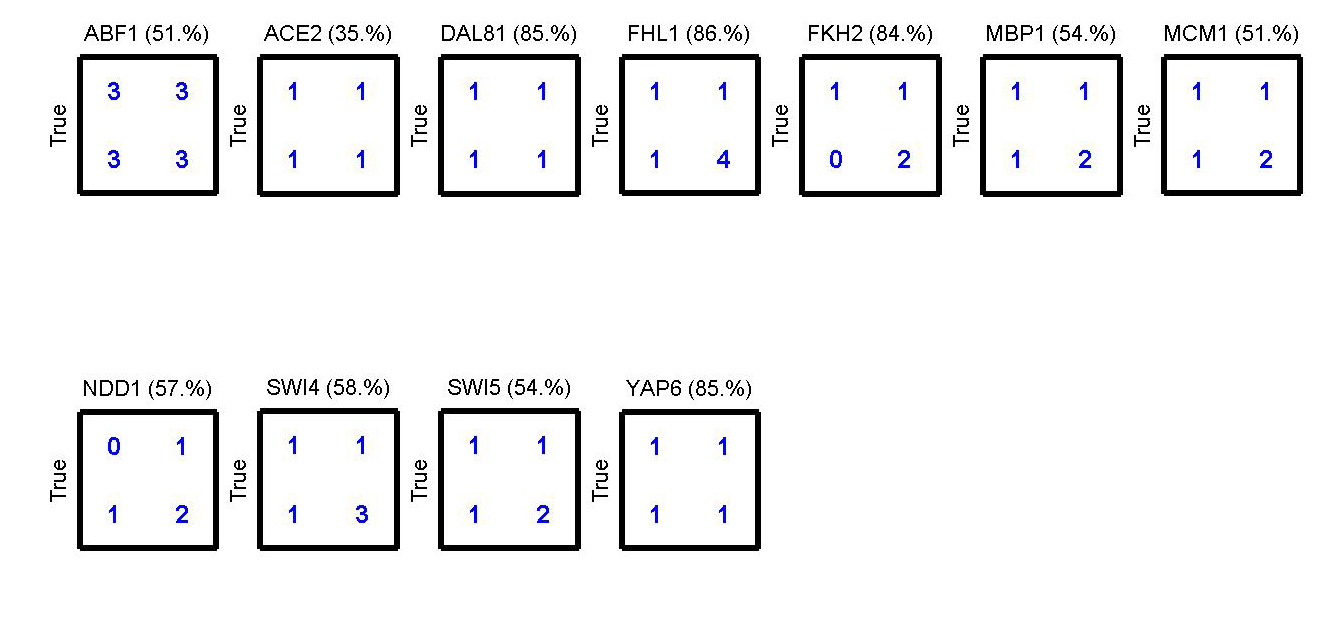
**

**Supplementary figure S6a. Mean confusion matrix and mean percentage of correct classifications for 32 yeast TFs**. The calculations are completed by applying Bayesian Neural Networks on 10 randomly selected test datasets (the half-available binding sites), which include diverse histone modifications (e.g. 3 histone acetylation, 5 histone methylation levels, nucleosome occupancy, and 2 histone acetyltransferase; Pokholok et al.)

**.
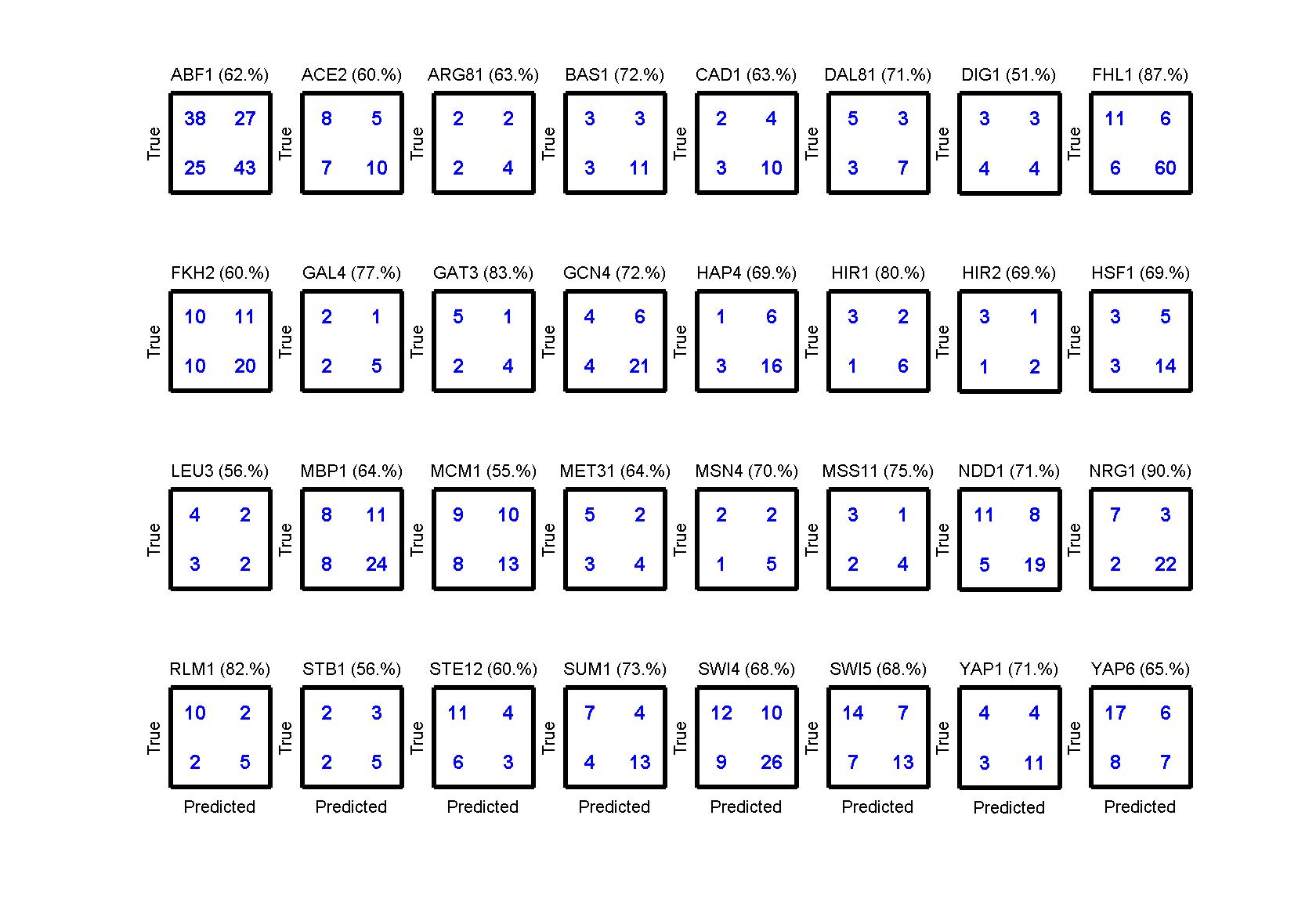
**

**Supplementary figure S6b. Mean confusion matrix and mean percentage of correct classifications for 24 yeast TFs.** Here Bayesian Neural Networks was applied on diverse histone modifications (e.g. 3 histone acetylation, 5 histone methylation levels, nucleosome occupancy, and 2 histone acetyltransferase; Pokholok et al.) that have sufficient test data (e.g. at least 10 probes for both functional and non-functional binding targets) for performing the 10-fold cross validation analysis.


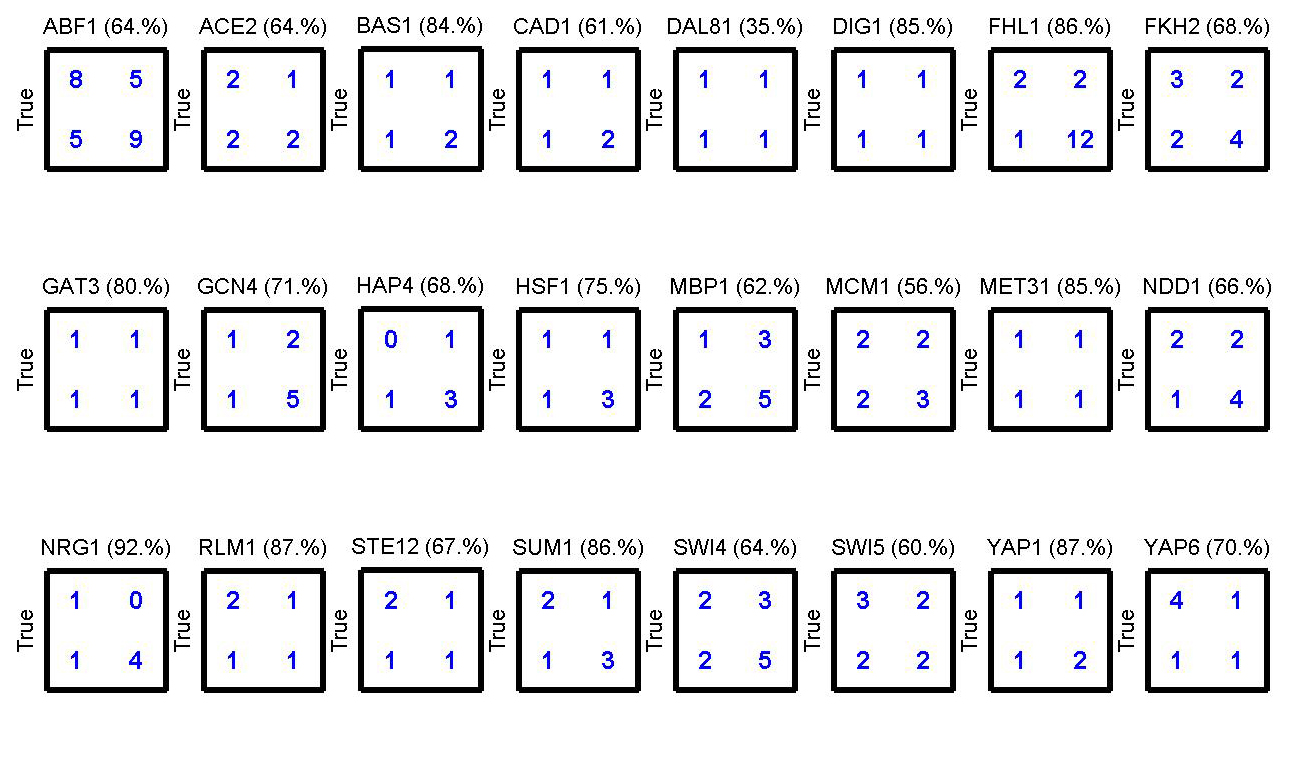


**Supplementary figure S7. A statistical measure of the difference of histone modifications between functional binding sites (bind and couple) and non-functional ones (bind not couple).** Here each subplot is based on the T-values from Figures (1, 2) and supplementary Figures (S1, S3), respectively. Basically, we first took the mean of T-values within each subgroup, then computed correlation coefficient r (and P-values) between the two mean T-values (e.g. bind and couple group vs. bind not couple group). The same calculations were done for the histone modifications at both ORF region and the promoter region. For dataset of Kurdistani et al, we used 11 histone acetylation levels, while for Pokholok et al. we considered 8 histone modifications and 2 histone acetyltransferase. Figure S7 clearly shows that the difference between functional and non-functional binding sites is much stronger at ORF region (e.g. Pokholok et al. r=-0.099, P=0.59) than at the promoter (e.g. Pokholok et al. r=0.74, P=1.3e-06) by considering more diverse histone modification information.

**
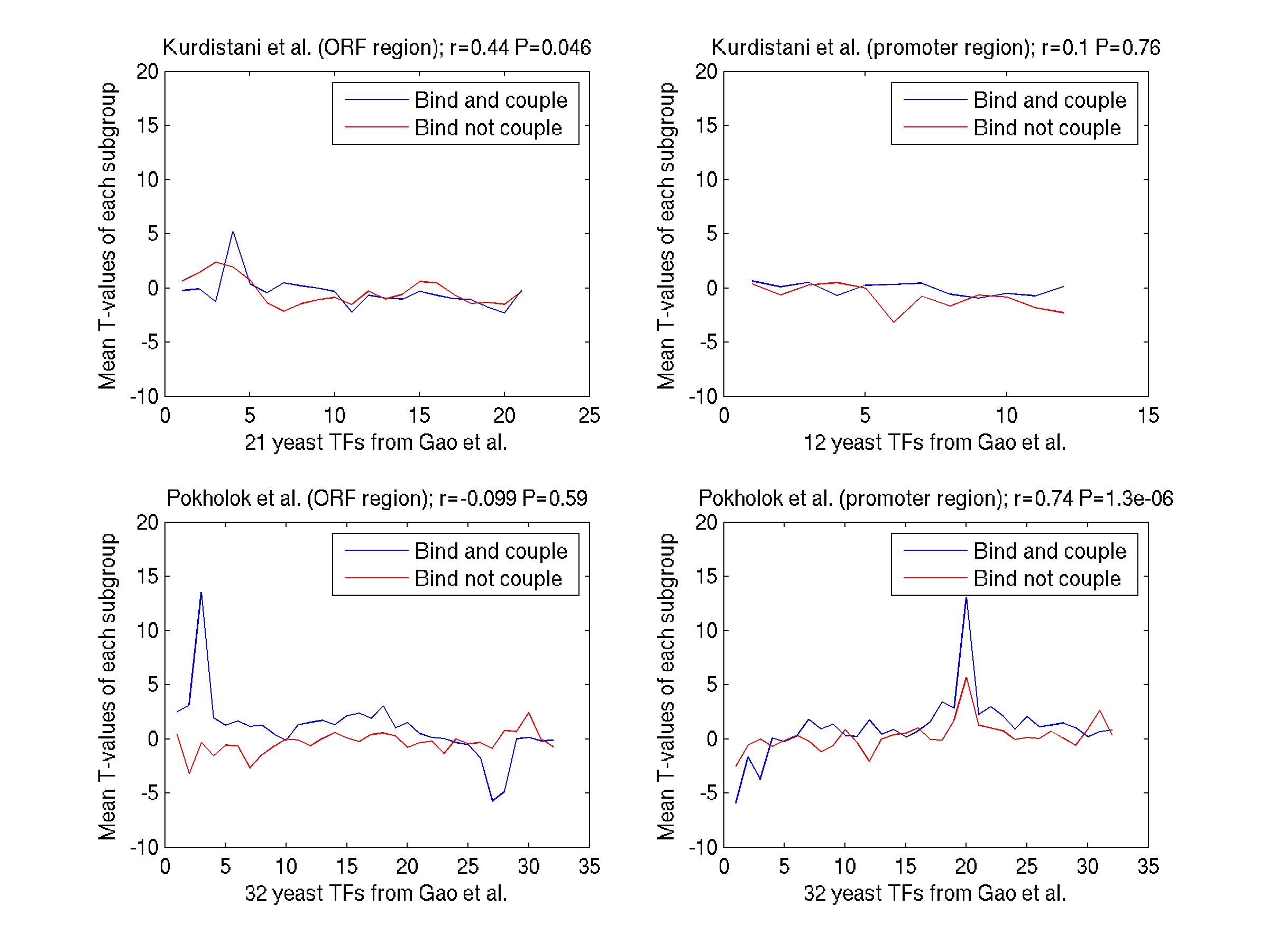
**

**Supplementary figure S8. Hierarchical clustering of t-values obtained from the t-test of acetylation levels on 11 histone lysines (Kurdistani SK et al.) in the coding region (ORF)**. For 9 yeast TFs (the cell cycle TFs were excluded) in both functional binding sites (bind and couple) and non-functional binding sites (bind not couple).

**
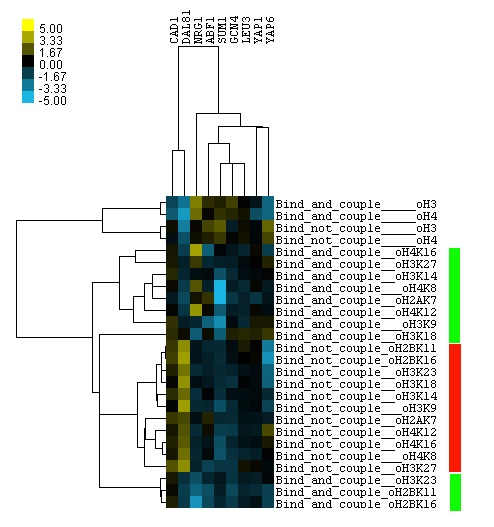
**

**Supplementary figure S9. Hierarchical clustering of t-values obtained from the t-test of 8 histone modifications (Pokholok et al.) in the gene coding region (ORF).** For 13 yeast TFs (the cell cycle TFs were excluded) in both functional binding sites (bind and couple) and non-functional binding sites (bind not couple).

**
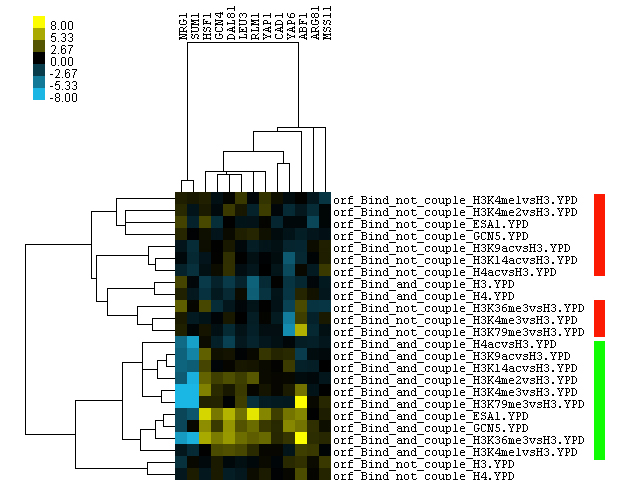
**

**Supplementary Table S1a. Mean percentage of correct classifications and mean total number of correct classifications for 21 yeast TFs.**

| TF Name | Mean percentage of correct classifications on 10 test datasets | Mean total number of correct classifications on 10 test datasets |
| --- | --- | --- |
| *GCN4* | 85 | 12 |
| *DAL81* | 83 | 10 |
| *NRG1* | 82 | 13 |
| **FHL1** | 77 | 22 |
| **GAT3** | 70 | 7 |
| *YAP1* | 67 | 6 |
| **STE12** | 63 | 5 |
| *SUM1* | 62 | 6 |
| *YAP6* | 59 | 8 |
| **FKH2** | 58 | 11 |
| **MET31** | 58 | 4 |
| **MBP1** | 56 | 11 |
| **SWI4** | 56 | 13 |
| **GAL4** | 55 | 3 |
| *LEU3* | 54 | 3 |
| **SWI5** | 54 | 9 |
| *ABF1* | 53 | 28 |
| *CAD1* | 52 | 4 |
| **MCM1** | 49 | 10 |
| **NDD1** | 44 | 8 |
| **ACE2** | 43 | 6 |

The calculations are completed by applying Bayesian Neural Networks on 10 randomly selected test datasets (the half-available binding sites), which include only 11 histone acetylation levels and nucleosome occupancy (Kurdistani et al.); TF name with bold text represents yeast cell cycle related TFs^5^.

**Supplementary Table S1b. Mean percentage of correct classifications and mean total number of correct classifications for 11 yeast TFs.**

| TF name | Mean percentage of correct classifications on 10-fold cross validation | Mean total number of correct classifications on 10-fold cross validation | Number of functional binding sites | Number of nonfunctional binding sites | Total number of binding sites |
| --- | --- | --- | --- | --- | --- |
| **FHL1** | 86 | 5 | 43 | 14 | 57 |
| DAL81 | 85 | 2 | 12 | 10 | 22 |
| YAP6 | 85 | 2 | 10 | 17 | 27 |
| **FKH2** | 84 | 3 | 25 | 15 | 40 |
| **SWI4** | 58 | 3 | 31 | 15 | 46 |
| **NDD1** | 57 | 2 | 22 | 14 | 36 |
| **MBP1** | 54 | 2 | 23 | 16 | 39 |
| **SWI5** | 54 | 2 | 20 | 11 | 31 |
| ABF1 | 51 | 6 | 55 | 51 | 106 |
| **MCM1** | 51 | 2 | 22 | 17 | 39 |
| **ACE2** | 35 | 1 | 14 | 11 | 25 |

Here Bayesian Neural Networks was applied on 11 histone acetylation levels and nucleosome occupancy (Kurdistani et al.) that have sufficient test data (e.g. at least 10 probes for both functional and non-functional binding targets) for performing the 10-fold cross validation analysis; TF name with bold text represents yeast cell cycle related TFs^5^.

**Supplementary Table S2. Mean percentage of correct classifications and mean total number of correct classifications for 24 yeast TFs.**

| TF name | Mean percentage of correct classifications on 10-fold cross validation | Mean total number of correct classifications on 10-fold cross validation | Number of functional binding sites | Number of nonfunctional binding sites | Total number of binding sites |
| --- | --- | --- | --- | --- | --- |
| NRG1 | 92 | 5 | 47 | 19 | 66 |
| RLM1 | 87 | 3 | 13 | 22 | 35 |
| YAP1 | 87 | 3 | 26 | 15 | 41 |
| **FHL1** | 86 | 14 | 131 | 32 | 163 |
| SUM1 | 86 | 5 | 33 | 21 | 54 |
| **DIG1** | 85 | 2 | 15 | 11 | 26 |
| **MET31** | 85 | 2 | 15 | 13 | 28 |
| **BAS1** | 84 | 3 | 26 | 11 | 37 |
| **GAT3** | 80 | 2 | 11 | 10 | 21 |
| HSF1 | 75 | 3 | 32 | 14 | 46 |
| GCN4 | 71 | 5 | 51 | 21 | 72 |
| YAP6 | 70 | 5 | 28 | 44 | 72 |
| **FKH2** | 68 | 7 | 59 | 40 | 99 |
| **HAP4** | 68 | 3 | 37 | 12 | 49 |
| **STE12** | 67 | 2 | 17 | 28 | 45 |
| **NDD1** | 66 | 5 | 46 | 36 | 82 |
| ABF1 | 64 | 16 | 135 | 129 | 264 |
| **ACE2** | 64 | 4 | 32 | 25 | 57 |
| **SWI4** | 64 | 7 | 68 | 42 | 110 |
| **MBP1** | 62 | 6 | 63 | 37 | 100 |
| CAD1 | 61 | 2 | 24 | 11 | 35 |
| **SWI5** | 60 | 5 | 39 | 40 | 79 |
| **MCM1** | 56 | 4 | 41 | 37 | 78 |
| DAL81 | 35 | 1 | 19 | 14 | 33 |

Here Bayesian Neural Networks was applied on diverse histone modifications (e.g. 3 histone acetylation, 5 histone methylation levels, nucleosome occupancy, and 2 histone acetyltransferase; Pokholok et al.) that have sufficient test data (e.g. at least 10 probes for both functional and non-functional binding targets) for performing the 10-fold cross validation analysis; TF name with bold text represents yeast cell cycle related TFs^5^.

**Additional File 2**

(**AddFile2_results_of_T-test_vs_RanksSum_test.xls**) contains heat-maps of log10 transformed P-values computed by both student T-test and RankSum test based on the same datasets (e.g. 11 histone acetylation levels from Kurdistani SK et al. for probes binding and coupling verse probes binding but not coupling).

**Additional File 3**

(**AddFile3_STAMP_output.pdf**) contains motif similarity matches by using STAMP^1^ tool. Here 28 of 32 yeast TFs from Figure 2 are compared because of the availability of their consensus sequence motifs in SGD database. From the comparison, we find the consensus sequences of majority TFs are dissimilar. For example, only 6 (GCN4, BAS1; CAD1, YAP6; STE12, DIG1) out of 28 TFs may have a similar binding motif (E value <0.00001) within the same dataset. However, the length of the similar consensus sequence motifs is quite different, which suggest that *in vivo* binding affinities of those TFs are different. This is because the variation of a nucleotide in either TF recognition sequence or flanking sites could result in a dramatic change in TF binding energy^2,3^. It is more clearly illustrated by supplementary Figure S2, in which for a pair of TFs with similar consensus sequence motif there are different genome-wide binding patterns (e.g. clustered yeast ChIP-chip ratios^4^).

**Additional File 4** (**AddFile4_18clusters_orf_functional.zip**) contains results (18clusters_orf_function.html) of 18 clusters for functional binding sites.

**Additional File 5** (**AddFile5_18clusters_orf_nonfunctional.zip**) contains results (8clusters_orf_unfunction.html) of 18 clusters for non-functional binding sites.

**Additional File 6** (**AddFile6_Clusters_of_functionalBindingTF_BioGRid_protein_protein_interactions.xls**) contains protein-protein interactions that extracted from BioGrid database for clusters of functional binding sites.

**Reference:**

1. STAMP: a web tool for exploring DNA-binding motif similarities.

Mahony S, Benos PV. Nucleic Acids Res. 2007 Jul; 35(Web Server issue):W253-8.

2. Protein-DNA recognition complexes: conservation of structure and binding energy in the transition state.

Jen-Jacobson L. Biopolymers 1997, 44(2):153-180.

3. Additivity in protein-DNA interactions: how good an approximation is it?

Benos PV, Bulyk ML, Stormo GD. Nucleic acids research 2002, 30(20):4442-4451.

4. Transcriptional regulatory code of a eukaryotic genome.

Harbison CT, Gordon DB, Lee TI, Rinaldi NJ, Macisaac KD, Danford TW, Hannett NM, Tagne JB, Reynolds DB, Yoo J, et al. Nature 2004 , 431(7004):99-104.

5. Statistical methods for identifying yeast cell cycle transcription factors.

Tsai HK, Lu HH, Li WH. Proceedings of the National Academy of Sciences of the United States of America 2005, 102(38):13532-13537.
