# Supplementary material for: Computational study of associations between histone modification and protein-DNA binding in yeast genome by integrating diverse information
Source: BMC Genomics. 2011 Apr 1;12:172. doi: 10.1186/1471-2164-12-172 (PMC3082246; doi:10.1186/1471-2164-12-172)
Supplement: Additional file 3 — AddFile3_STAMP_output.pdf Acrobat files. Motif analysis by using STAMP. Here contains motif similarity matches by using STAMP tool, and 28 of 32 yeast TFs from Figure 2 are compared because of the availability of their consensus sequence motifs in SGD database. From the comparison, we find the consensus sequences of majority TFs are dissimilar. For example, only 6 (GCN4, BAS1; CAD1, YAP6; STE12, DIG1) out of 28 TFs may have a similar binding motif (E value <0.00001) within the same dataset. However, the length of the similar consensus sequence motifs is quite different, which suggest that in vivo binding affinities of those TFs are different. This is because the variation of a nucleotide in either TF recognition sequence or flanking sites could result in a dramatic change in TF binding energy. It is more clearly illustrated by Additional file 1 Figure S2, in which for a pair of TFs with similar consensus sequence motif there are different genome-wide binding patterns (e.g. clustered yeast ChIP-chip ratios). [file 1471-2164-12-172-S3.PDF]

Jump to: Multiple Alignment Motif Tree Motif Matching
Input file: 28 motifs loaded
Settings: Metric=PCC, Alignment=SWU, Gap-open=1000, Gap-extend=1000, -nooverlapalign
Multiple Alignment=IR, Tree=UPGMA, Matching against: user-defined
Note: All results files are removed nightly at midnight EST. Please save your results by saving "Webpage, complete".
Download results as a PDF
Click here to run STAMP again.

Multiple Alignment

(Consensus sequence representations shown, but multiple alignment was carried out on the matrices)

```
abf1:     ----------RTCAYTNNNNACG------
ace2:     ----------ACCAGC-------------
bas1:     --------GAGTCA---------------
cad1:     ------MTTASTMAKC-------------
dal81:    AAACGCAATTTTC----------------
dig1:     -------TGTTTCA---------------
fhl1:     ----CAYCCRTACA---------------
fkh2:     --------TRTTKRY--------------
gal4:     ------------CGGNNNNNNNNNNNCCG
gat3:     ------GTAGATCY---------------
gcn4:     -------WGAGTCAYT-------------
hap4:     -----KNTNATTGGNNGR-----------
hsf1:     TTCYNNNNNNTTC----------------
leu3:     -----CCGNNNNCGG--------------
mbp1:     ---------ACGCGT--------------
mcm1:     ---------WTWCCYAAWNNGGTAA----
met31:    ----CCACAGTTT----------------
msn4:     ------CCCCTT-----------------
ndd1:     -----CCNNWTYNGG--------------
nrg1:     -------AGGGTCC---------------
rlm1:     ----CTAWWWWTAG---------------
stb1:     ----------TTSGCGTYY----------
ste12:    -------TGTTTCAT--------------
```

```
sum1:    ----WTTWSTGWCRC--------------
swi4:    --------TTTTCGTG-------------
swi5:    -----------YCAGCM------------
yap6:    ----------TTAGTAA------------
yap1:    --------MTTACGTAAK-----------
```

Familial Profile:
(click for matrix)

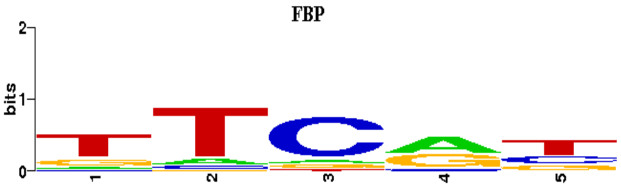

Motif Tree

Tree (drawn by Phylip)
Click here for Newick-format tree (viewable with MEGA)

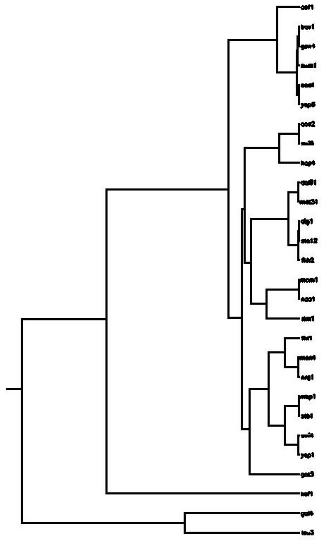

Input Motif	Best match in user-defined
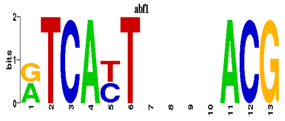 abf1	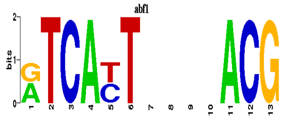 abf1 (E val: 3.4913e-09)
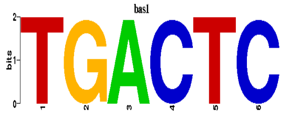 bas1	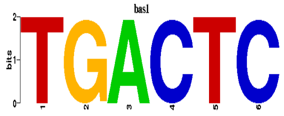 bas1 (E val: 3.8873e-11)
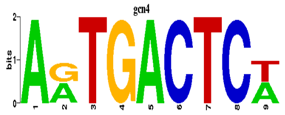 gcn4	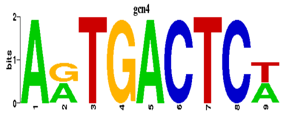 gcn4 (E val: 1.1102e-16)
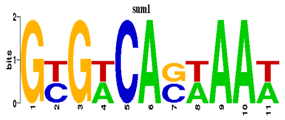 sum1	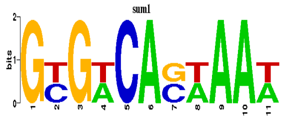 sum1 (E val: 0.0000e+00)

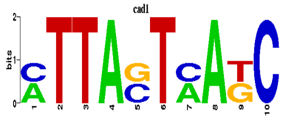 cad1	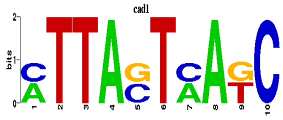 cad1 (E val: 0.0000e+00)
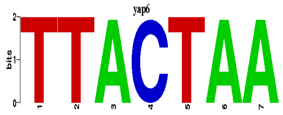 yap6	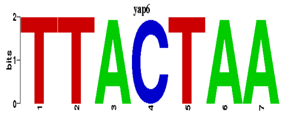 yap6 (E val: 1.0500e-12)
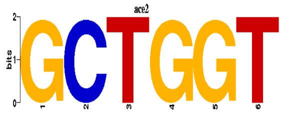 ace2	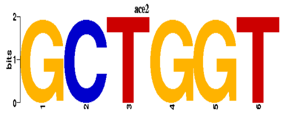 ace2 (E val: 3.8873e-11)
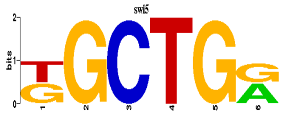 swi5	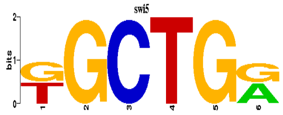 swi5 (E val: 3.8873e-11)
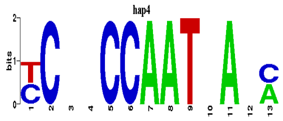 hap4	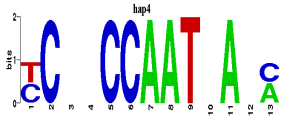 hap4 (E val: 3.4913e-09)
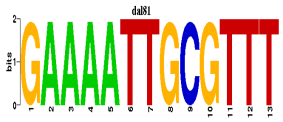 dal81	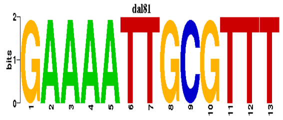 dal81 (E val: 0.0000e+00)
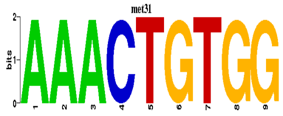 met31	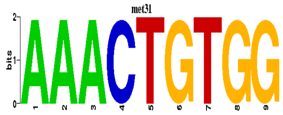 met31 (E val: 1.1102e-16)
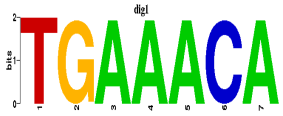 dig1	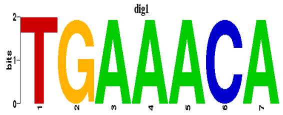 dig1 (E val: 1.0500e-12)

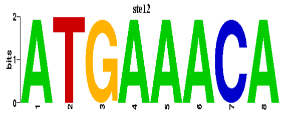 ste12	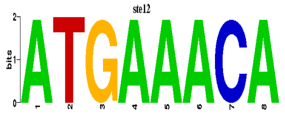 ste12 (E val: 6.5503e-15)
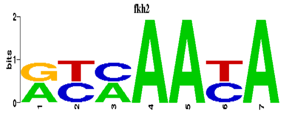 fkh2	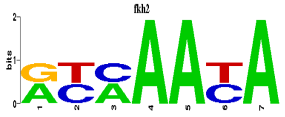 fkh2 (E val: 1.0500e-12)
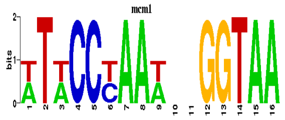 mcm1	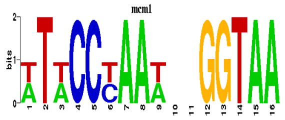 mcm1 (E val: 0.0000e+00)
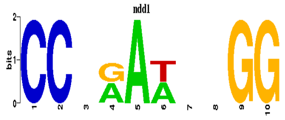 ndd1	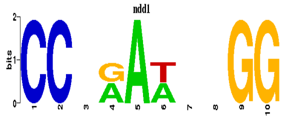 ndd1 (E val: 5.3250e-07)
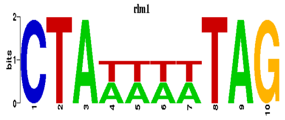 rlm1	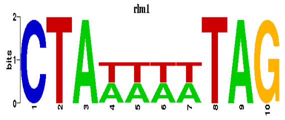 rlm1 (E val: 0.0000e+00)
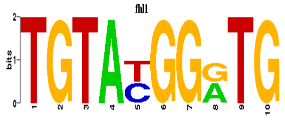 fhl1	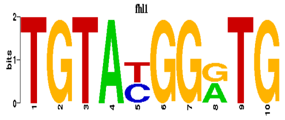 fhl1 (E val: 0.0000e+00)
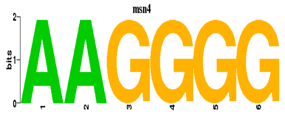 msn4	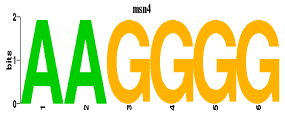 msn4 (E val: 3.8873e-11)
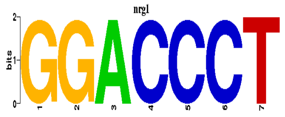 nrg1	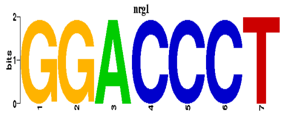 nrg1 (E val: 1.0500e-12)

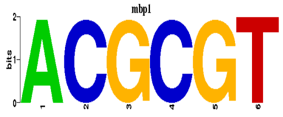 mbp1	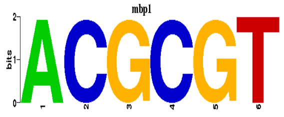 mbp1 (E val: 3.8873e-11)
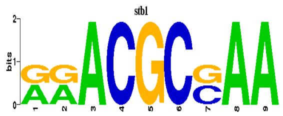 stb1	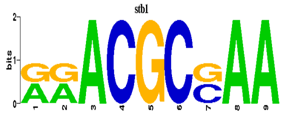 stb1 (E val: 1.1102e-16)
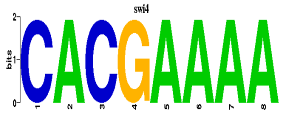 swi4	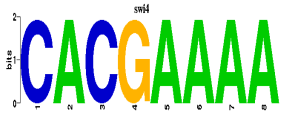 swi4 (E val: 6.5503e-15)
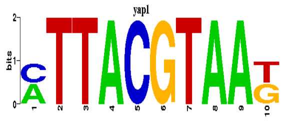 yap1	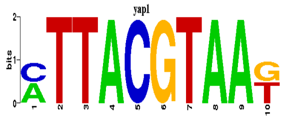 yap1 (E val: 0.0000e+00)
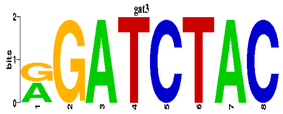 gat3	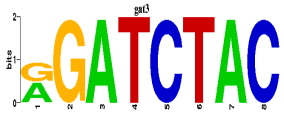 gat3 (E val: 6.5503e-15)
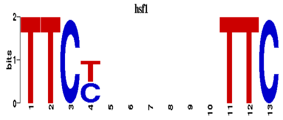 hsf1	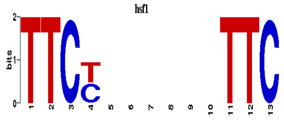 hsf1 (E val: 3.3200e-04)
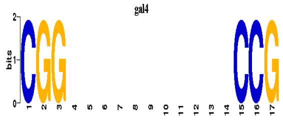 gal4	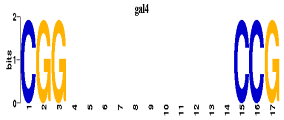 gal4 (E val: 1.5438e-01)
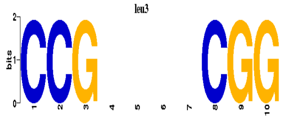 leu3	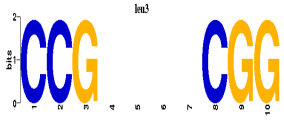 leu3 (E val: 2.0663e-04)

Motif Similarity Matches

abf1

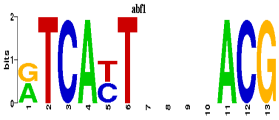 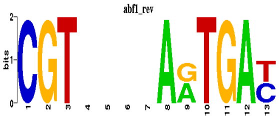

forward *reverse compliment*

Name	*E value*	*Alignment*	*Motif*
abf1	3.4913e-09	CGTNNNNNARTGAY<br>CGTNNNNNARTGAY	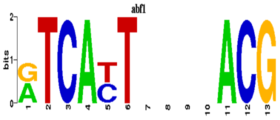
gcn4	2.8258e-03	---RTCAYTNNNNNACG<br>WGAGTCAYT--------	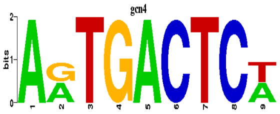
bas1	1.1615e-01	--RTCAYTNNNNNACG<br>GAGTCA----------	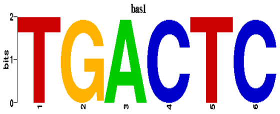
sum1	2.9221e-01	RTCAYTNNNNNACG-<br>---WTTWSTGWCRC	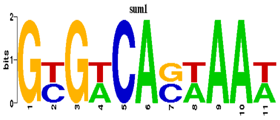
ste12	3.5928e-01	---RTCAYTNNNNNACG<br>TGTTTCAT---------	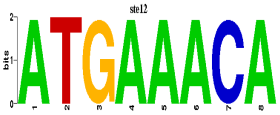

---

bas1

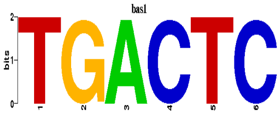 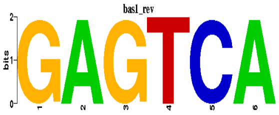

forward *reverse compliment*

Name	*E value*	*Alignment*	*Motif*
bas1	3.8873e-11	GAGTCA<br>GAGTCA	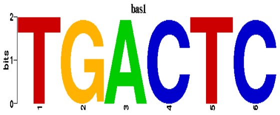

gcn4	1.1768e-08	-GAGTCA--<br>WGAGTCAYT	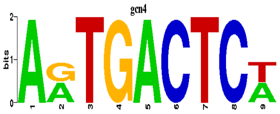
cad1	7.3837e-03	--TGACTC--<br>GMTKASTAAK	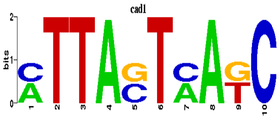
nrg1	1.5524e-02	-GAGTCA<br>AGGGTCC	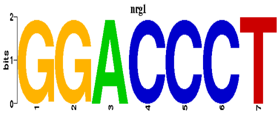
yap6	1.5524e-02	-GAGTCA<br>TTAGTAA	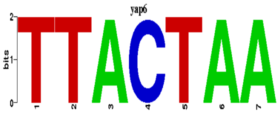

gcn4

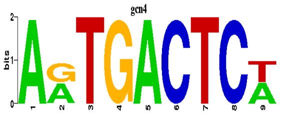 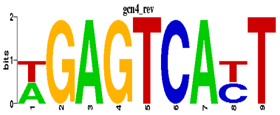

forward *reverse compliment*

Name	*E value*	*Alignment*	*Motif*
gcn4	1.1102e-16	WGAGTCAYT<br>WGAGTCAYT	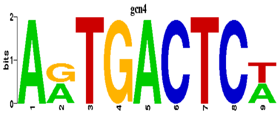
bas1	1.1768e-08	WGAGTCAYT<br>-GAGTCA--	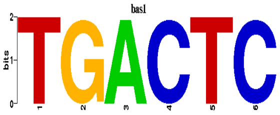
abf1	2.8258e-03	--------ARTGACTCW<br>CGTNNNNNARTGAY---	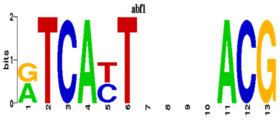
ste12	1.3174e-02	WGAGTCAYT<br>TGTTTCAT-	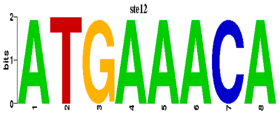
sum1	1.3264e-02	---WGAGTCAYT<br>WTTWSTGWCRC-	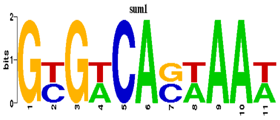

sum1

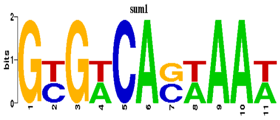 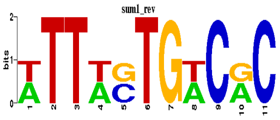

forward *reverse compliment*

Name	*E value*	*Alignment*	*Motif*
sum1	0.0000e+00	WTTWSTGWCRC<br>WTTWSTGWCRC	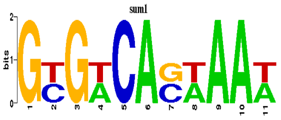
gcn4	1.3264e-02	WTTWSTGWCRC-<br>---WGAGTCAYT	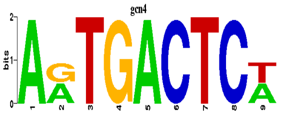
yap6	1.5156e-02	GYGWCASWAAW<br>---TTAGTAA-	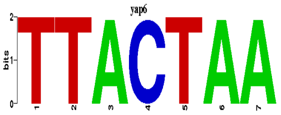
bas1	1.9430e-02	GYGWCASWAAW<br>GAGTCA-----	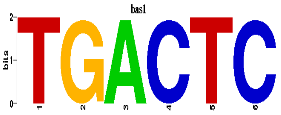
swi4	2.1946e-02	-WTTWSTGWCRC<br>TTTTCGTG----	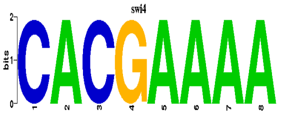

---

cad1

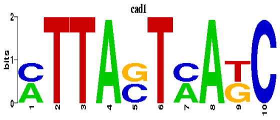 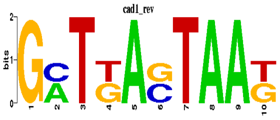

forward *reverse compliment*

Name	*E value*	*Alignment*	*Motif*
cad1	0.0000e+00	GMTKASTAAK<br>GMTKASTAAK	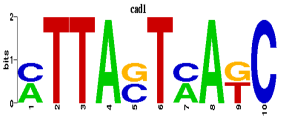
yap6	3.6117e-07	MTTASTMAKC<br>-TTAGTAA--	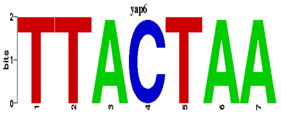

bas1	7.3837e-03	GMTKASTAAK<br>--TGACTC--	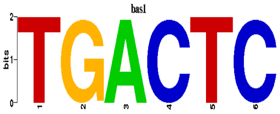
swi5	4.0226e-02	MTTASTMAKC-<br>-----YCAGCM	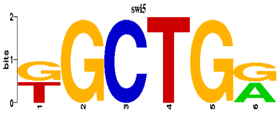
yap1	4.1418e-02	GMTKASTAAK<br>MTTACGTAAK	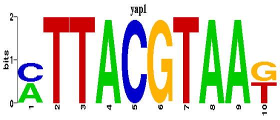

yap6

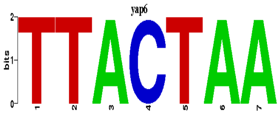

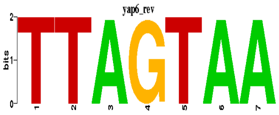

forward *reverse compliment*

Name	*E value*	*Alignment*	*Motif*
yap6	1.0500e-12	TTAGTAA<br>TTAGTAA	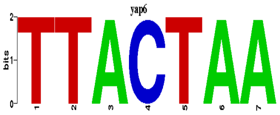
cad1	3.6117e-07	--TTACTAA-<br>GMTKASTAAK	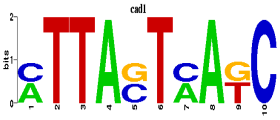
mcm1	8.3785e-03	-TTACTAA--------<br>WTWCCYAAWNNGGTAA	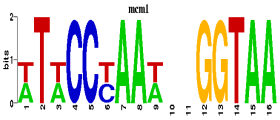
yap1	1.3855e-02	-TTACTAA--<br>MTTACGTAAK	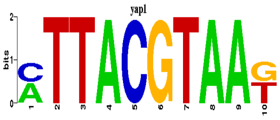
sum1	1.5156e-02	-TTACTAA---<br>WTTWSTGWCRC	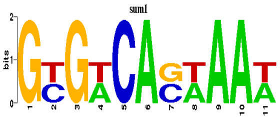

ace2

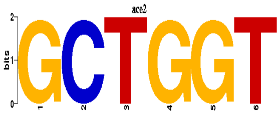

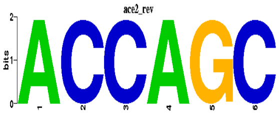

forward *reverse compliment*

Name	*E value*	*Alignment*	*Motif*
ace2	3.8873e-11	ACCAGC<br>ACCAGC	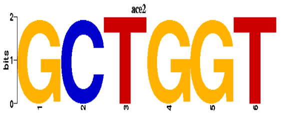
swi5	2.5340e-05	ACCAGC-<br>-YCAGCM	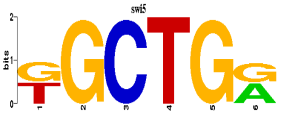
nrg1	1.4681e-01	GCTGGT--<br>-AGGGTCC	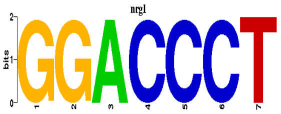
fkh2	1.5612e-01	-GCTGGT<br>TRTTKRY	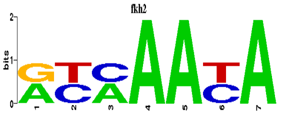
cad1	2.1025e-01	GCTGGT----<br>GMTKASTAAK	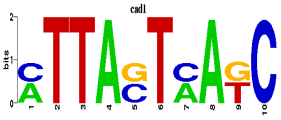

swi5

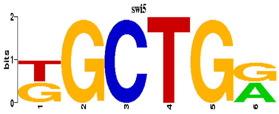

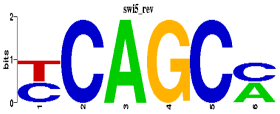

forward *reverse compliment*

Name	*E value*	*Alignment*	*Motif*
swi5	3.8873e-11	YCAGCM<br>YCAGCM	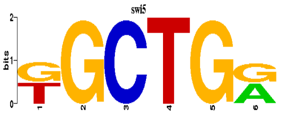
ace2	2.5340e-05	-YCAGCM<br>ACCAGC-	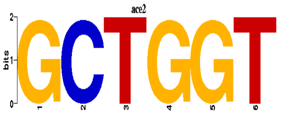
cad1	4.0226e-02	KGCTGR-----<br>-GMTKASTAAK	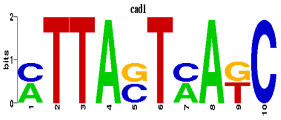

fkh2	4.3464e-02	KGCTGR-<br>TRTTKRY	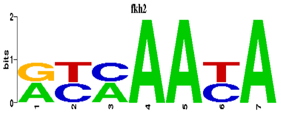
hap4	1.1615e-01	-------KGCTGR<br>KNTNATTGGNNGR	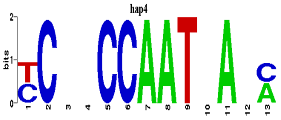

---

hap4

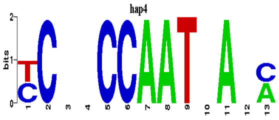

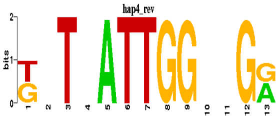

forward *reverse compliment*

Name	*E value*	*Alignment*	*Motif*
hap4	3.4913e-09	KNTNATTGGNNGR<br>KNTNATTGGNNGR	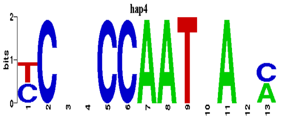
swi5	1.1615e-01	YCNNCCAATNANM<br>YCAGCM-------	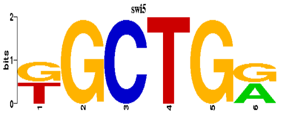
fkh2	1.6844e-01	KNTNATTGGNNGR<br>---TRTTKRY---	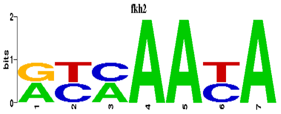
ste12	3.5928e-01	--KNTNATTGGNNGR<br>TGTTTCAT-------	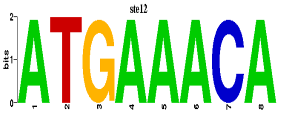
rlm1	3.6255e-01	YCNNCCAATNANM-<br>----CTAWWWWTAG	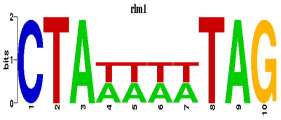

---

dal81

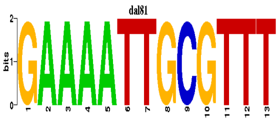

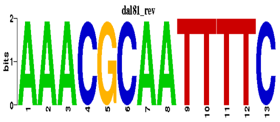

forward *reverse compliment*

Name	*E value*	*Alignment*	*Motif*
dal81	0.0000e+00	AAACGCAATTTTC<br>AAACGCAATTTTC	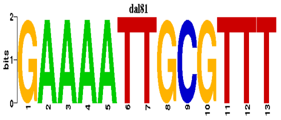
stb1	2.8258e-03	GAAAATTGCGTTT<br>----TTSGCGTYY	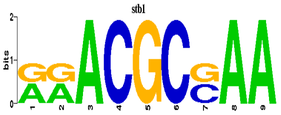
met31	7.3922e-03	AAACGCAATTTTC<br>--CCACAGTTT--	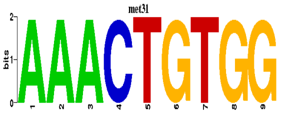
swi4	8.2593e-03	AAACGCAATTTTC---<br>--------TTTTCGTG	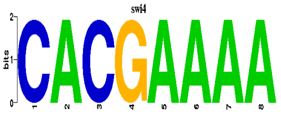
yap1	2.2753e-02	GAAAATTGCGTTT-<br>----MTTACGTAAK	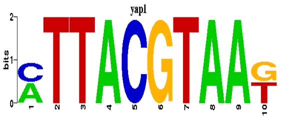

met31

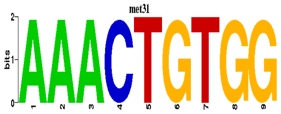

forward

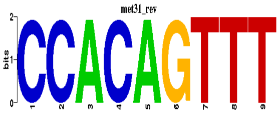

reverse compliment

Name	*E value*	*Alignment*	*Motif*
met31	1.1102e-16	CCACAGTTT<br>CCACAGTTT	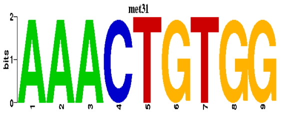
dal81	7.3922e-03	--CCACAGTTT--<br>AAACGCAATTTTC	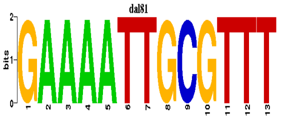
dig1	3.2348e-02	CCACAGTTT--<br>----TGTTTCA	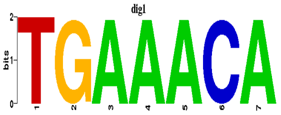
ste12	6.5519e-02	CCACAGTTT---<br>----TGTTTCAT	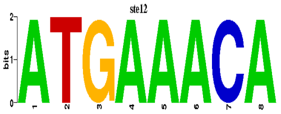

sum1	1.9693e-01	-AAACTGTGG-<br>WTTWSTGWCRC	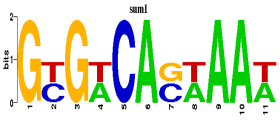

dig1

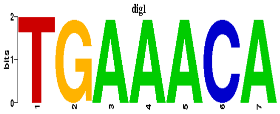

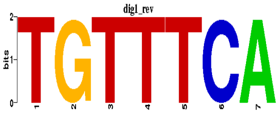

forward *reverse compliment*

Name	*E value*	*Alignment*	*Motif*
dig1	1.0500e-12	TGTTTCA<br>TGTTTCA	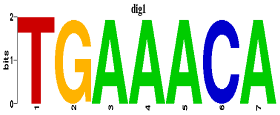
ste12	1.2763e-11	TGTTTCA-<br>TGTTTCAT	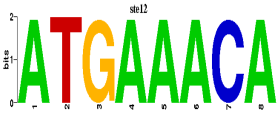
fkh2	5.3262e-03	TGTTTCA<br>TRTTKRY	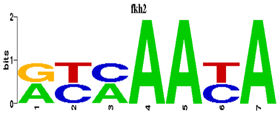
swi4	2.0287e-02	TGTTTCA--<br>-TTTTCGTG	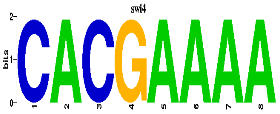
met31	3.2348e-02	----TGTTTCA<br>CCACAGTTT--	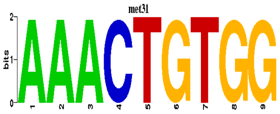

ste12

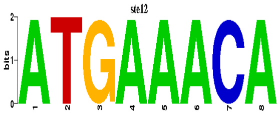

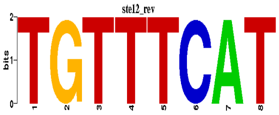

forward *reverse compliment*

Name	*E value*	*Alignment*	*Motif*

ste12	6.5503e-15	TGTTTCAT<br>TGTTTCAT	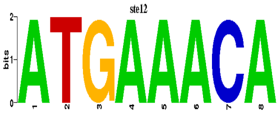
dig1	1.2763e-11	TGTTTCAT<br>TGTTTCA-	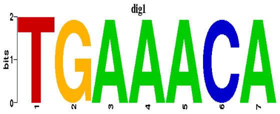
swi4	3.1086e-03	TGTTTCAT-<br>-TTTTCGTG	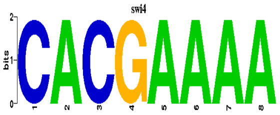
fkh2	1.1121e-02	TGTTTCAT<br>TRTTKRY-	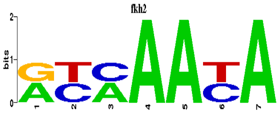
gcn4	1.3174e-02	TGTTTCAT-<br>WGAGTCAYT	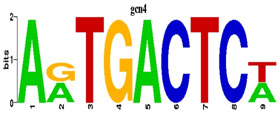

---

fkh2

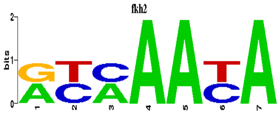 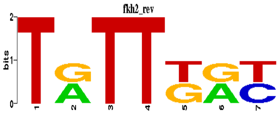

forward *reverse compliment*

Name	*E value*	*Alignment*	*Motif*
fkh2	1.0500e-12	TRTTKRY<br>TRTTKRY	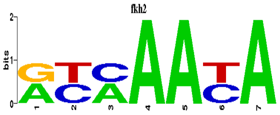
dig1	5.3262e-03	TRTTKRY<br>TGTTTCA	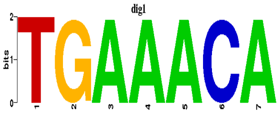
ste12	1.1121e-02	TRTTKRY-<br>TGTTTCAT	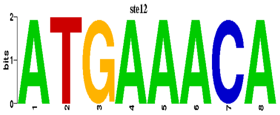
swi5	4.3464e-02	RYMAAYA<br>-YCAGCM	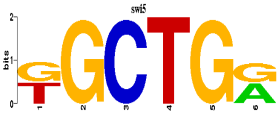

fhl1	4.8339e-02	-RYMAAYA--<br>CAYCCRTACA	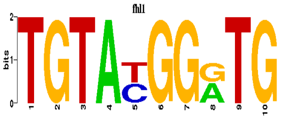

---

mcm1

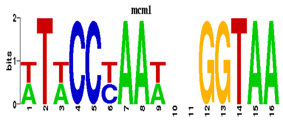 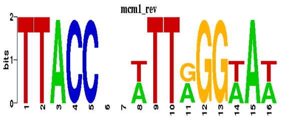

forward *reverse compliment*

Name	*E value*	*Alignment*	*Motif*
mcm1	0.0000e+00	TTACCNNNWTTRGGWAW<br>TTACCNNNWTTRGGWAW	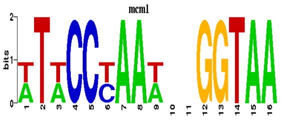
ndd1	2.8137e-04	TTACCNNNWTTRGGWAW<br>---CCNNNWTYNGG---	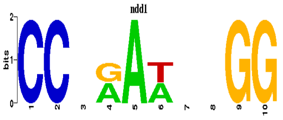
yap6	8.3785e-03	WTWCCYAAWNNGGTAA<br>-TTACTAA--------	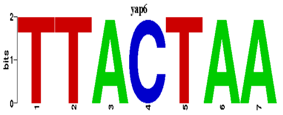
yap1	2.7291e-02	-WTWCCYAAWNNGGTAA<br>MTTACGTAAK-------	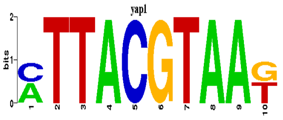
msn4	1.5730e-01	TTACCNNNWTTRGGWAW<br>---CCCCTT-------	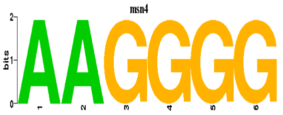

---

ndd1

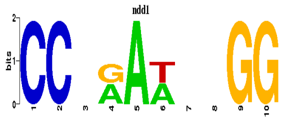 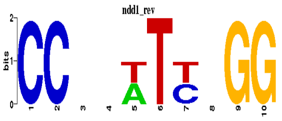

forward *reverse compliment*

Name	*E value*	*Alignment*	*Motif*

ndd1	5.3250e-07	CCNNWTYNGG<br>CCNNWTYNGG	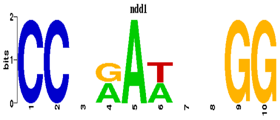
mcm1	2.8137e-04	---CCNNWTYNGG---<br>TTACCNNWTTRGGWAW	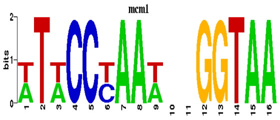
msn4	6.7388e-02	CCNNWTYNGG<br>CCCCTT----	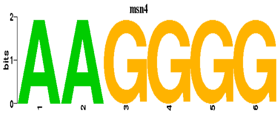
leu3	2.0159e-01	CCNRAWNNGG<br>CCGNNNNCGG	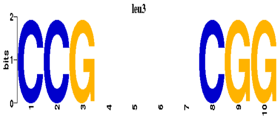
swi4	4.5274e-01	CCNNWTYNGG-<br>---TTTTCGTG	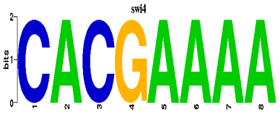

---

rlm1

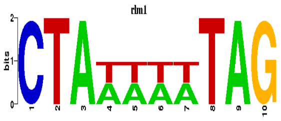

forward

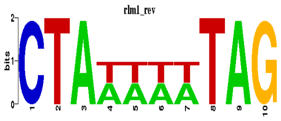

reverse compliment

Name	*E value*	*Alignment*	*Motif*
rlm1	0.0000e+00	CTAWWWWTAG<br>CTAWWWWTAG	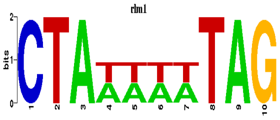
yap6	1.4496e-01	CTAWWWWTAG---<br>------TTAGTAA	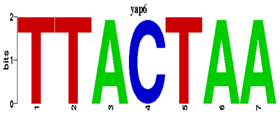
dal81	2.1624e-01	----CTAWWWWTAG<br>AAACGCAATTTTC-	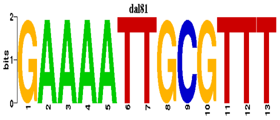
fkh2	2.5682e-01	CTAWWWWTAG<br>--RYMAAYA-	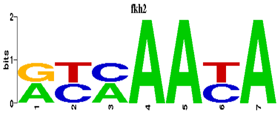
swi4	3.1912e-01	CTAWWWWTAG--<br>----TTTTCGTG	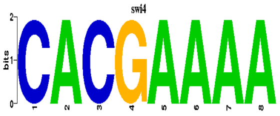

fhl1

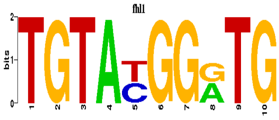 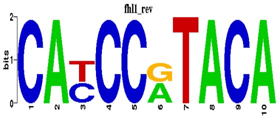

forward *reverse compliment*

Name	*E value*	*Alignment*	*Motif*
fhl1	0.0000e+00	CAYCCRTACA<br>CAYCCRTACA	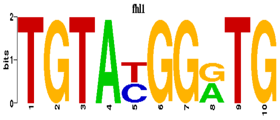
mbp1	2.9121e-02	CAYCCRTACA<br>-ACGCGT---	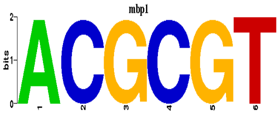
fkh2	4.8339e-02	CAYCCRTACA<br>-RYMAAYA--	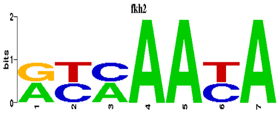
nrg1	1.4496e-01	TGTAYGGRTG-<br>----AGGGTCC	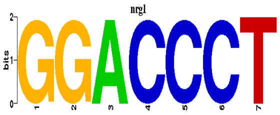
yap1	1.7772e-01	TGTAYGGRTG<br>MTTACGTAAK	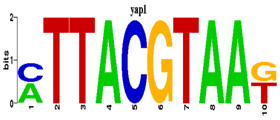

msn4

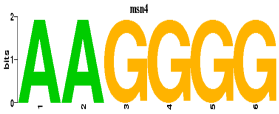 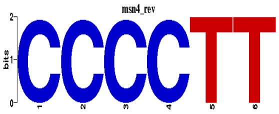

forward *reverse compliment*

Name	*E value*	*Alignment*	*Motif*
msn4	3.8873e-11	CCCCTT<br>CCCCTT	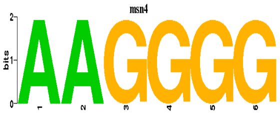
nrg1	3.3686e-03	AAGGGG--<br>-AGGGTCC	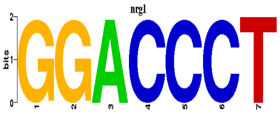

ndd1	6.7388e-02	CCCCTT----<br>CCNNWTYNGG	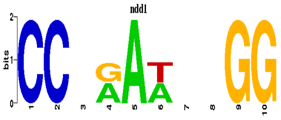
mcm1	1.5730e-01	---CCCCTT-------<br>TTACCNNWTTRGGWAW	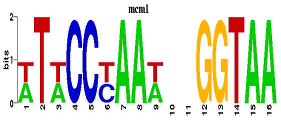
fhl1	2.9012e-01	--CCCCTT--<br>CAYCCRTACA	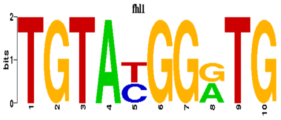

---

nrg1

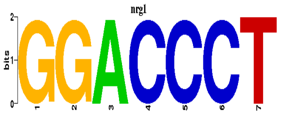

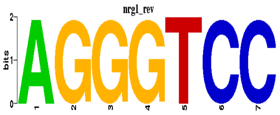

forward *reverse compliment*

Name	*E value*	*Alignment*	*Motif*
nrg1	1.0500e-12	AGGGTCC<br>AGGGTCC	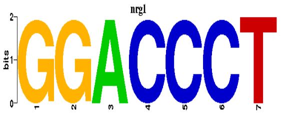
msn4	3.3686e-03	GGACCCT-<br>--CCCCTT	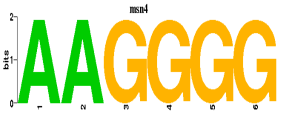
gcn4	1.3282e-02	AGGGTCC--<br>WGAGTCAYT	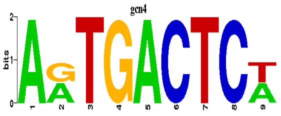
bas1	1.5524e-02	AGGGTCC<br>-GAGTCA	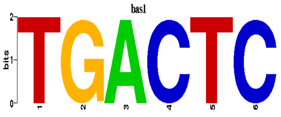
stb1	5.7498e-02	--AGGGTCC<br>TTSGCGTYY	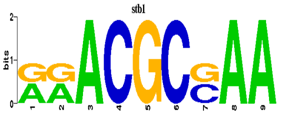

---

mbp1

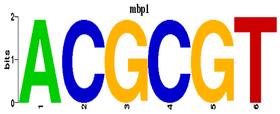 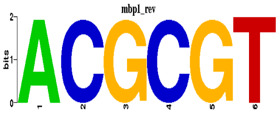

forward *reverse compliment*

Name	*E value*	*Alignment*	*Motif*
mbp1	3.8873e-11	ACGCGT<br>ACGCGT	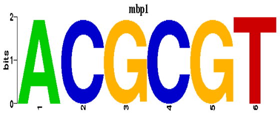
stb1	5.5510e-04	-ACGCGT--<br>TTSGCGTYY	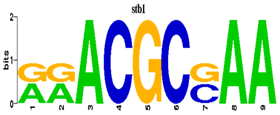
fhl1	2.9121e-02	-ACGCGT---<br>CAYCCRTACA	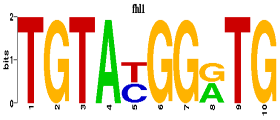
dal81	2.9402e-02	--ACGCGT-----<br>AAACGCAATTTTC	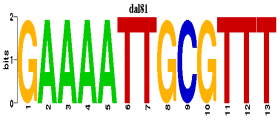
swi4	1.9854e-01	-ACGCGT-<br>TTTTCGTG	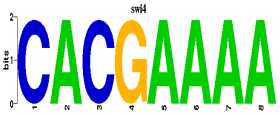

---

stb1

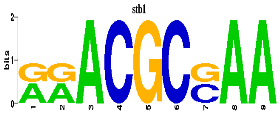 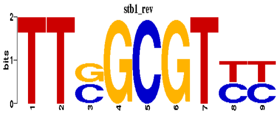

forward *reverse compliment*

Name	*E value*	*Alignment*	*Motif*
stb1	1.1102e-16	TTSGCGTYY<br>TTSGCGTYY	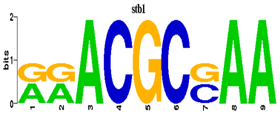
mbp1	5.5510e-04	RRACGCSAA<br>--ACGCGT-	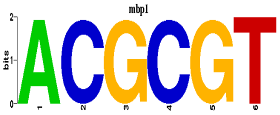

dal81	2.8258e-03	----TTSGCGTYY<br>GAAAATTGCGTTT	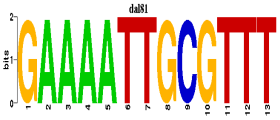
swi4	3.1053e-02	--TTSGCGTYY<br>TTTTCGTG---	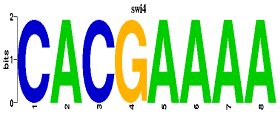
nrg1	5.7498e-02	TTSGCGTYY<br>--AGGGTCC	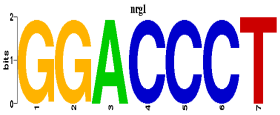

---

swi4

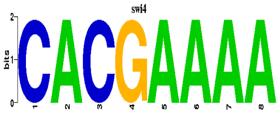

forward

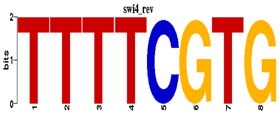

reverse compliment

Name	*E value*	*Alignment*	*Motif*
swi4	6.5503e-15	TTTTCGTG<br>TTTTCGTG	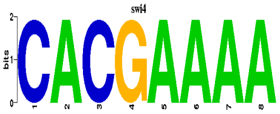
ste12	3.1086e-03	-TTTTCGTG<br>TGTTTCAT-	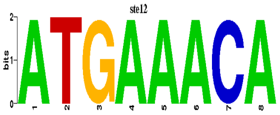
dal81	8.2593e-03	--------TTTTCGTG<br>AAACGCAATTTTC---	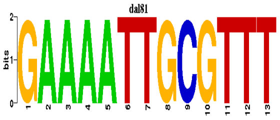
yap1	1.0831e-02	TTTTCGTG--<br>MTTACGTAAK	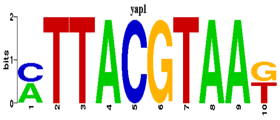
dig1	2.0287e-02	-TTTTCGTG<br>TGTTTCA--	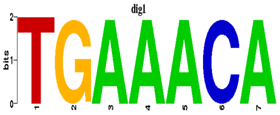

---

yap1

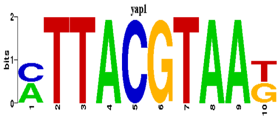

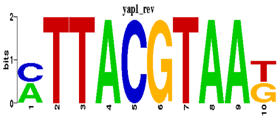

Name	E value	Alignment (*forward* / *reverse compliment*)	Motif
yap1	0.0000e+00	MTTACGTAAK<br>MTTACGTAAK	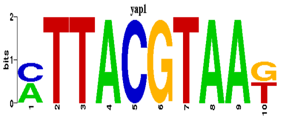
swi4	1.0831e-02	MTTACGTAAK<br>TTTTCGTG--	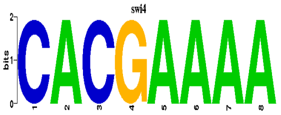
yap6	1.3855e-02	MTTACGTAAK<br>--TTAGTAA-	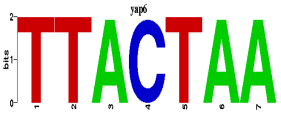
dal81	2.2753e-02	MTTACGTAAK----<br>-AAACGCAATTTTC	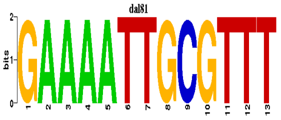
mcm1	2.7291e-02	-------MTTACGTAAK<br>TTACCNNWTTRGGWAW-	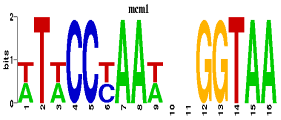

---

gat3

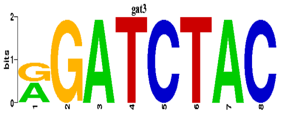

forward

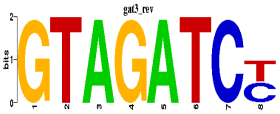

reverse compliment

Name	E value	Alignment	Motif
gat3	6.5503e-15	GTAGATCY<br>GTAGATCY	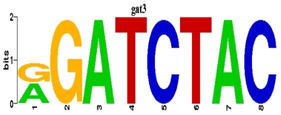
nrg1	1.8861e-01	-RGATCTAC<br>AGGGTCC--	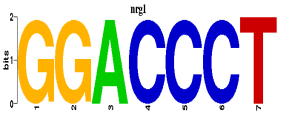
yap6	3.0607e-01	---GTAGATCY<br>TTAGTAA----	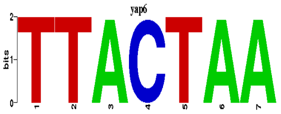

fkh2	3.1912e-01	RGATCTAC<br>-TRTTKRY	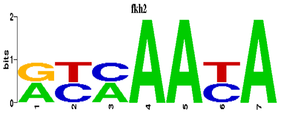
fhl1	4.0231e-01	-----GTAGATCY<br>CAYCCRTACA---	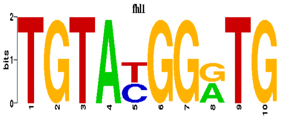

hsf1

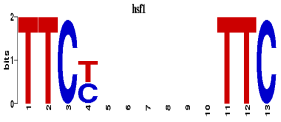

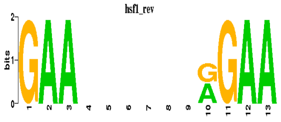

forward *reverse compliment*

Name	*E value*	*Alignment*	*Motif*
hsf1	3.3200e-04	GAANNNNNNRGAA<br>GAANNNNNNRGAA	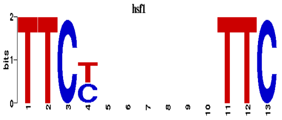
dig1	5.5941e-01	---TTCYNNNNNNTTC<br>TGTTTCA---------	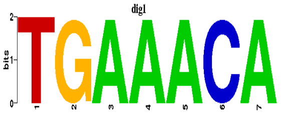
gat3	6.7947e-01	----TTCYNNNNNNTTC<br>GTAGATCY---------	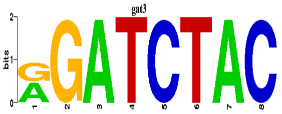
ste12	6.7947e-01	---TTCYNNNNNNTTC<br>TGTTTCAT--------	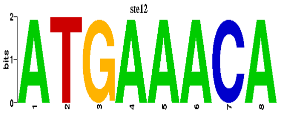
swi4	6.7947e-01	--TTCYNNNNNNTTC<br>TTTTCGTG-------	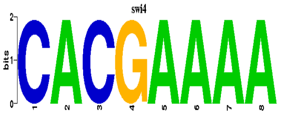

gal4

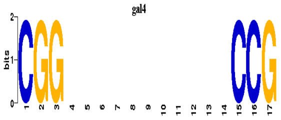

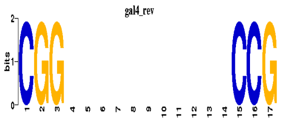

forward *reverse compliment*

Name	*E value*	*Alignment*	*Motif*
gal4	1.5438e-01	CGGNNNNNNNNNNNCCG<br>CGGNNNNNNNNNNNCCG	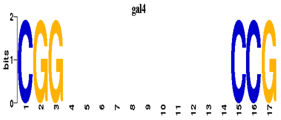
leu3	8.9750e-01	-------CGGNNNNNNNNNNNCCG<br>CCGNNNNCGG--------------	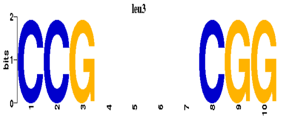
ace2	9.5579e-01	CGGNNNNNNNNNNNCCG--<br>-------------ACCAGC	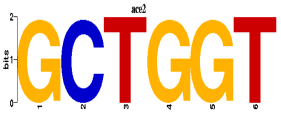
mbp1	9.5579e-01	-CGGNNNNNNNNNNNCCG<br>ACGCGT------------	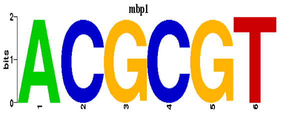
msn4	9.5579e-01	CGGNNNNNNNNNNNCCG---<br>--------------CCCCTT	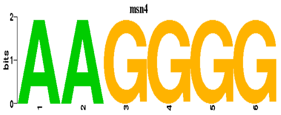

---

leu3

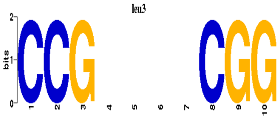
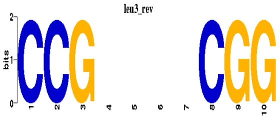

forward *reverse compliment*

Name	*E value*	*Alignment*	*Motif*
leu3	2.0663e-04	CCGNNNNCGG<br>CCGNNNNCGG	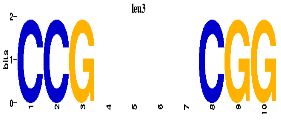
ndd1	2.0159e-01	CCGNNNNCGG<br>CCNNWTYNGG	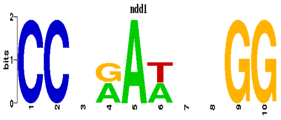
mcm1	7.3812e-01	---CCGNNNNCGG---<br>TTACCNNWTTRGGWAW	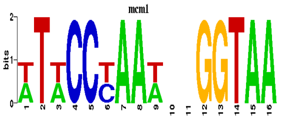
ace2	8.5980e-01	-CCGNNNNCGG<br>ACCAGC-----	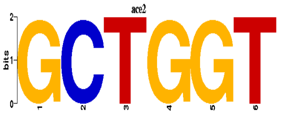

mbp1	8.5980e-01	CCGNNNNCGG<br>ACGCGT----	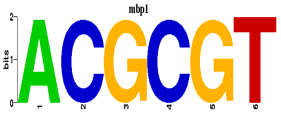

Sequence logo generation powered by weblogo
STAMP is written by Shaun Mahony
