# Supplementary material for: Computational study of associations between histone modification and protein-DNA binding in yeast genome by integrating diverse information
Source: BMC Genomics. 2011 Apr 1;12:172. doi: 10.1186/1471-2164-12-172 (PMC3082246; doi:10.1186/1471-2164-12-172)
Supplement: Additional file 4 — AddFile4_18clusters_orf_functional.zip ZIP files. Protein clustering for functional binding target. Here contains results (18clusters_orf_function.html) of 18 clusters for functional binding sites. [file 1471-2164-12-172-S4.ZIP › supFile1_18clusters_orf_function.html]

# **Result of Clustering**

***## Picture of C1***
  

## ***T-values of C1***

  
  
  
  
***## Picture of C2***
  

## ***T-values of C2***

  
  
  
  
***## Picture of C3***
  

## ***T-values of C3***

  
  
  
  
***## Picture of C4***
  

## ***T-values of C4***

  
  
  
  
***## Picture of C5***
  

## ***T-values of C5***

  
  
  
  
***## Picture of C6***
  

## ***T-values of C6***

  
  
  
  
***## Picture of C7***
  

## ***T-values of C7***

  
  
  
  
***## Picture of C8***
  

## ***T-values of C8***

  
  
  
  
***## Picture of C9***
  

## ***T-values of C9***

  
  
  
  
***## Picture of C10***
  

## ***T-values of C10***

  
  
  
  
***## Picture of C11***
  

## ***T-values of C11***

  
  
  
  
***## Picture of C12***
  

## ***T-values of C12***

  
  
  
  
***## Picture of C13***
  

## ***T-values of C13***

  
  
  
  
***## Picture of C14***
  

## ***T-values of C14***

  
  
  
  
***## Picture of C15***
  

## ***T-values of C15***

  
  
  
  
***## Picture of C16***
  

## ***T-values of C16***

  
  
  
  
***## Picture of C17***
  

## ***T-values of C17***

  
  
  
  
***## Picture of C18***
  

## ***T-values of C18***
