# Supplementary figures and images for: Computational study of associations between histone modification and protein-DNA binding in yeast genome by integrating diverse information
Source: BMC Genomics. 2011 Apr 1;12:172. doi: 10.1186/1471-2164-12-172 (PMC3082246; doi:10.1186/1471-2164-12-172)

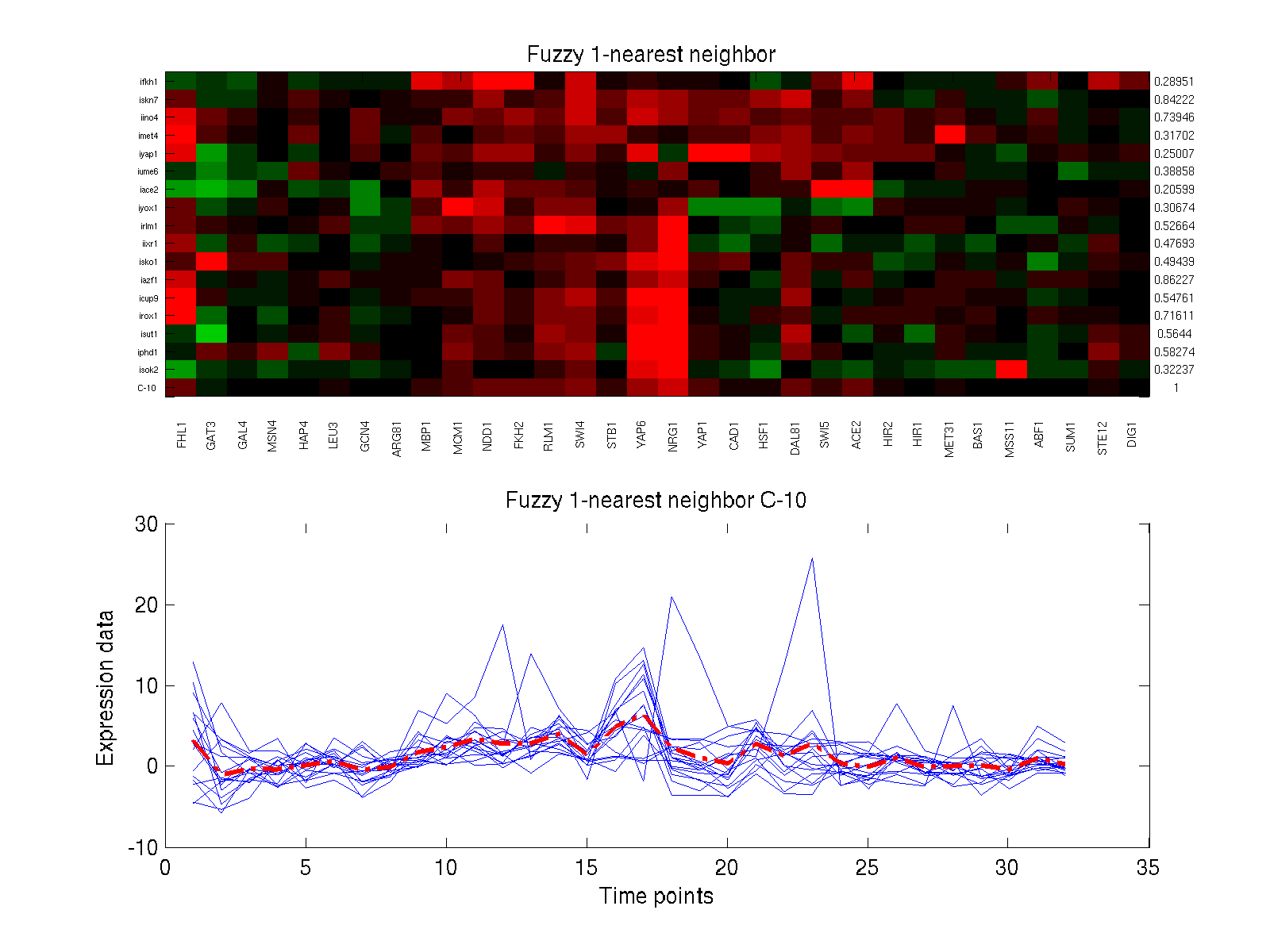

Supplement: Additional file 4 — AddFile4_18clusters_orf_functional.zip ZIP files. Protein clustering for functional binding target. Here contains results (18clusters_orf_function.html) of 18 clusters for functional binding sites. [file 1471-2164-12-172-S4.ZIP › C-10.png]

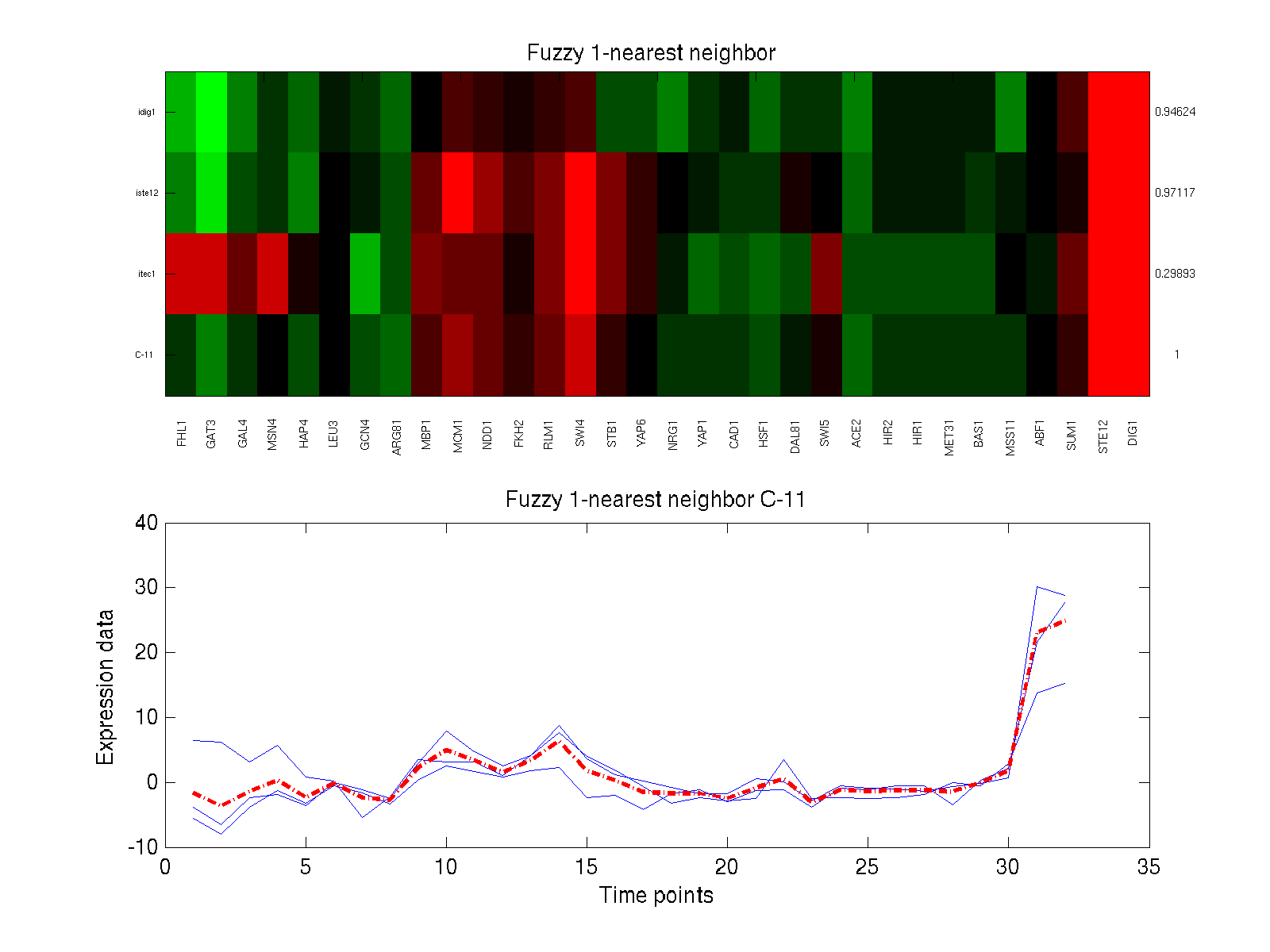

Supplement: Additional file 4 — AddFile4_18clusters_orf_functional.zip ZIP files. Protein clustering for functional binding target. Here contains results (18clusters_orf_function.html) of 18 clusters for functional binding sites. [file 1471-2164-12-172-S4.ZIP › C-11.png]

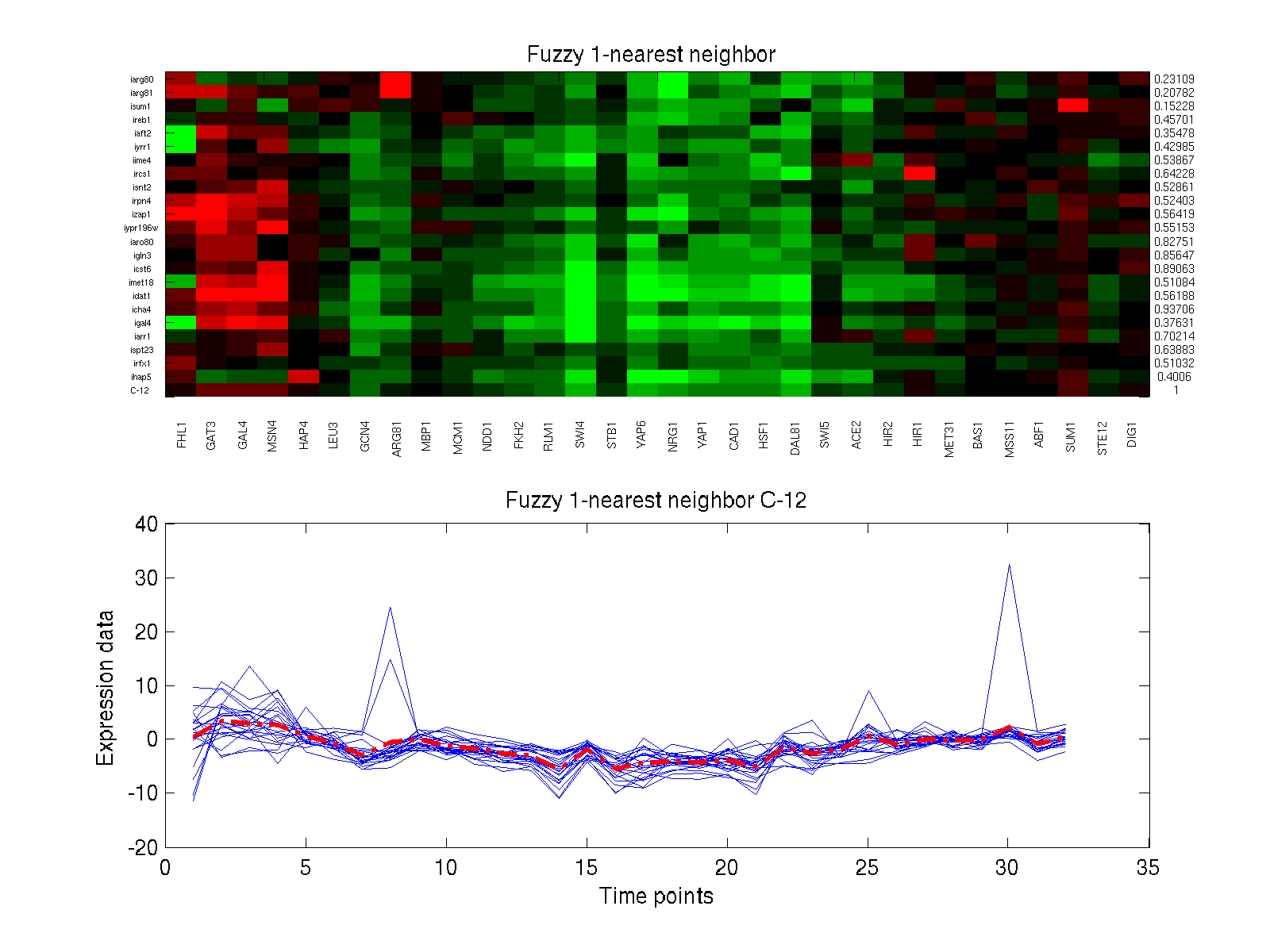

Supplement: Additional file 4 — AddFile4_18clusters_orf_functional.zip ZIP files. Protein clustering for functional binding target. Here contains results (18clusters_orf_function.html) of 18 clusters for functional binding sites. [file 1471-2164-12-172-S4.ZIP › C-12.png]

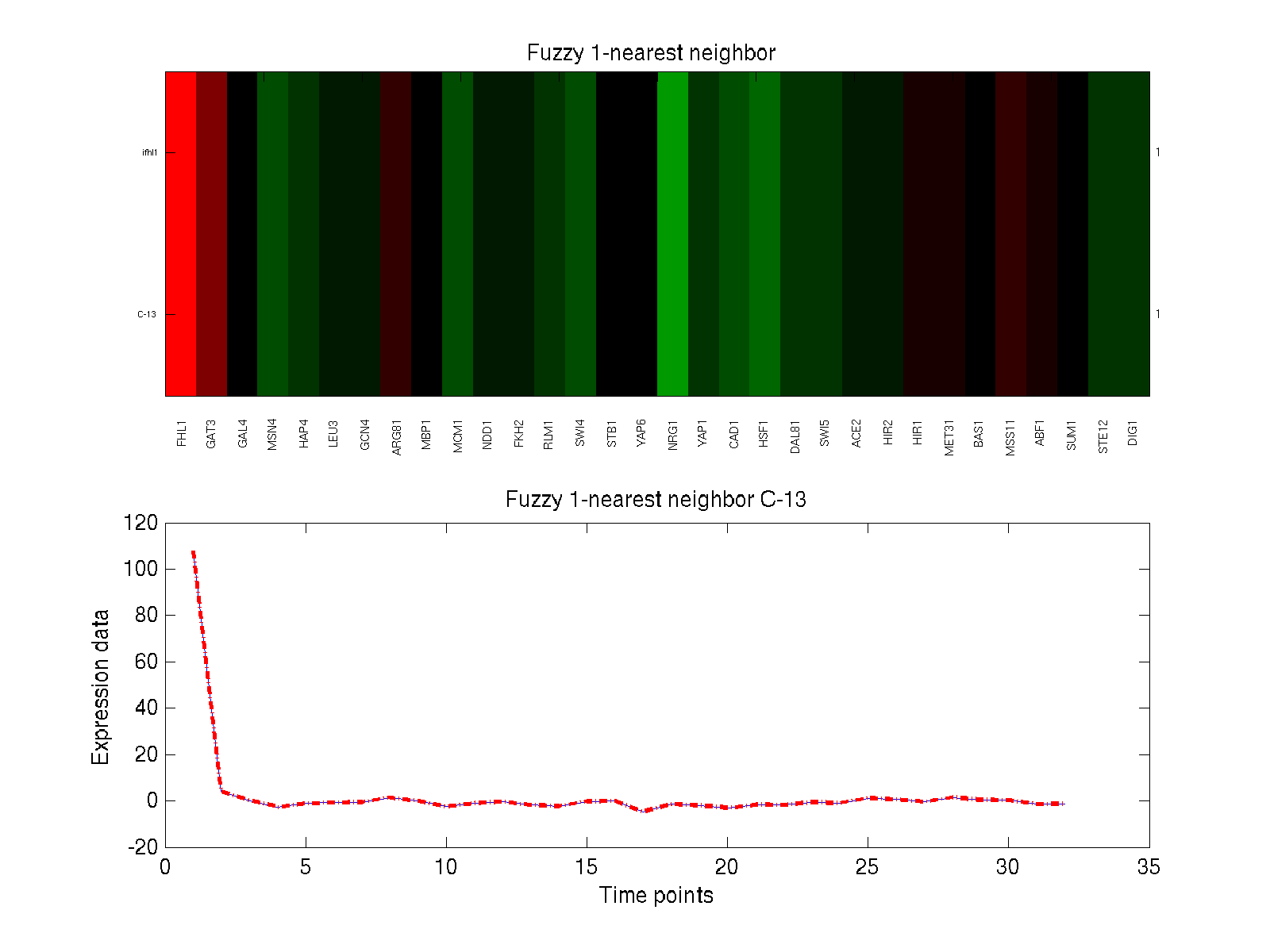

Supplement: Additional file 4 — AddFile4_18clusters_orf_functional.zip ZIP files. Protein clustering for functional binding target. Here contains results (18clusters_orf_function.html) of 18 clusters for functional binding sites. [file 1471-2164-12-172-S4.ZIP › C-13.png]

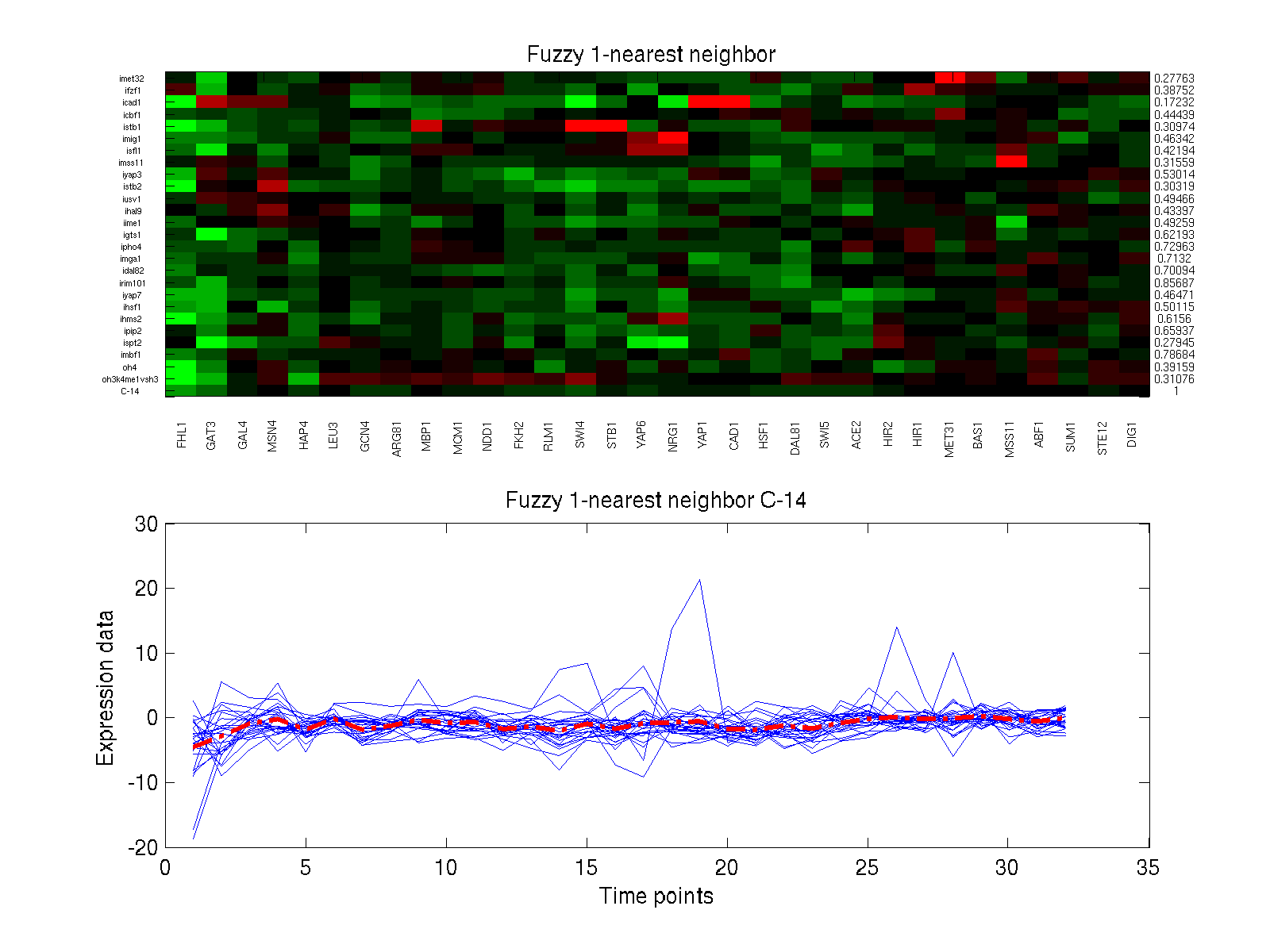

Supplement: Additional file 4 — AddFile4_18clusters_orf_functional.zip ZIP files. Protein clustering for functional binding target. Here contains results (18clusters_orf_function.html) of 18 clusters for functional binding sites. [file 1471-2164-12-172-S4.ZIP › C-14.png]

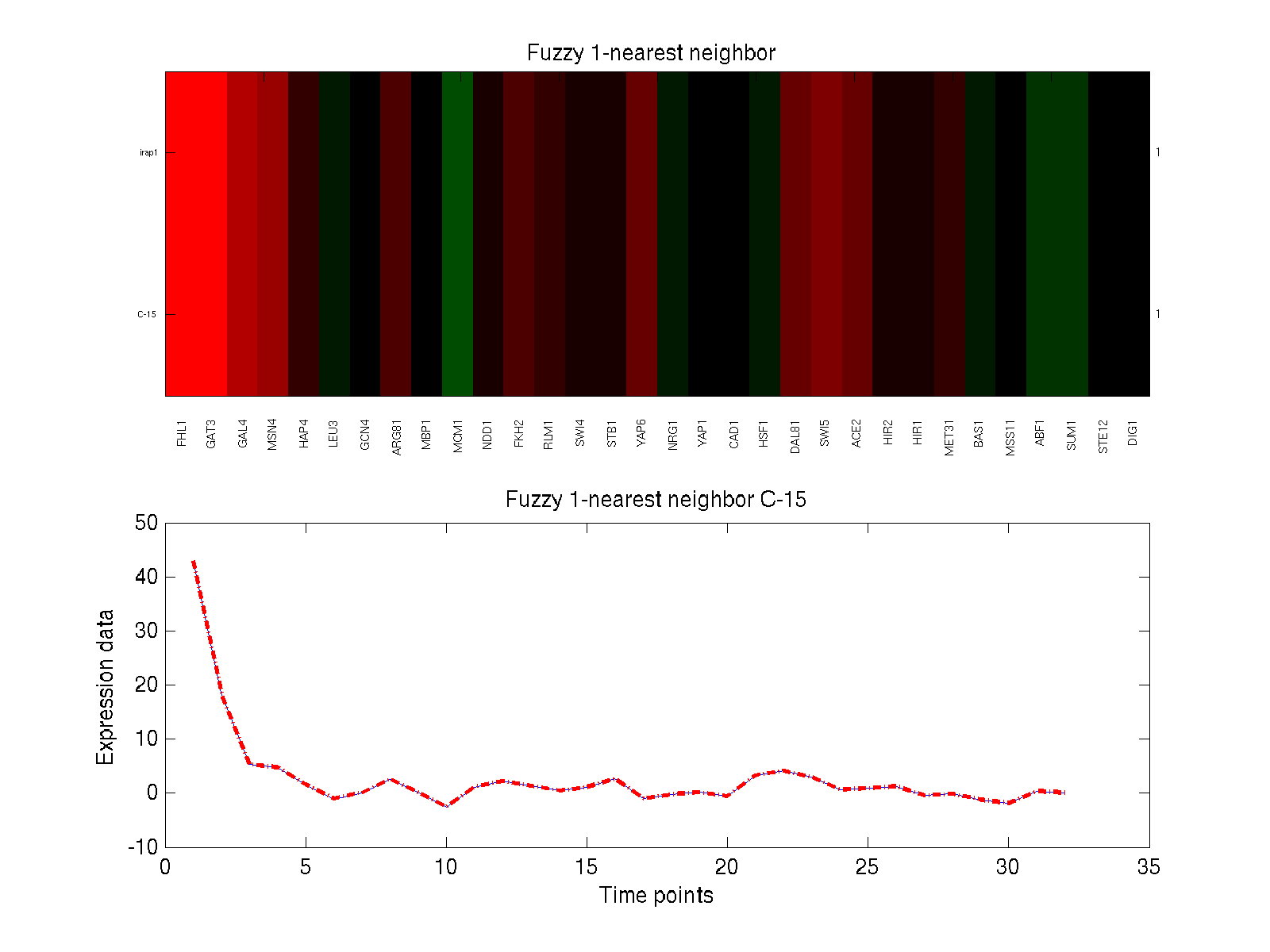

Supplement: Additional file 4 — AddFile4_18clusters_orf_functional.zip ZIP files. Protein clustering for functional binding target. Here contains results (18clusters_orf_function.html) of 18 clusters for functional binding sites. [file 1471-2164-12-172-S4.ZIP › C-15.png]

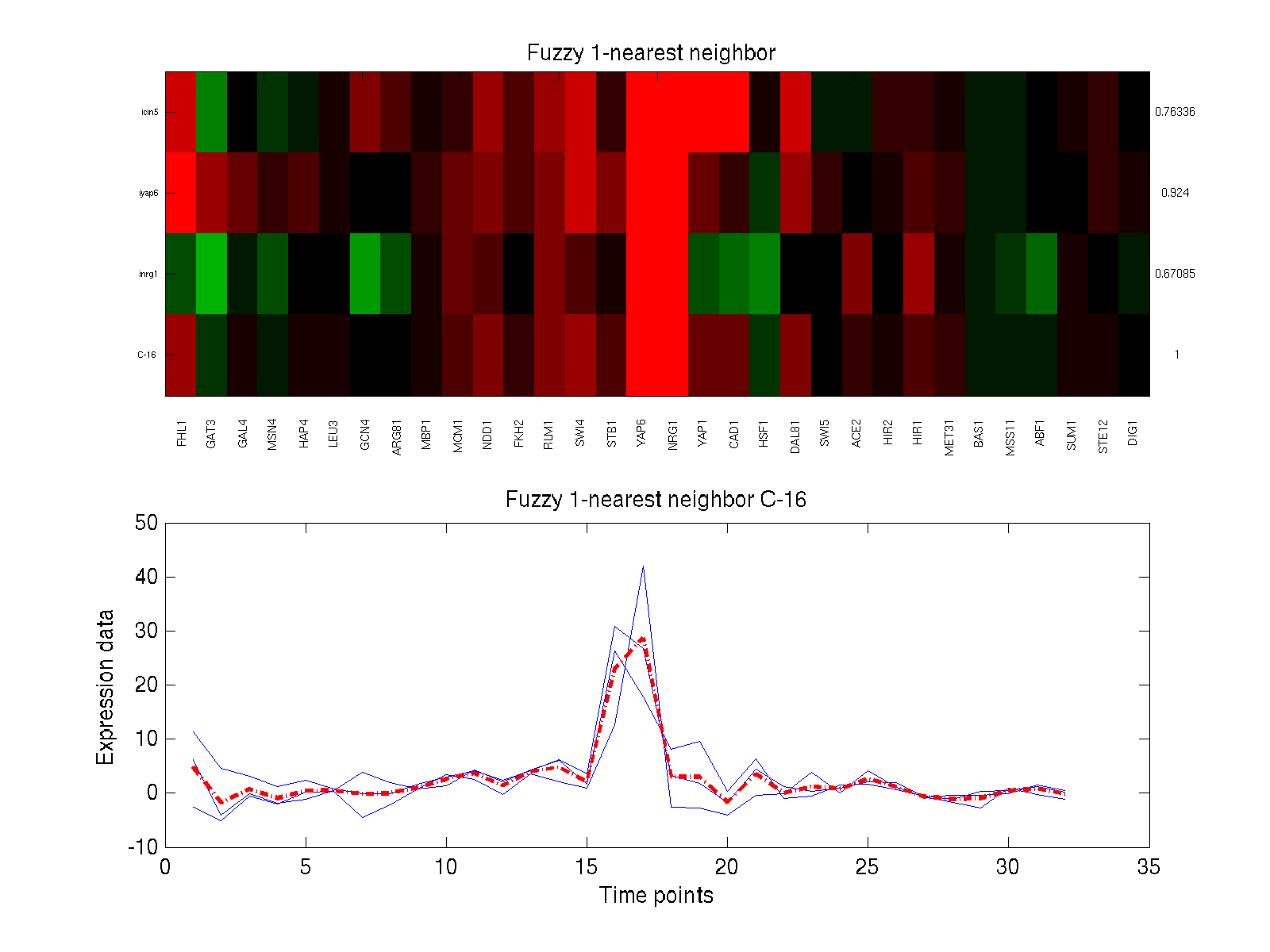

Supplement: Additional file 4 — AddFile4_18clusters_orf_functional.zip ZIP files. Protein clustering for functional binding target. Here contains results (18clusters_orf_function.html) of 18 clusters for functional binding sites. [file 1471-2164-12-172-S4.ZIP › C-16.png]

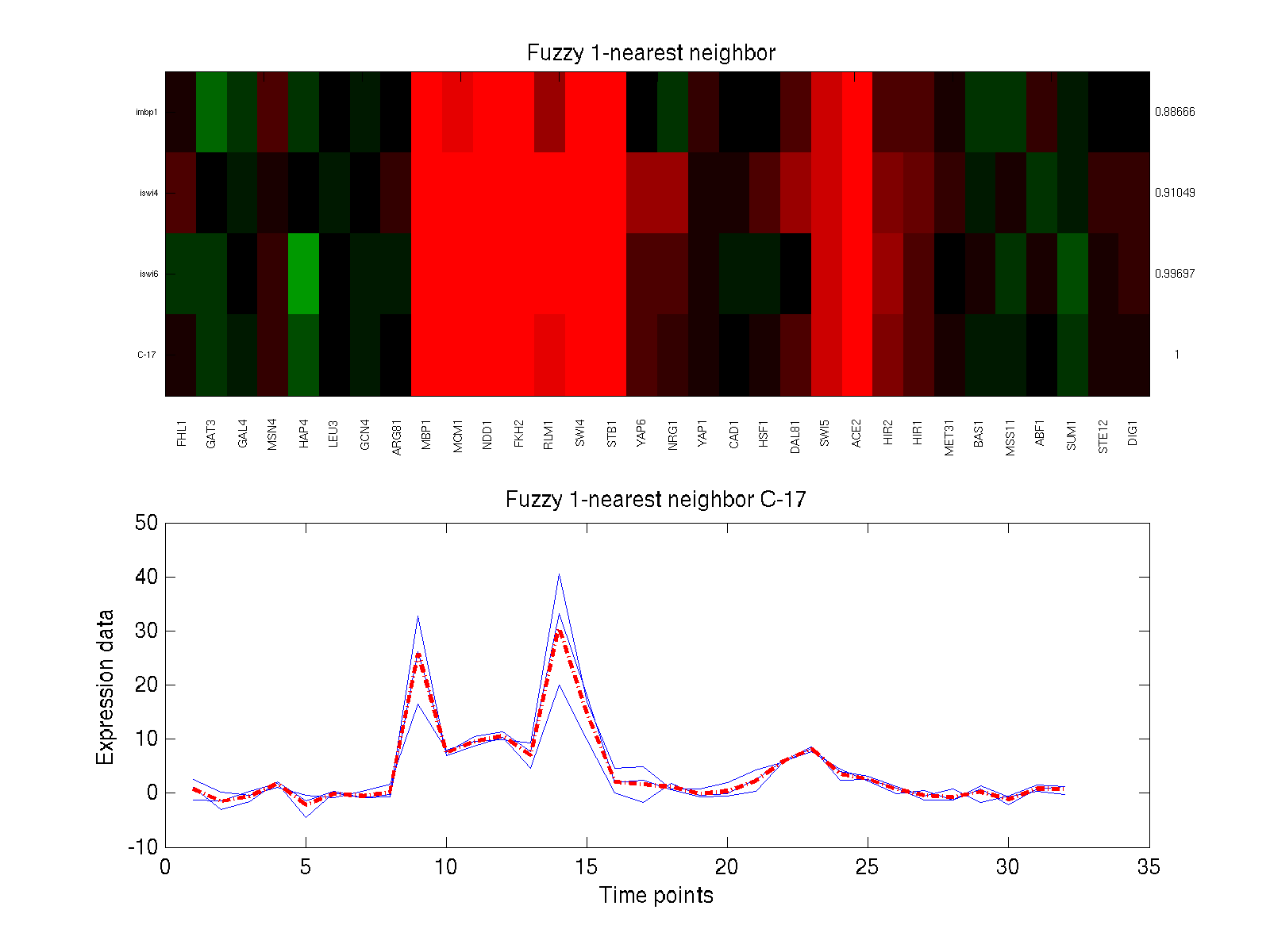

Supplement: Additional file 4 — AddFile4_18clusters_orf_functional.zip ZIP files. Protein clustering for functional binding target. Here contains results (18clusters_orf_function.html) of 18 clusters for functional binding sites. [file 1471-2164-12-172-S4.ZIP › C-17.png]

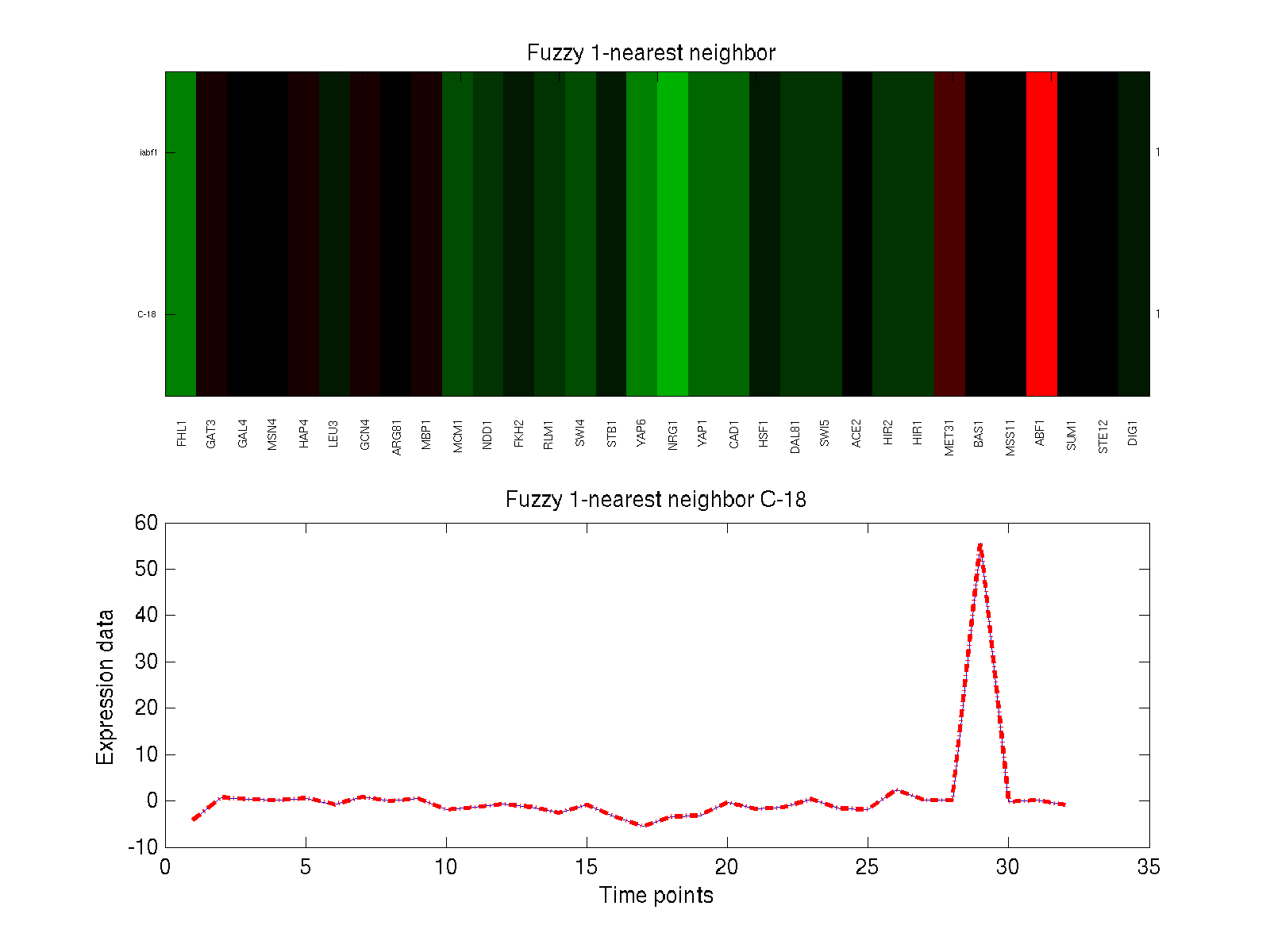

Supplement: Additional file 4 — AddFile4_18clusters_orf_functional.zip ZIP files. Protein clustering for functional binding target. Here contains results (18clusters_orf_function.html) of 18 clusters for functional binding sites. [file 1471-2164-12-172-S4.ZIP › C-18.png]

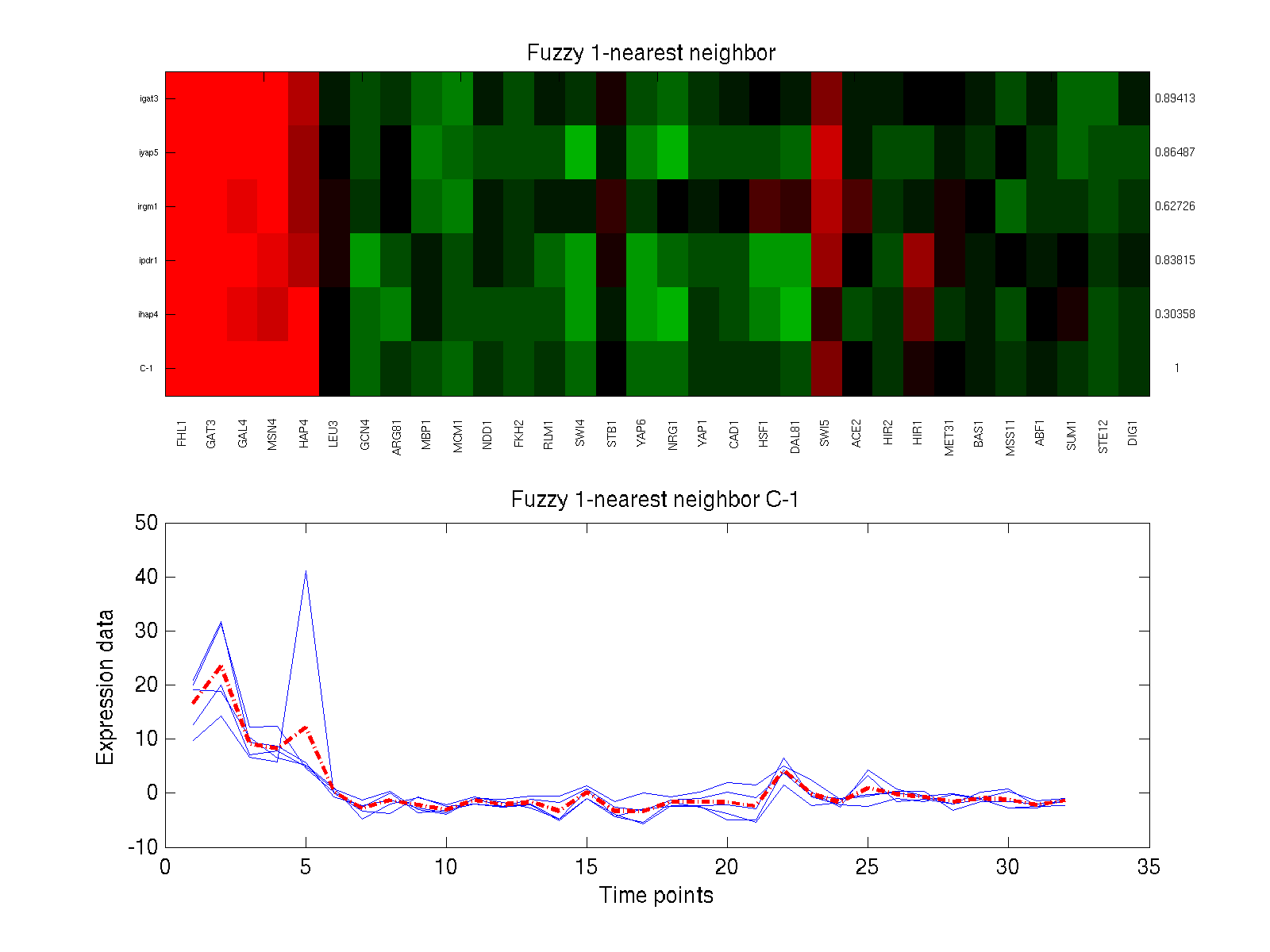

Supplement: Additional file 4 — AddFile4_18clusters_orf_functional.zip ZIP files. Protein clustering for functional binding target. Here contains results (18clusters_orf_function.html) of 18 clusters for functional binding sites. [file 1471-2164-12-172-S4.ZIP › C-1.png]

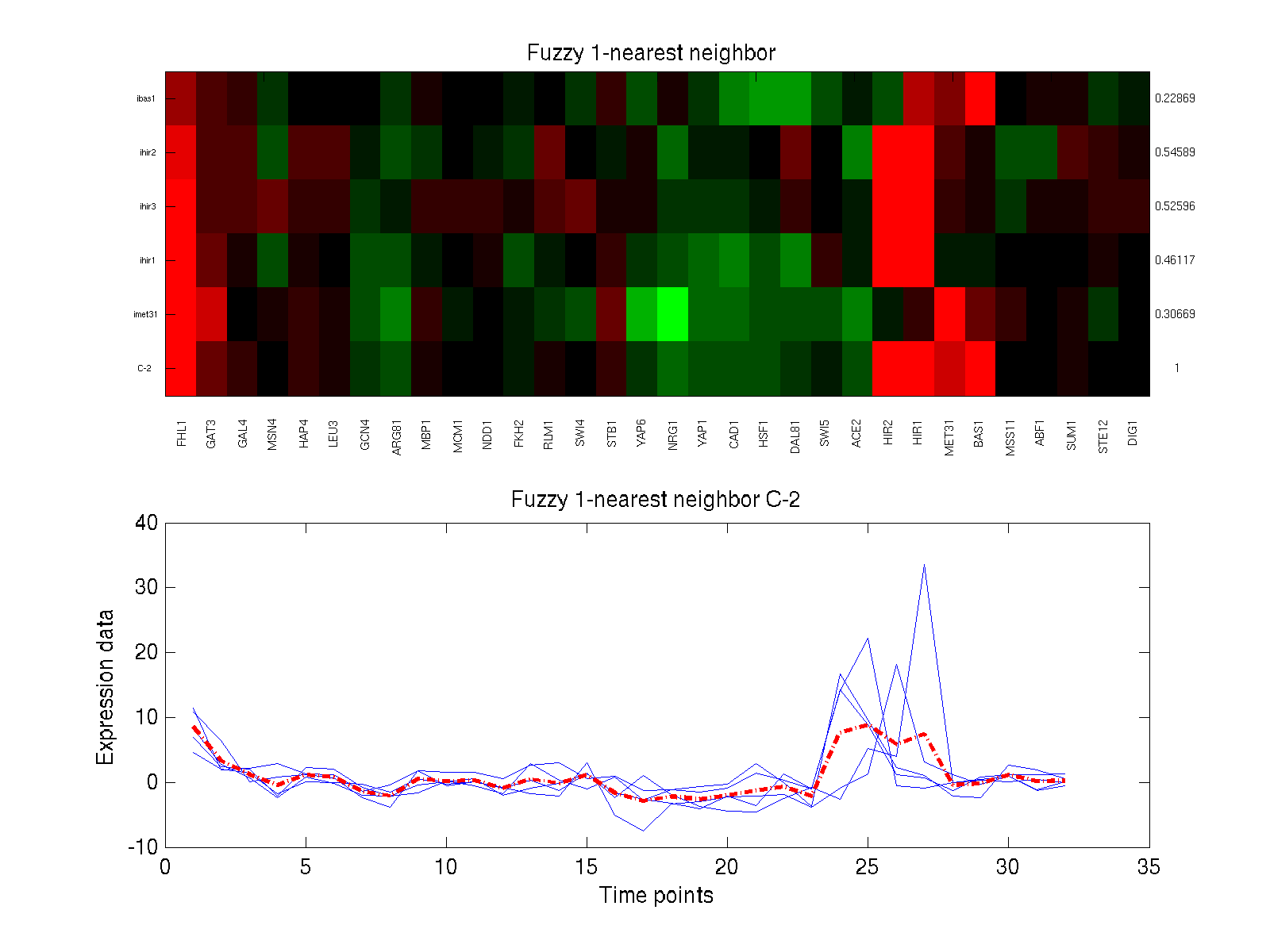

Supplement: Additional file 4 — AddFile4_18clusters_orf_functional.zip ZIP files. Protein clustering for functional binding target. Here contains results (18clusters_orf_function.html) of 18 clusters for functional binding sites. [file 1471-2164-12-172-S4.ZIP › C-2.png]

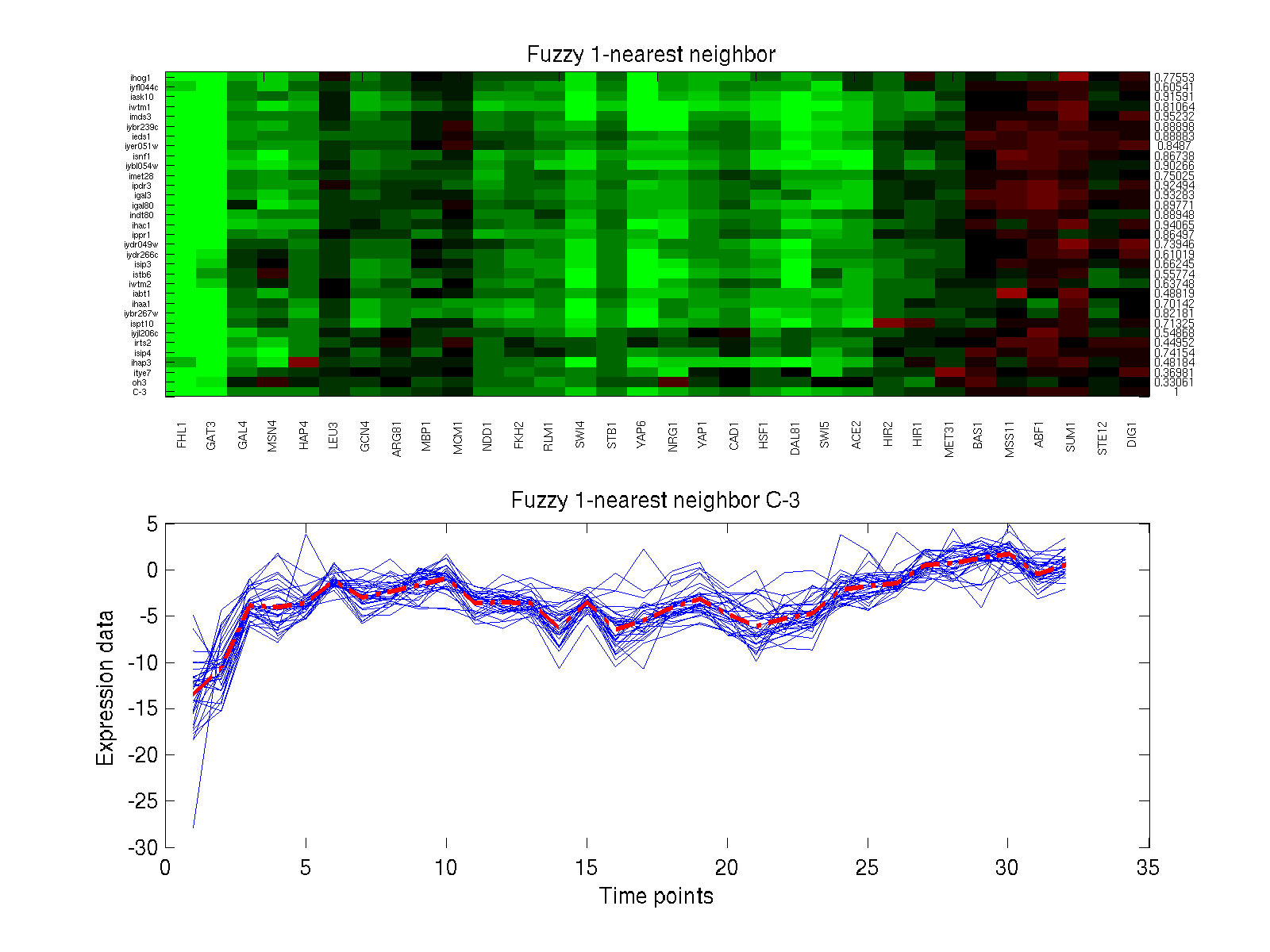

Supplement: Additional file 4 — AddFile4_18clusters_orf_functional.zip ZIP files. Protein clustering for functional binding target. Here contains results (18clusters_orf_function.html) of 18 clusters for functional binding sites. [file 1471-2164-12-172-S4.ZIP › C-3.png]

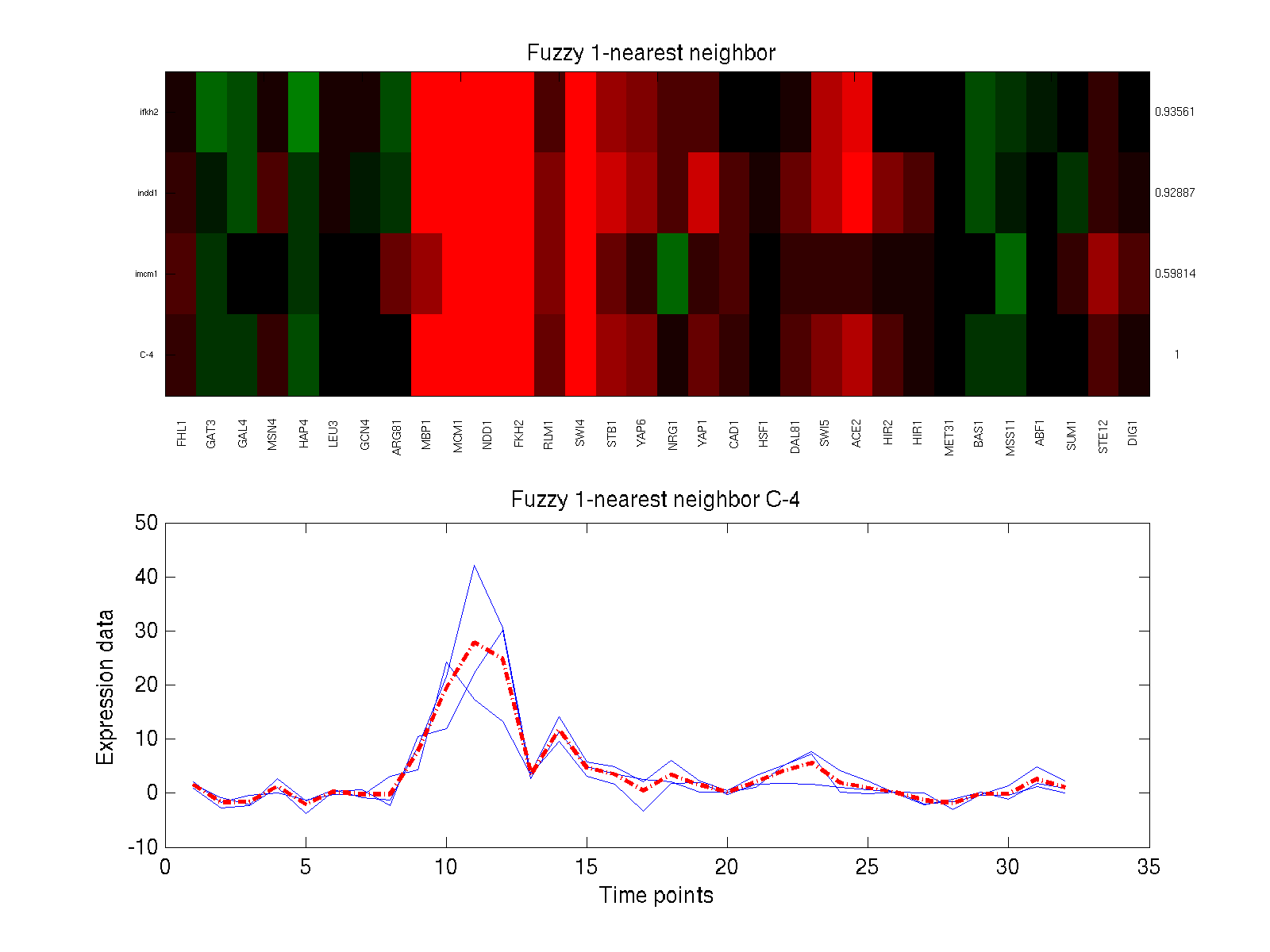

Supplement: Additional file 4 — AddFile4_18clusters_orf_functional.zip ZIP files. Protein clustering for functional binding target. Here contains results (18clusters_orf_function.html) of 18 clusters for functional binding sites. [file 1471-2164-12-172-S4.ZIP › C-4.png]

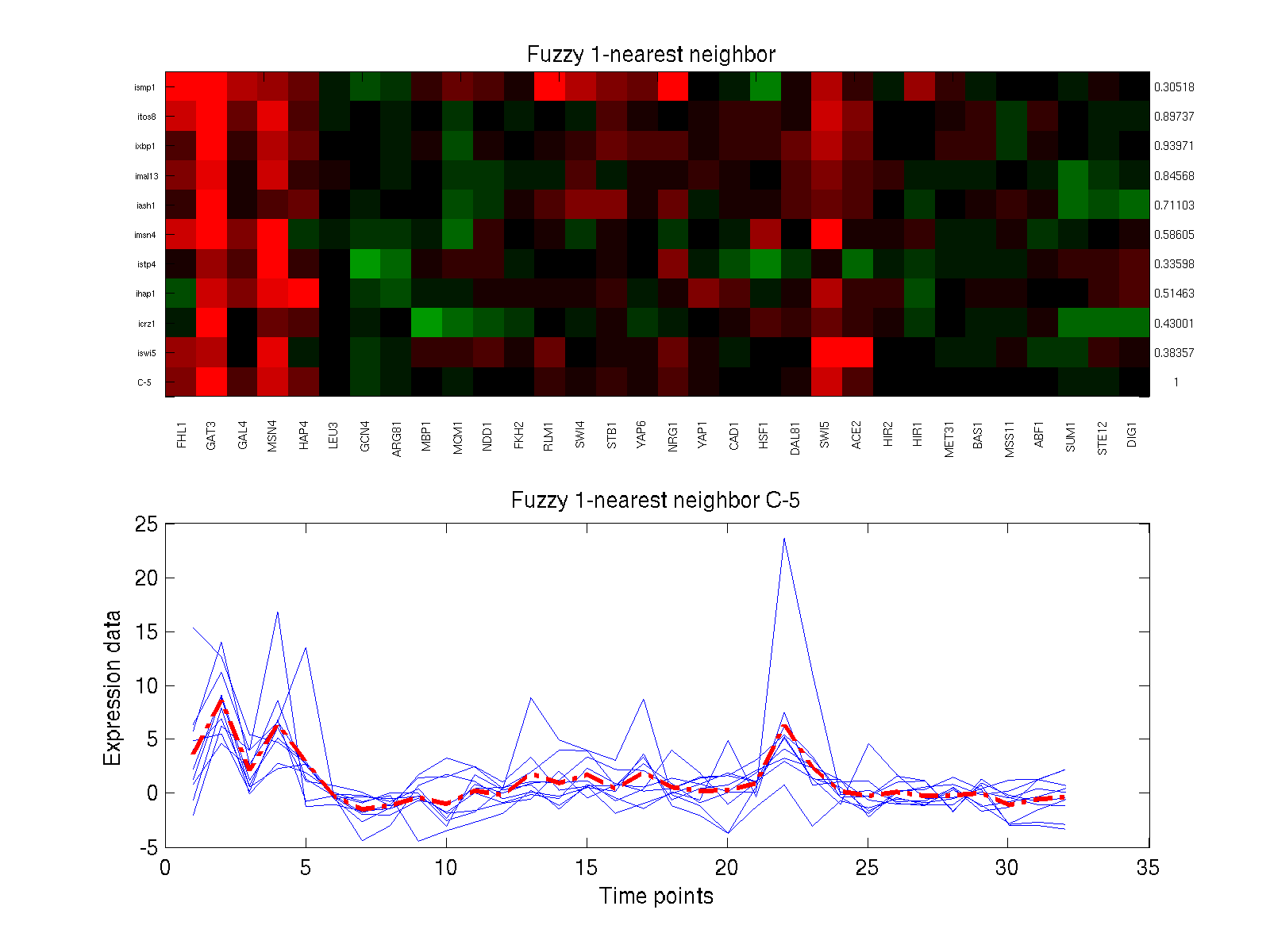

Supplement: Additional file 4 — AddFile4_18clusters_orf_functional.zip ZIP files. Protein clustering for functional binding target. Here contains results (18clusters_orf_function.html) of 18 clusters for functional binding sites. [file 1471-2164-12-172-S4.ZIP › C-5.png]

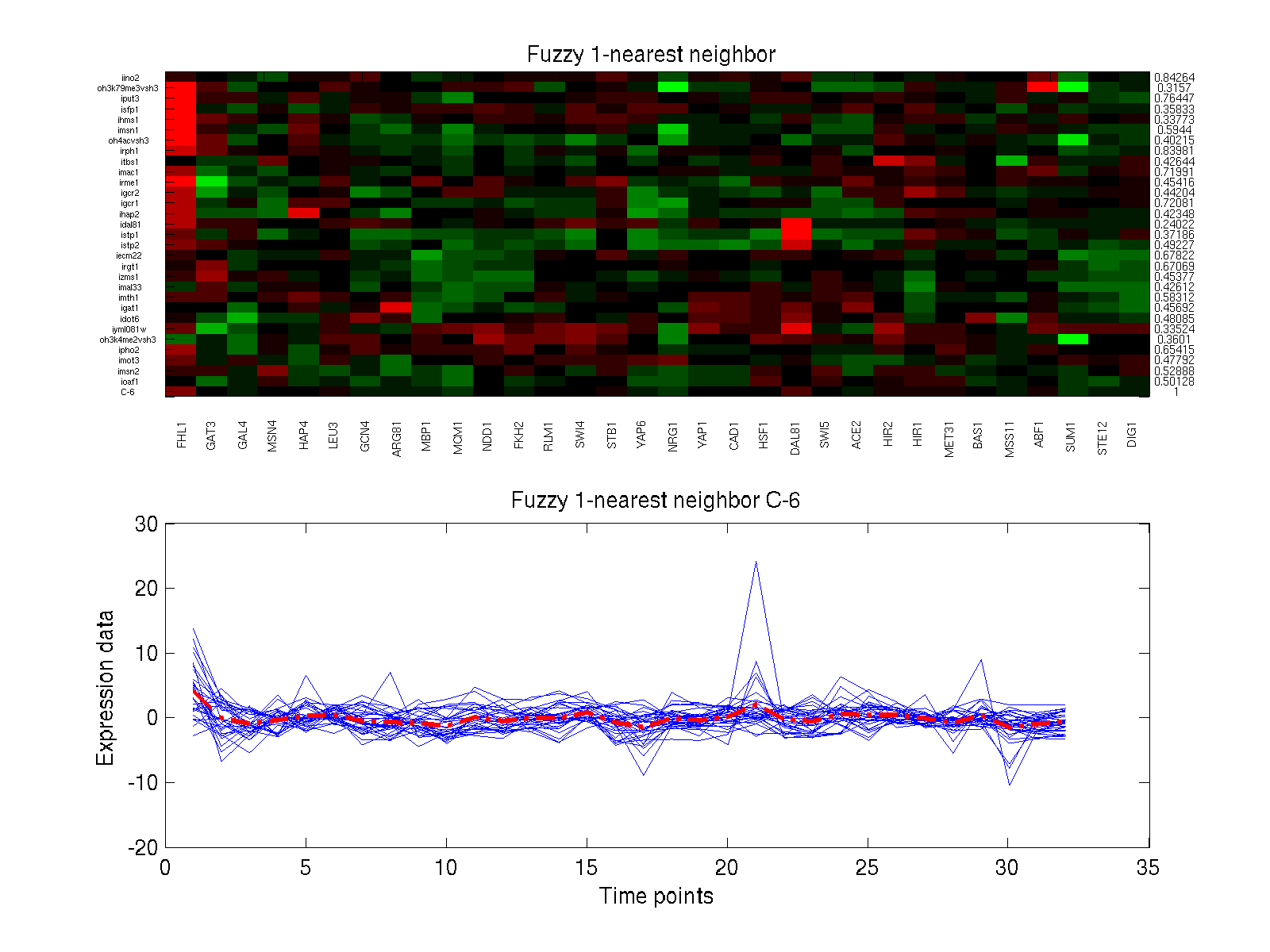

Supplement: Additional file 4 — AddFile4_18clusters_orf_functional.zip ZIP files. Protein clustering for functional binding target. Here contains results (18clusters_orf_function.html) of 18 clusters for functional binding sites. [file 1471-2164-12-172-S4.ZIP › C-6.png]

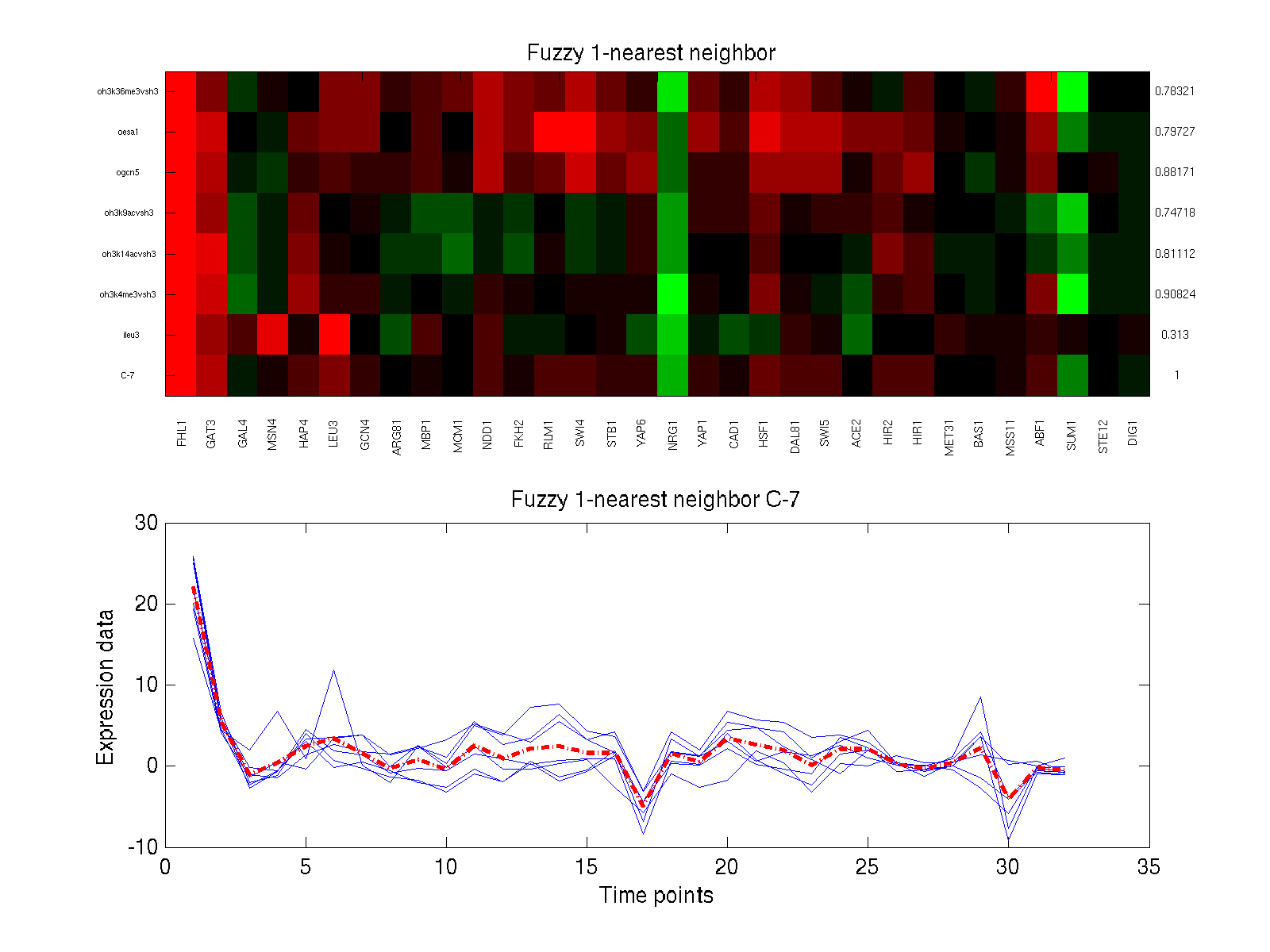

Supplement: Additional file 4 — AddFile4_18clusters_orf_functional.zip ZIP files. Protein clustering for functional binding target. Here contains results (18clusters_orf_function.html) of 18 clusters for functional binding sites. [file 1471-2164-12-172-S4.ZIP › C-7.png]

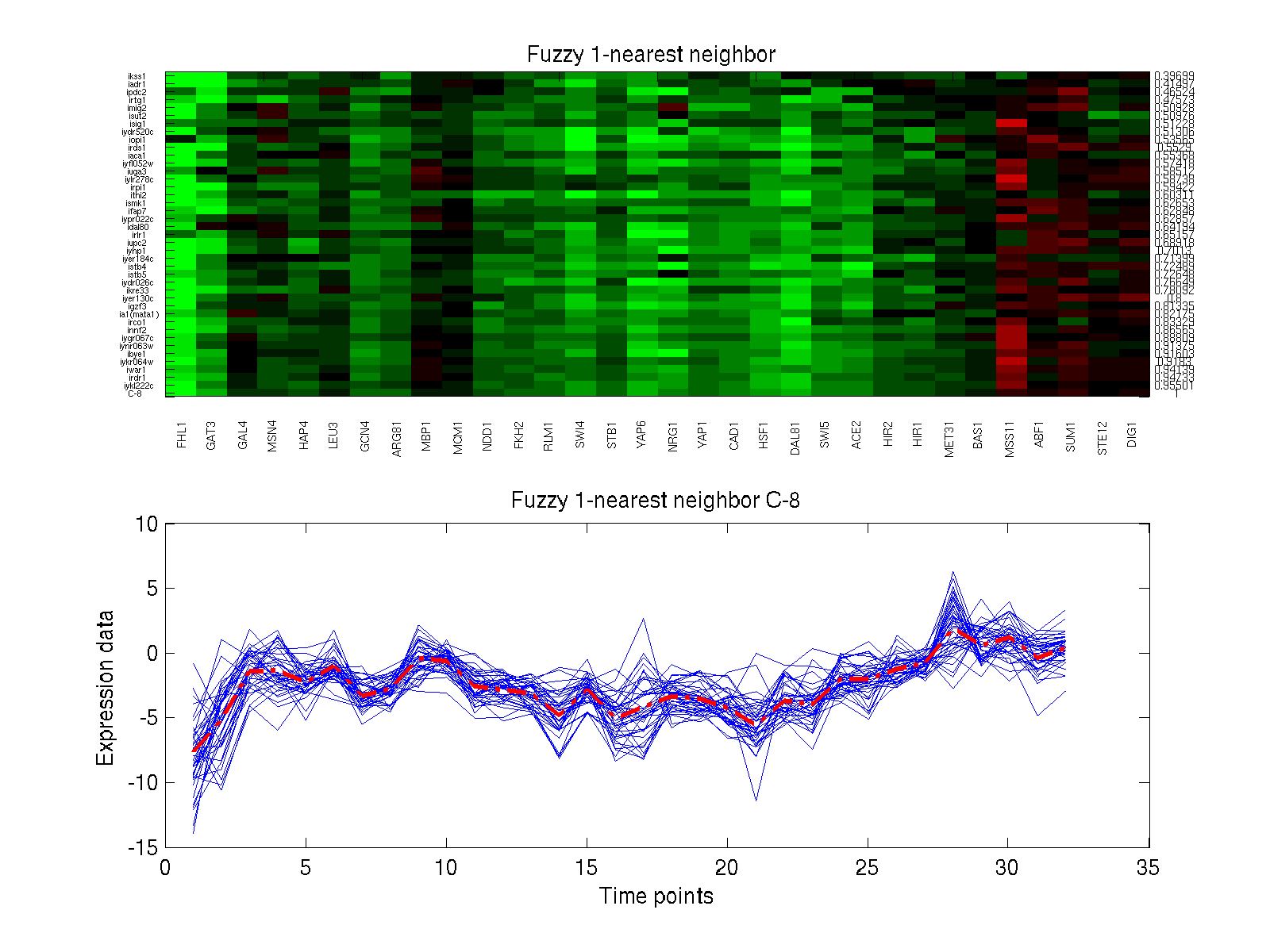

Supplement: Additional file 4 — AddFile4_18clusters_orf_functional.zip ZIP files. Protein clustering for functional binding target. Here contains results (18clusters_orf_function.html) of 18 clusters for functional binding sites. [file 1471-2164-12-172-S4.ZIP › C-8.png]

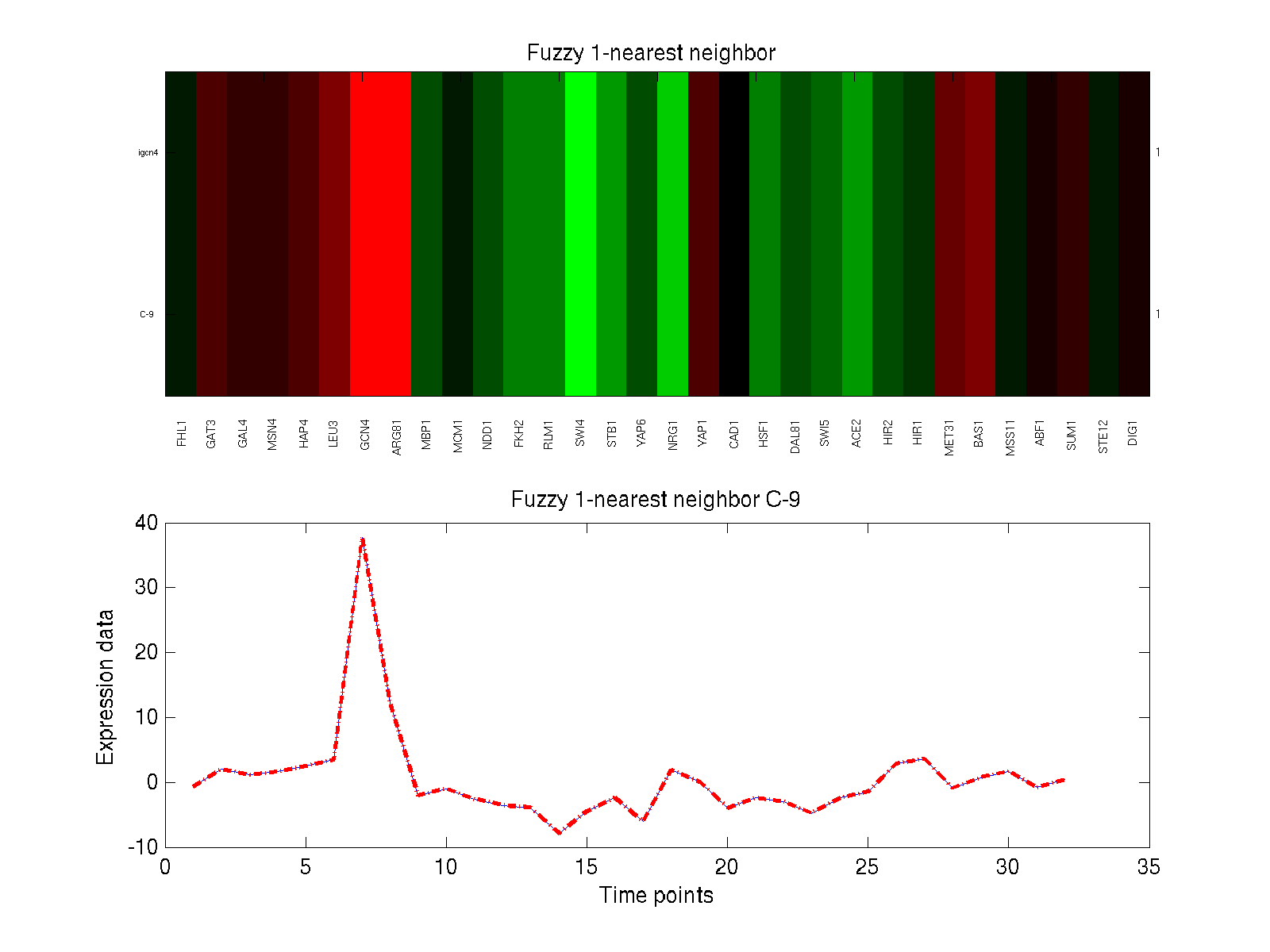

Supplement: Additional file 4 — AddFile4_18clusters_orf_functional.zip ZIP files. Protein clustering for functional binding target. Here contains results (18clusters_orf_function.html) of 18 clusters for functional binding sites. [file 1471-2164-12-172-S4.ZIP › C-9.png]

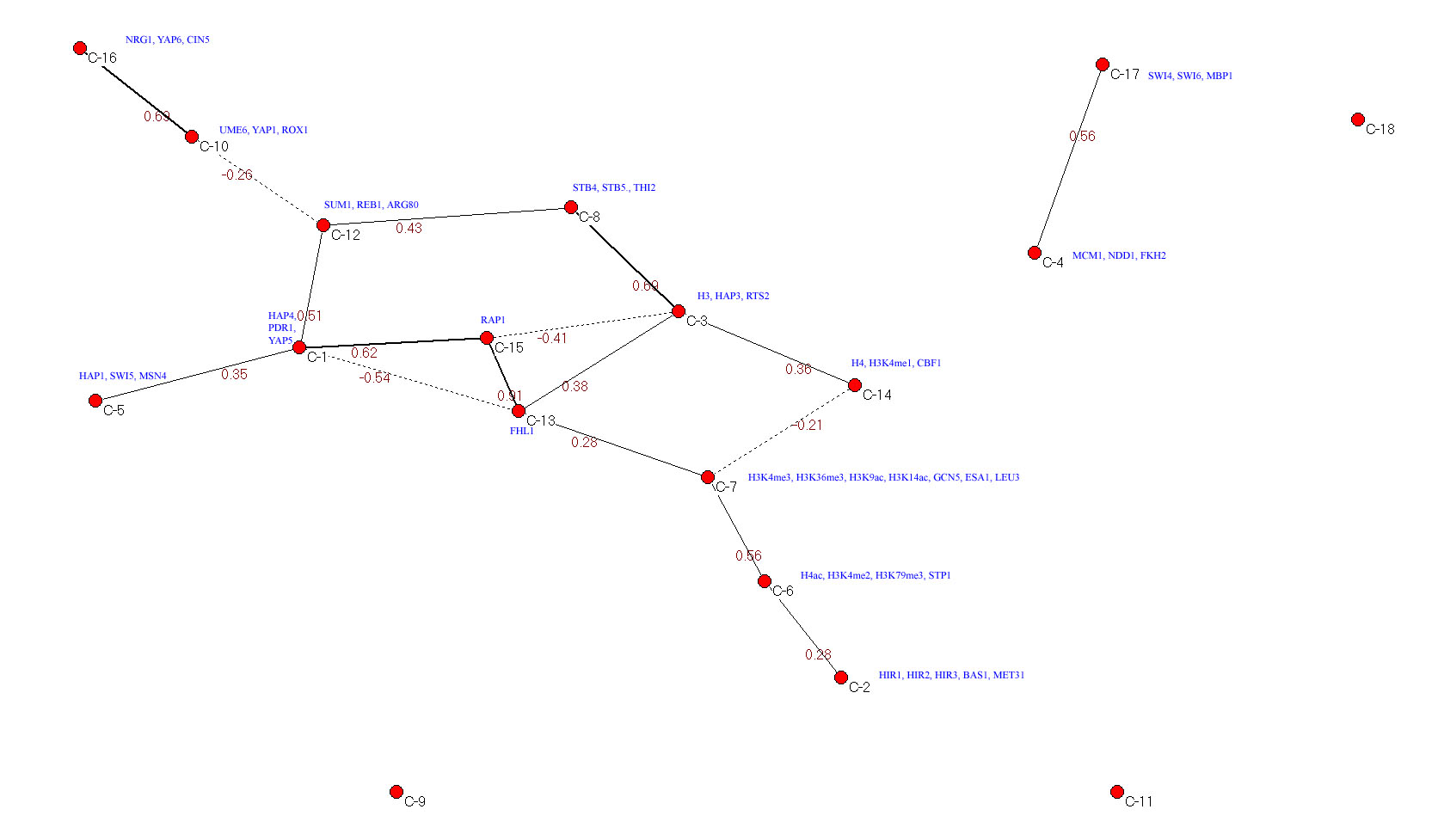

Supplement: Additional file 4 — AddFile4_18clusters_orf_functional.zip ZIP files. Protein clustering for functional binding target. Here contains results (18clusters_orf_function.html) of 18 clusters for functional binding sites. [file 1471-2164-12-172-S4.ZIP › Figure5_18clusters_orf_function_network_p003_GGM_unrestrict_noarraw.jpg]

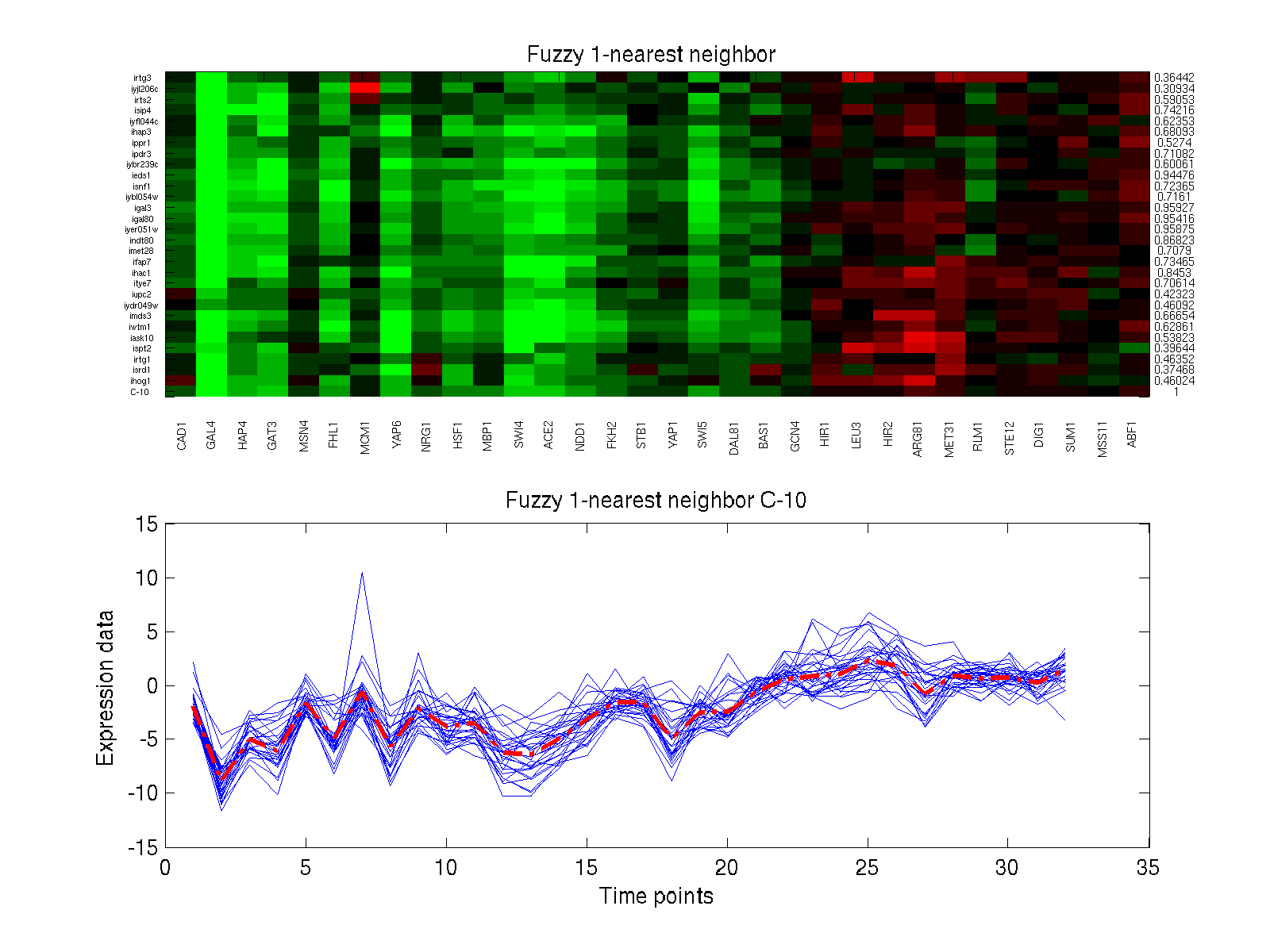

Supplement: Additional file 5 — AddFile5_18clusters_orf_nonfunctional.zip ZIP files. Protein clustering for non-functional binding target. Here contains results (8clusters_orf_unfunction.html) of 18 clusters for non-functional binding sites. [file 1471-2164-12-172-S5.ZIP › C-10.png]

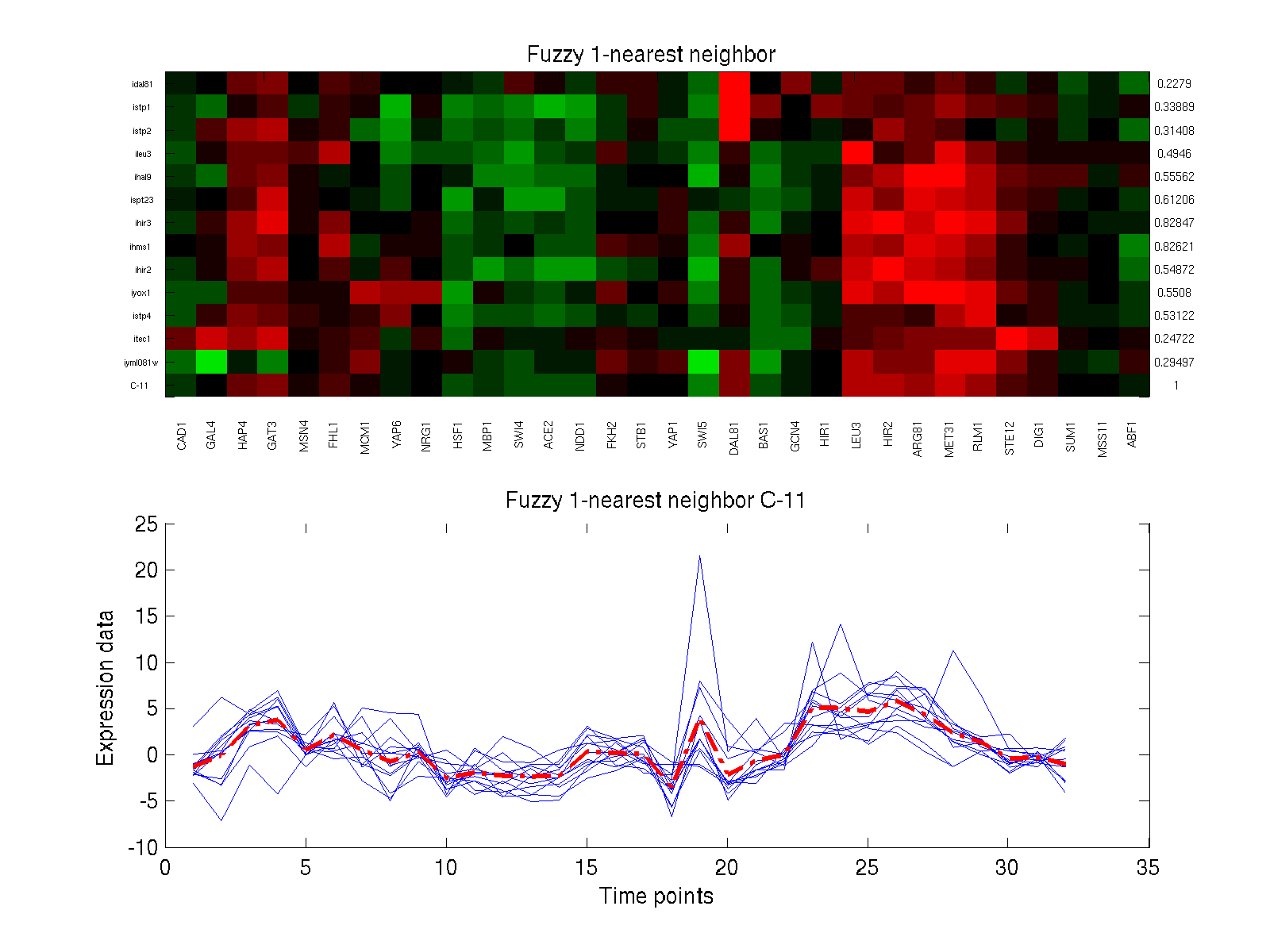

Supplement: Additional file 5 — AddFile5_18clusters_orf_nonfunctional.zip ZIP files. Protein clustering for non-functional binding target. Here contains results (8clusters_orf_unfunction.html) of 18 clusters for non-functional binding sites. [file 1471-2164-12-172-S5.ZIP › C-11.png]

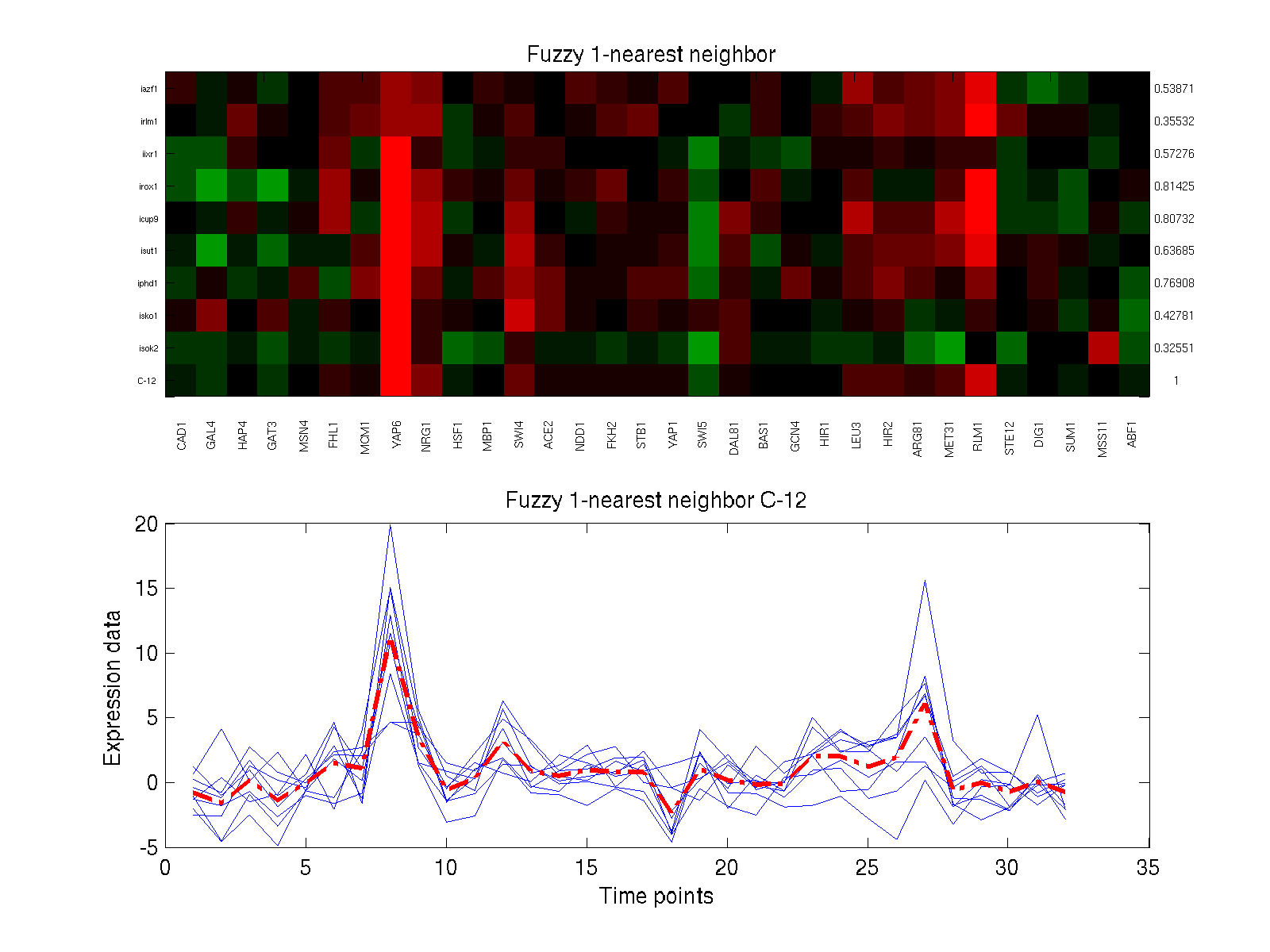

Supplement: Additional file 5 — AddFile5_18clusters_orf_nonfunctional.zip ZIP files. Protein clustering for non-functional binding target. Here contains results (8clusters_orf_unfunction.html) of 18 clusters for non-functional binding sites. [file 1471-2164-12-172-S5.ZIP › C-12.png]

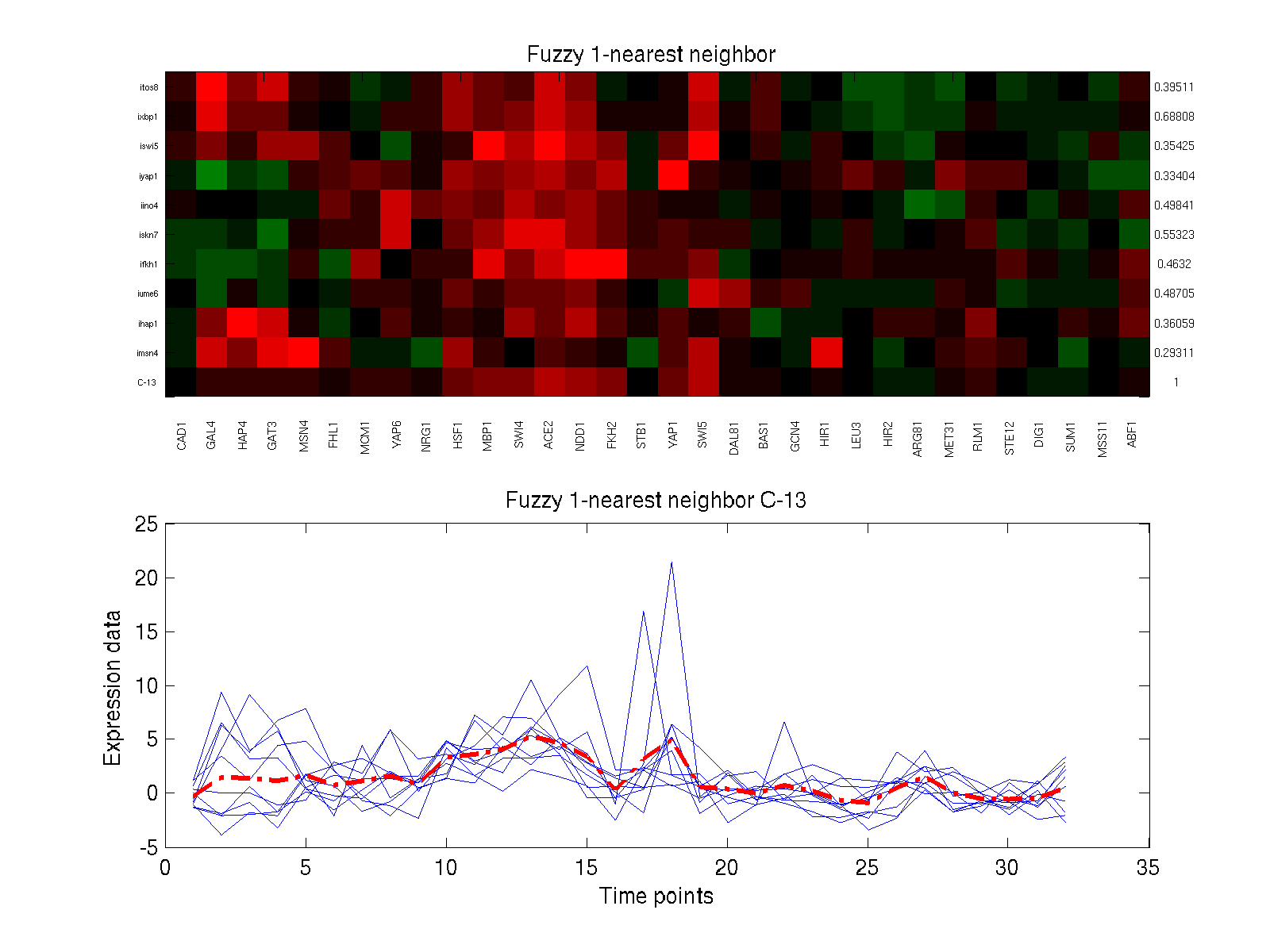

Supplement: Additional file 5 — AddFile5_18clusters_orf_nonfunctional.zip ZIP files. Protein clustering for non-functional binding target. Here contains results (8clusters_orf_unfunction.html) of 18 clusters for non-functional binding sites. [file 1471-2164-12-172-S5.ZIP › C-13.png]

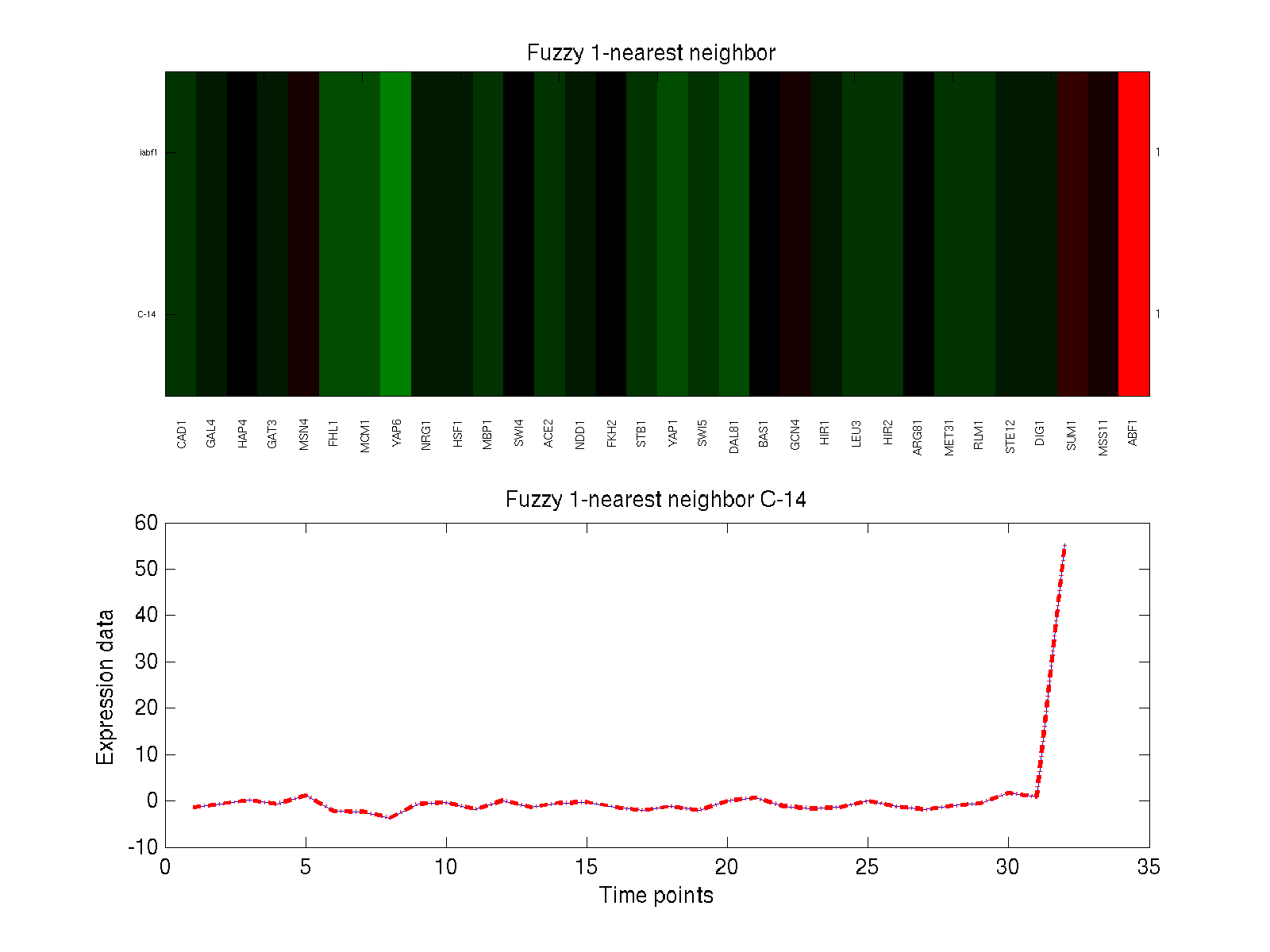

Supplement: Additional file 5 — AddFile5_18clusters_orf_nonfunctional.zip ZIP files. Protein clustering for non-functional binding target. Here contains results (8clusters_orf_unfunction.html) of 18 clusters for non-functional binding sites. [file 1471-2164-12-172-S5.ZIP › C-14.png]

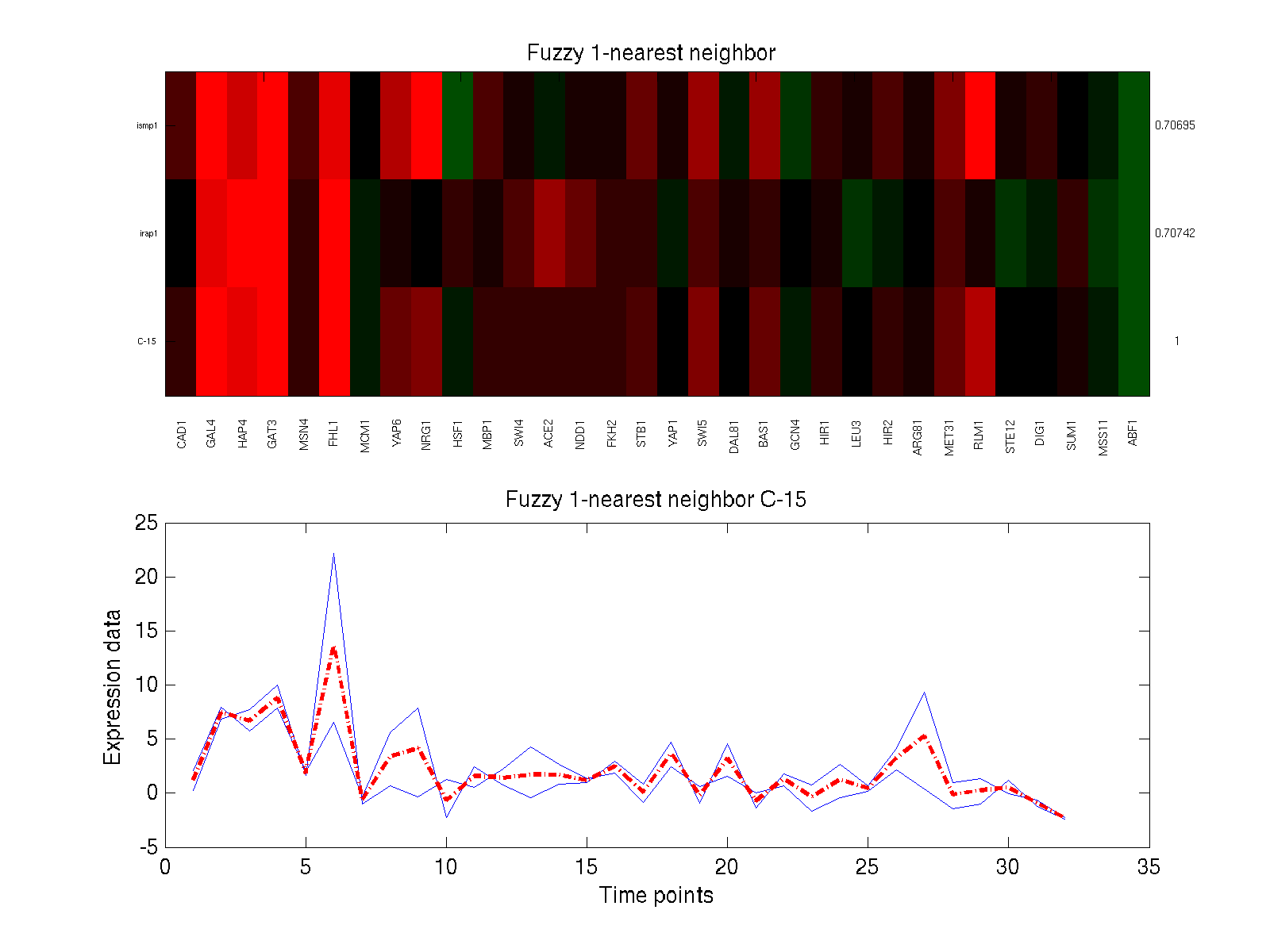

Supplement: Additional file 5 — AddFile5_18clusters_orf_nonfunctional.zip ZIP files. Protein clustering for non-functional binding target. Here contains results (8clusters_orf_unfunction.html) of 18 clusters for non-functional binding sites. [file 1471-2164-12-172-S5.ZIP › C-15.png]

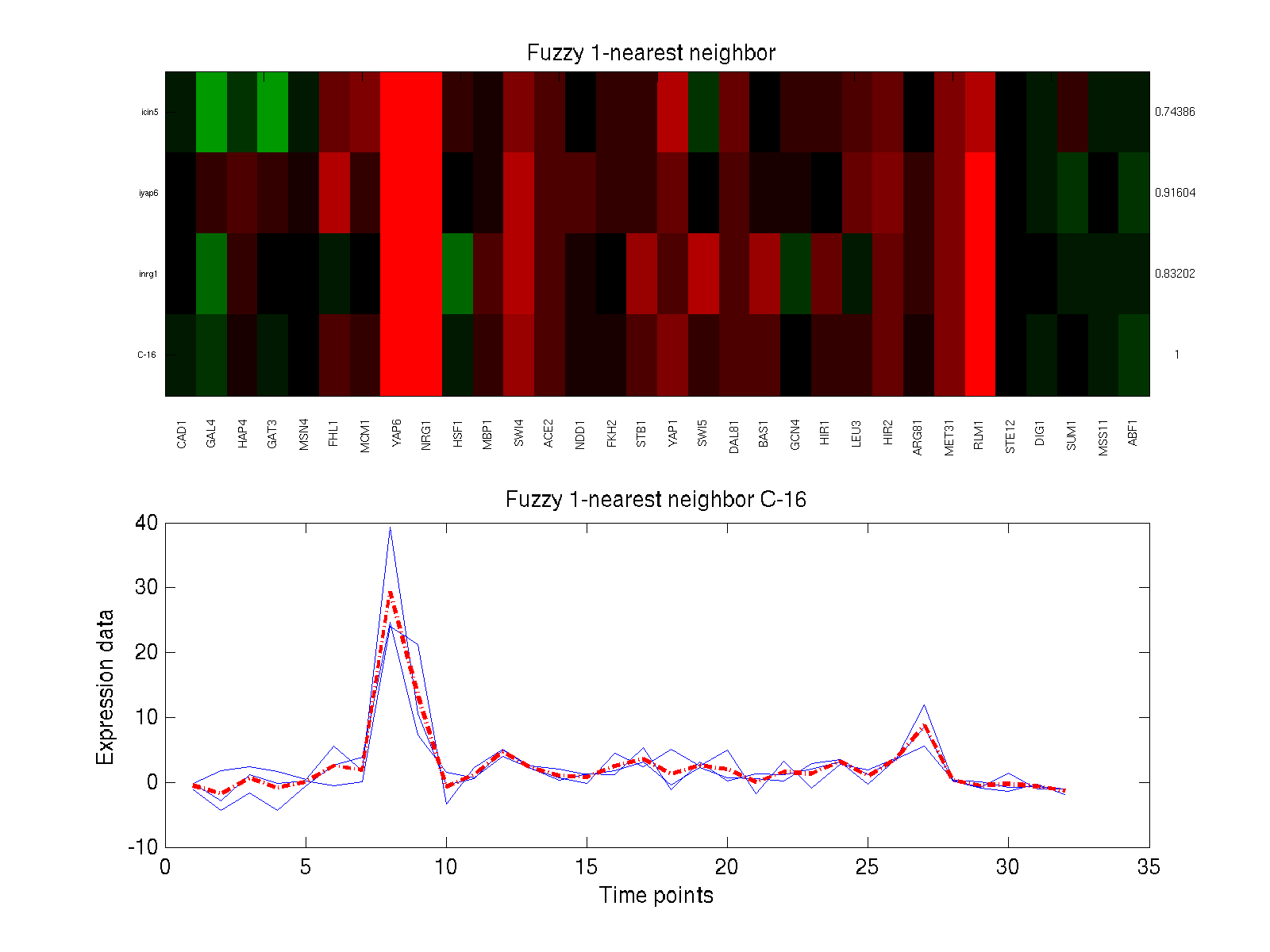

Supplement: Additional file 5 — AddFile5_18clusters_orf_nonfunctional.zip ZIP files. Protein clustering for non-functional binding target. Here contains results (8clusters_orf_unfunction.html) of 18 clusters for non-functional binding sites. [file 1471-2164-12-172-S5.ZIP › C-16.png]

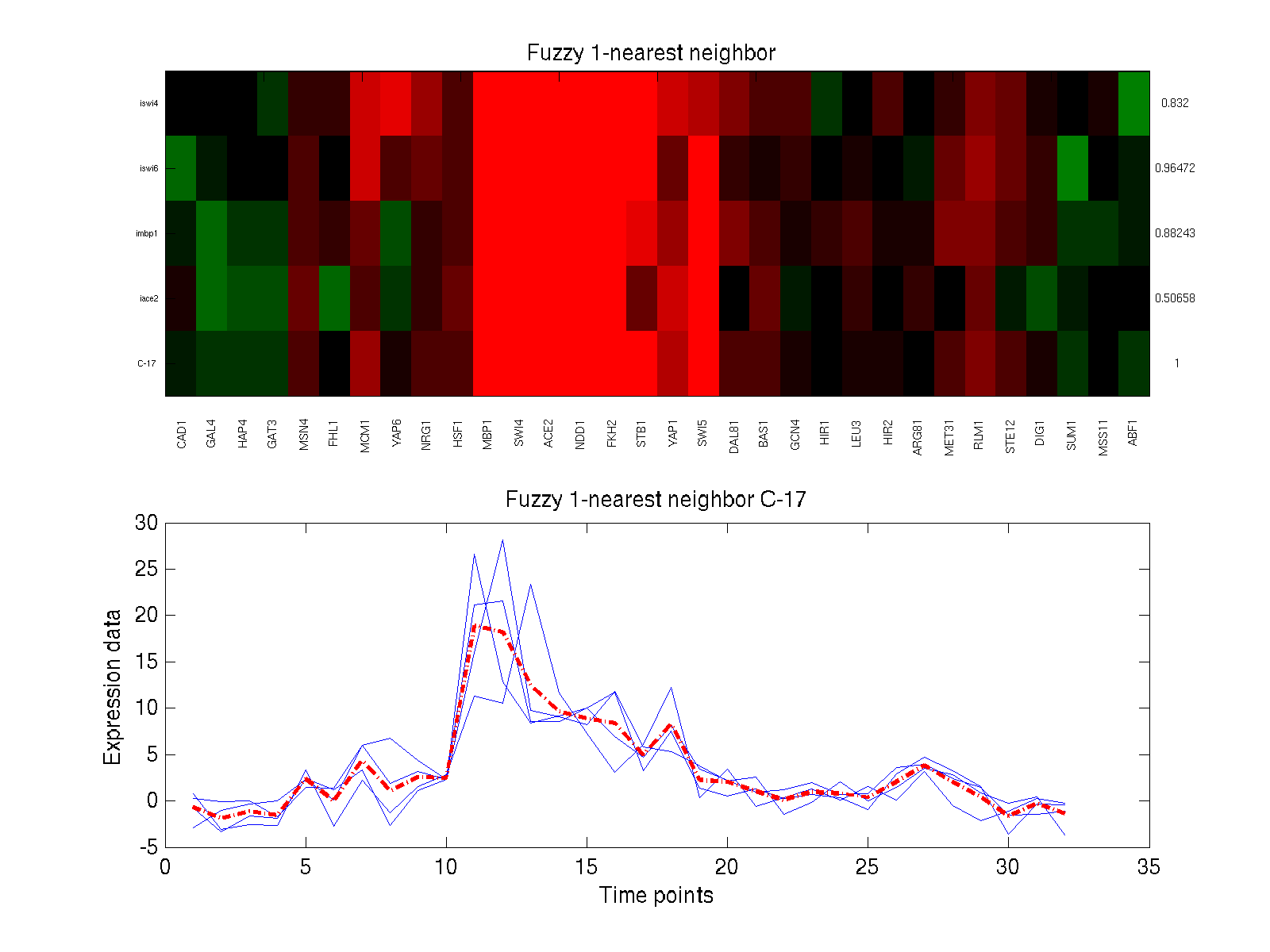

Supplement: Additional file 5 — AddFile5_18clusters_orf_nonfunctional.zip ZIP files. Protein clustering for non-functional binding target. Here contains results (8clusters_orf_unfunction.html) of 18 clusters for non-functional binding sites. [file 1471-2164-12-172-S5.ZIP › C-17.png]

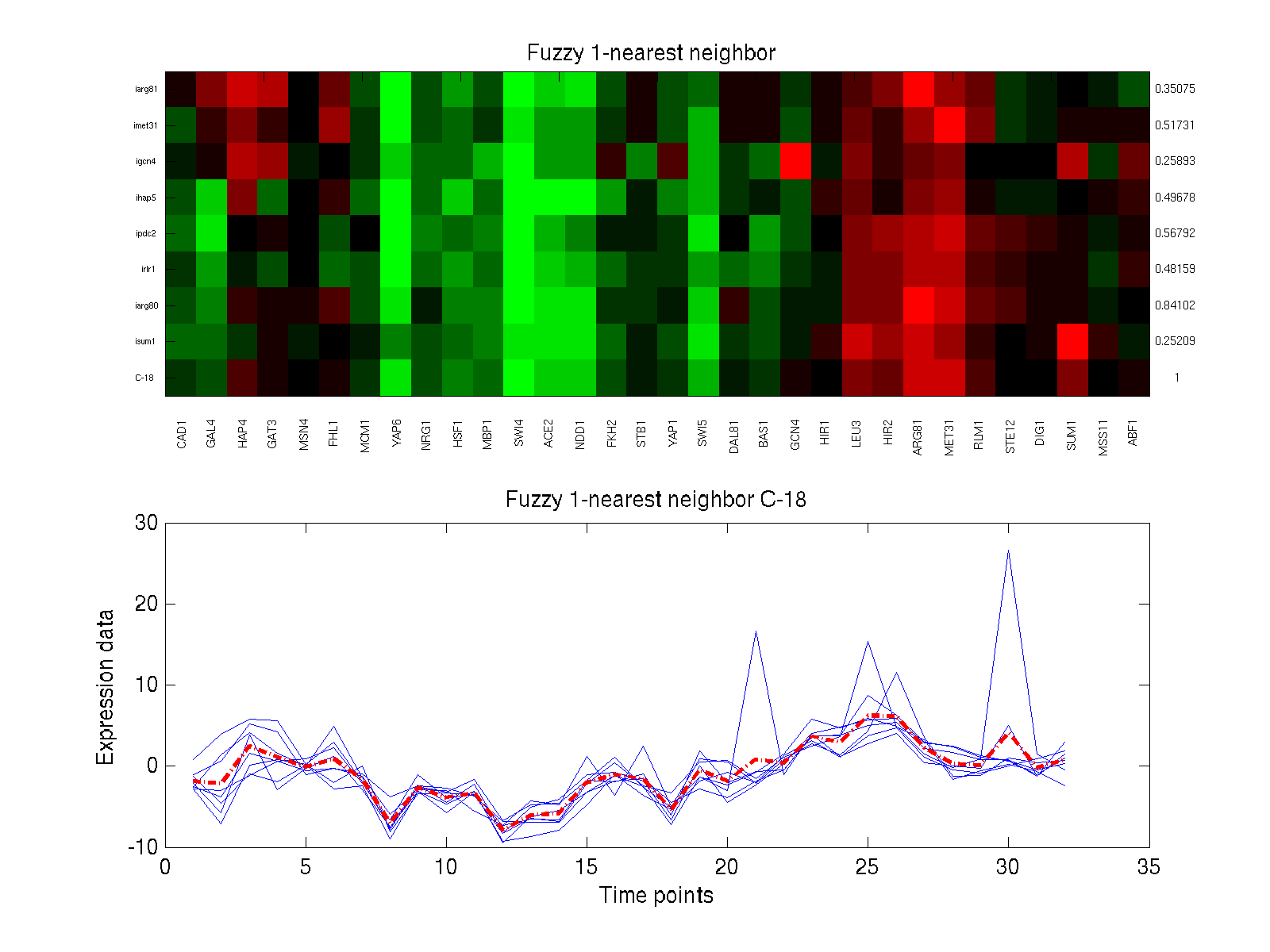

Supplement: Additional file 5 — AddFile5_18clusters_orf_nonfunctional.zip ZIP files. Protein clustering for non-functional binding target. Here contains results (8clusters_orf_unfunction.html) of 18 clusters for non-functional binding sites. [file 1471-2164-12-172-S5.ZIP › C-18.png]

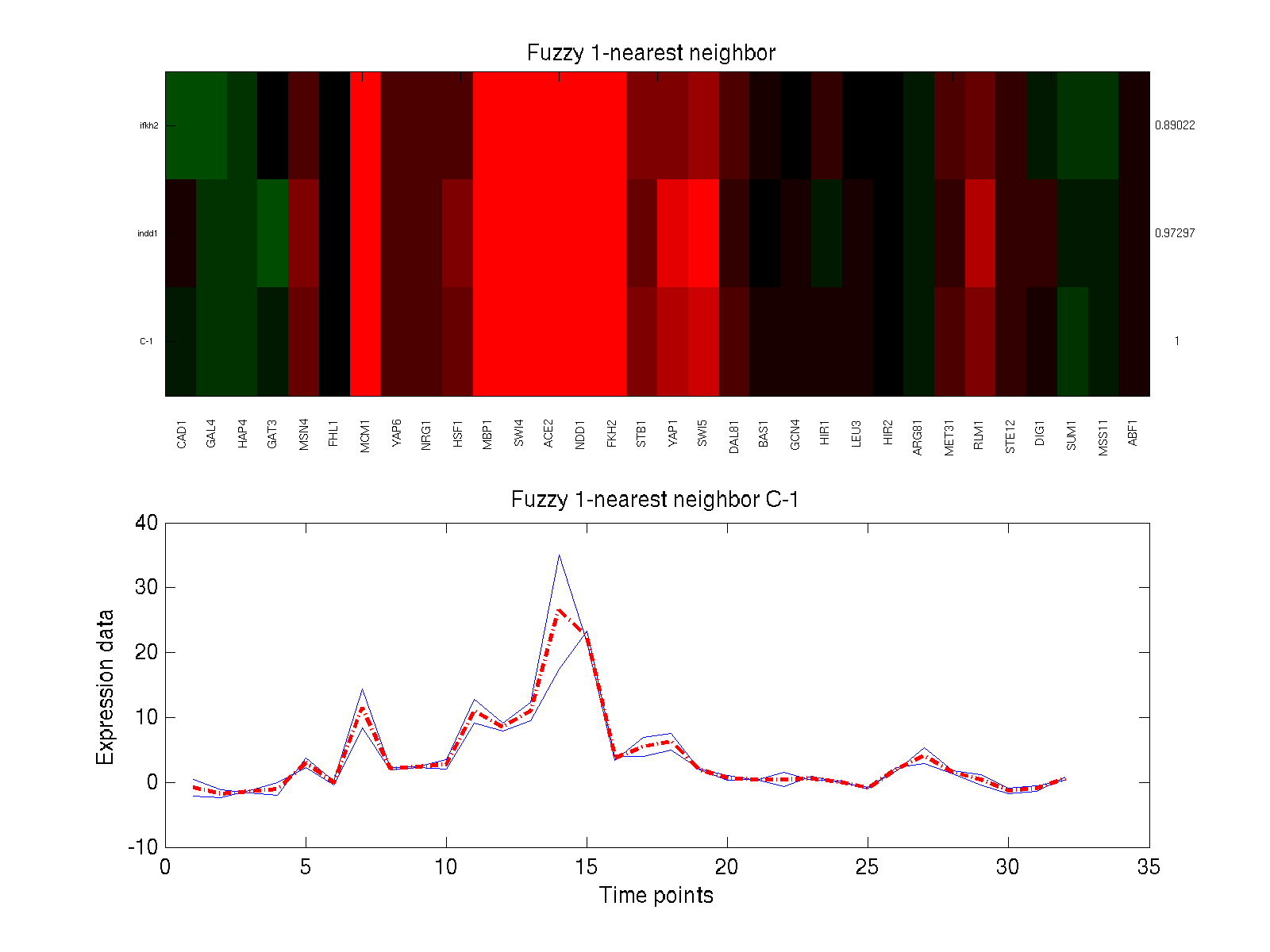

Supplement: Additional file 5 — AddFile5_18clusters_orf_nonfunctional.zip ZIP files. Protein clustering for non-functional binding target. Here contains results (8clusters_orf_unfunction.html) of 18 clusters for non-functional binding sites. [file 1471-2164-12-172-S5.ZIP › C-1.png]

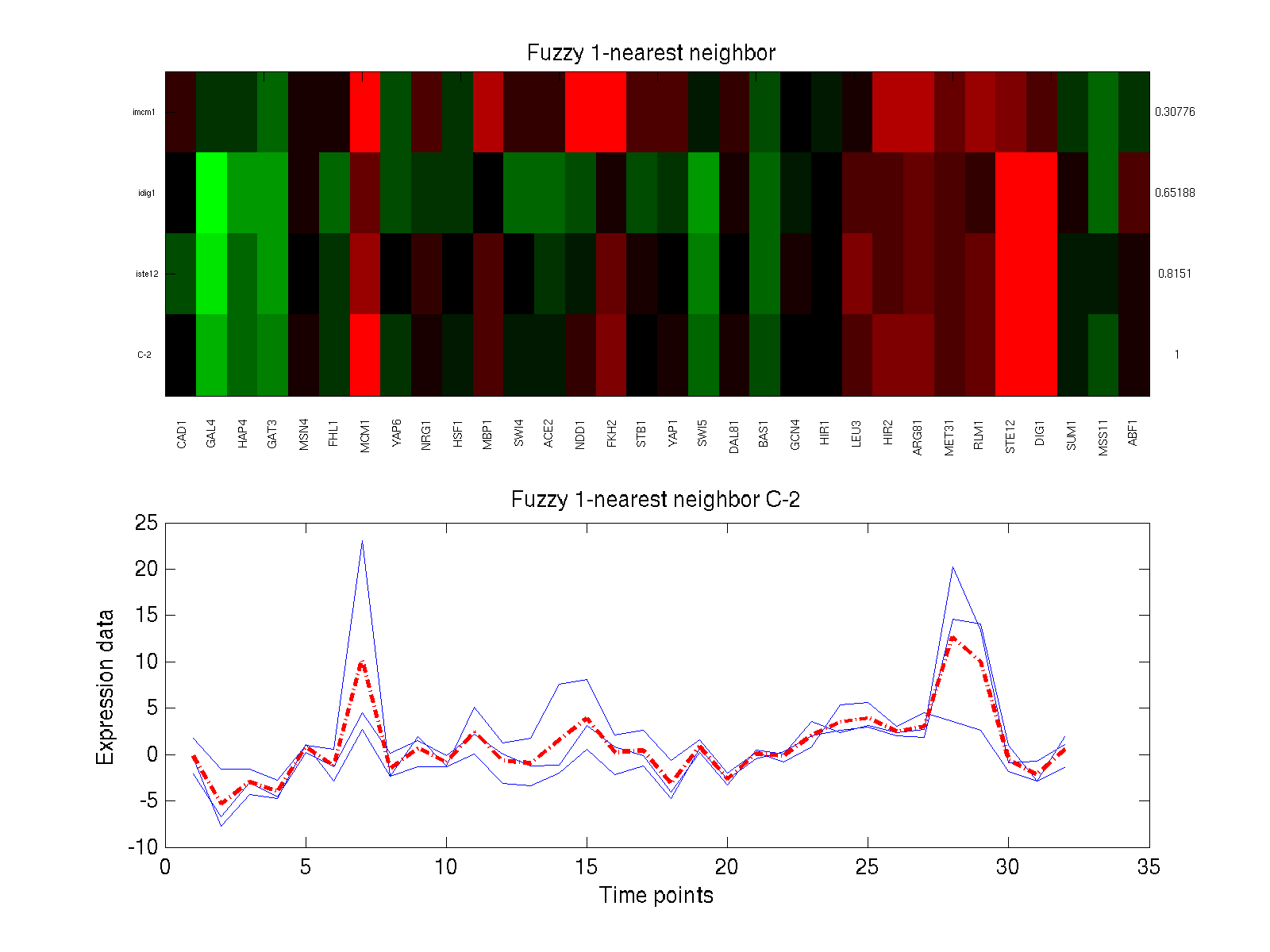

Supplement: Additional file 5 — AddFile5_18clusters_orf_nonfunctional.zip ZIP files. Protein clustering for non-functional binding target. Here contains results (8clusters_orf_unfunction.html) of 18 clusters for non-functional binding sites. [file 1471-2164-12-172-S5.ZIP › C-2.png]

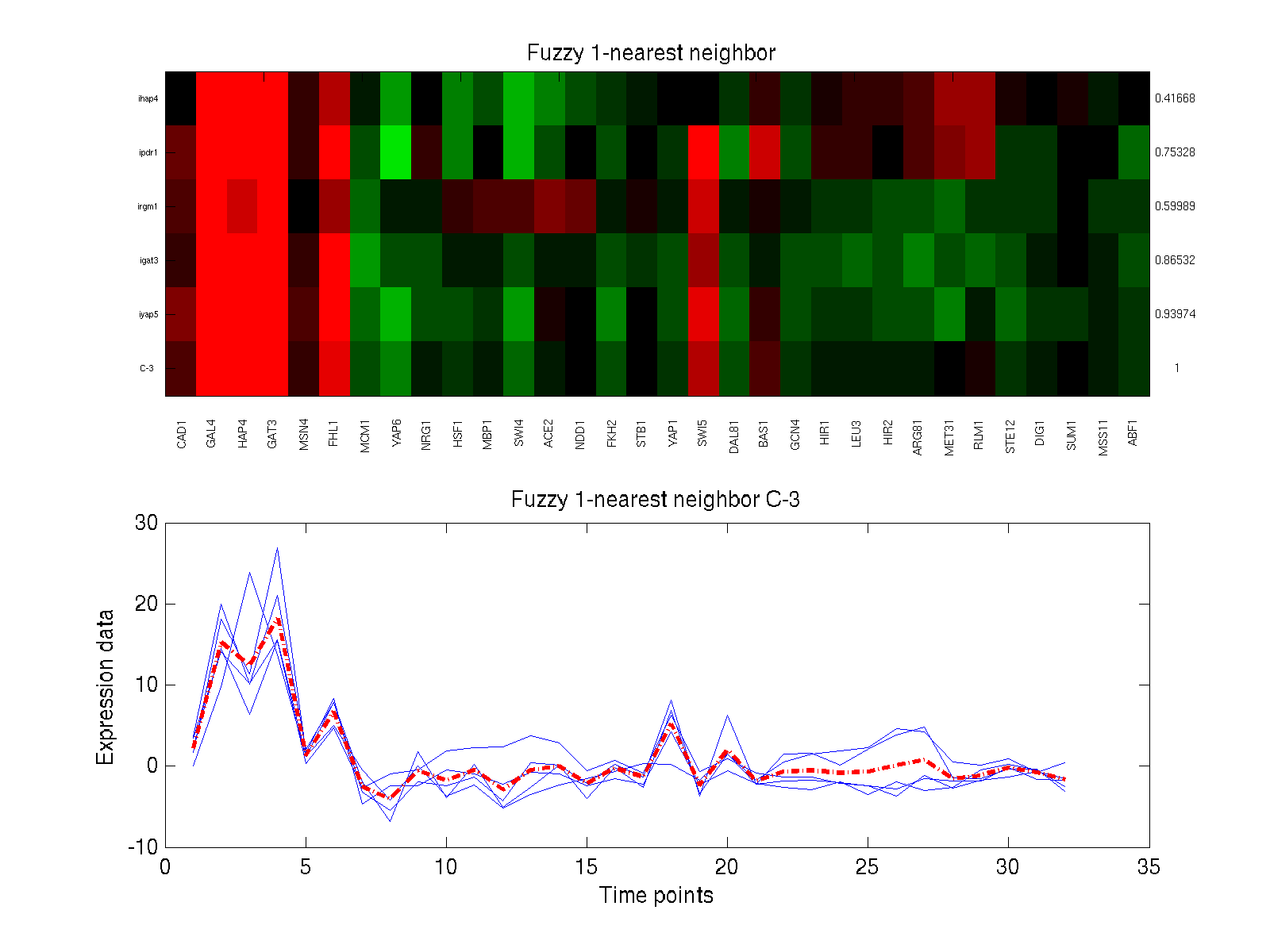

Supplement: Additional file 5 — AddFile5_18clusters_orf_nonfunctional.zip ZIP files. Protein clustering for non-functional binding target. Here contains results (8clusters_orf_unfunction.html) of 18 clusters for non-functional binding sites. [file 1471-2164-12-172-S5.ZIP › C-3.png]

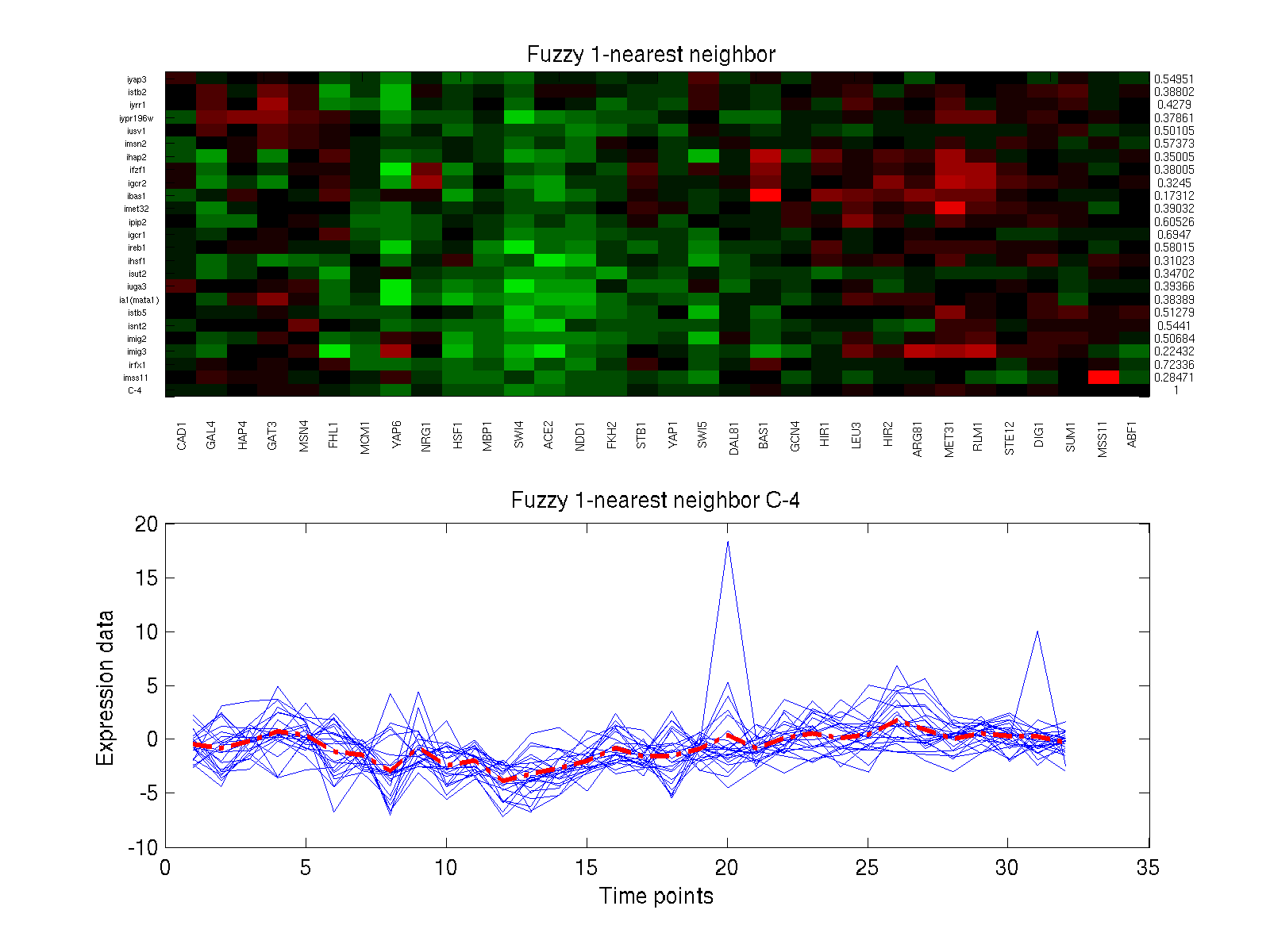

Supplement: Additional file 5 — AddFile5_18clusters_orf_nonfunctional.zip ZIP files. Protein clustering for non-functional binding target. Here contains results (8clusters_orf_unfunction.html) of 18 clusters for non-functional binding sites. [file 1471-2164-12-172-S5.ZIP › C-4.png]

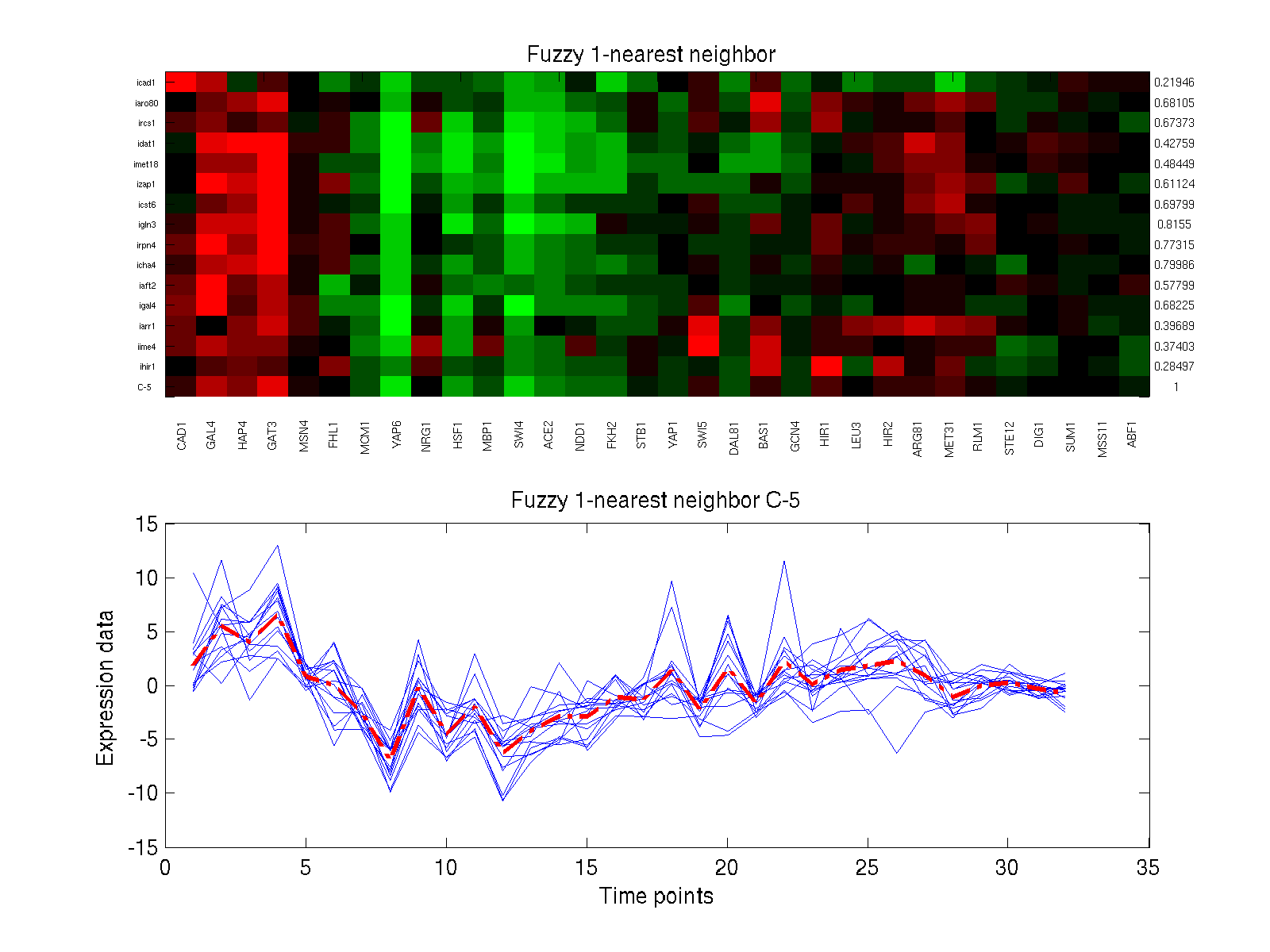

Supplement: Additional file 5 — AddFile5_18clusters_orf_nonfunctional.zip ZIP files. Protein clustering for non-functional binding target. Here contains results (8clusters_orf_unfunction.html) of 18 clusters for non-functional binding sites. [file 1471-2164-12-172-S5.ZIP › C-5.png]

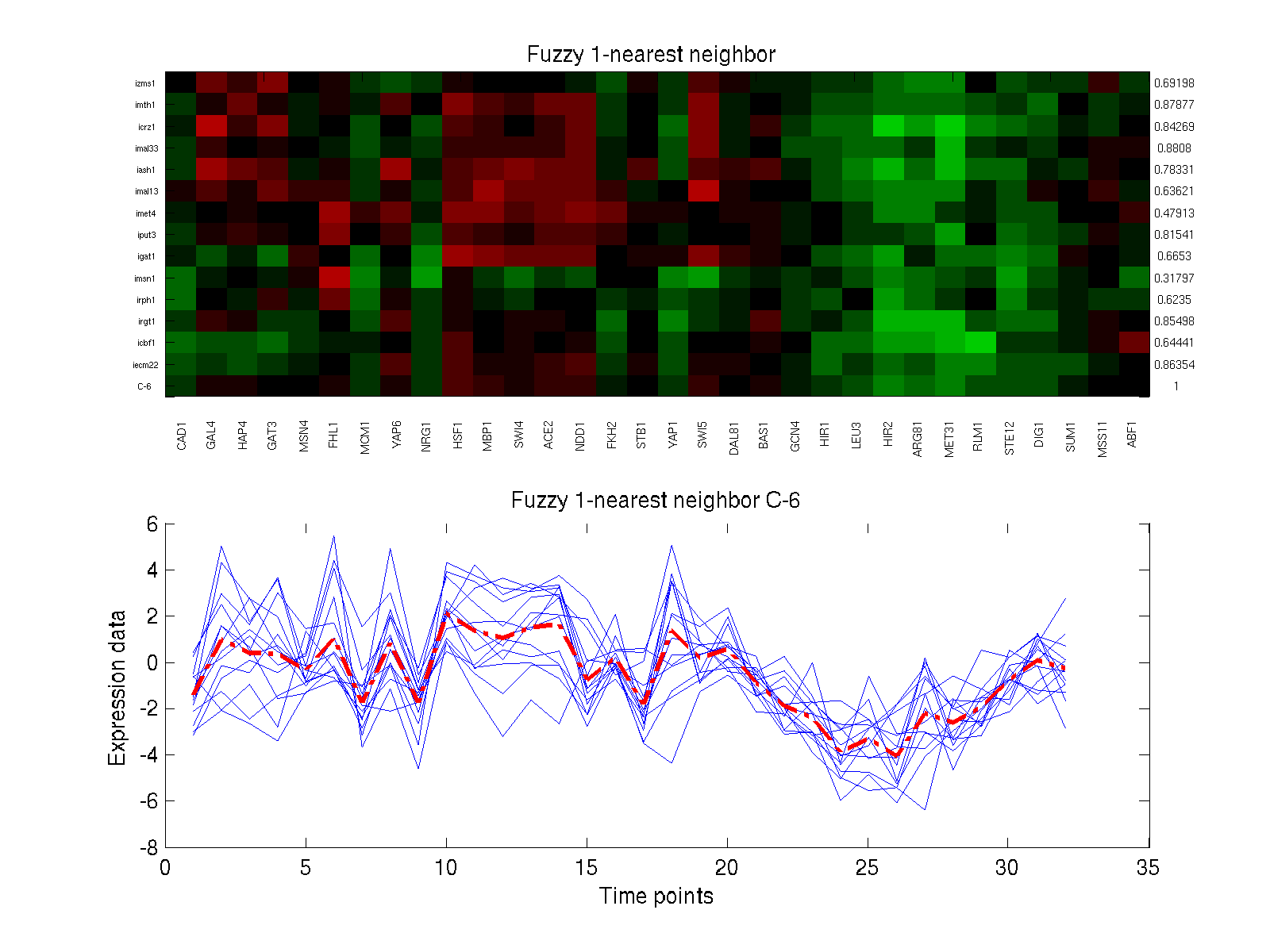

Supplement: Additional file 5 — AddFile5_18clusters_orf_nonfunctional.zip ZIP files. Protein clustering for non-functional binding target. Here contains results (8clusters_orf_unfunction.html) of 18 clusters for non-functional binding sites. [file 1471-2164-12-172-S5.ZIP › C-6.png]

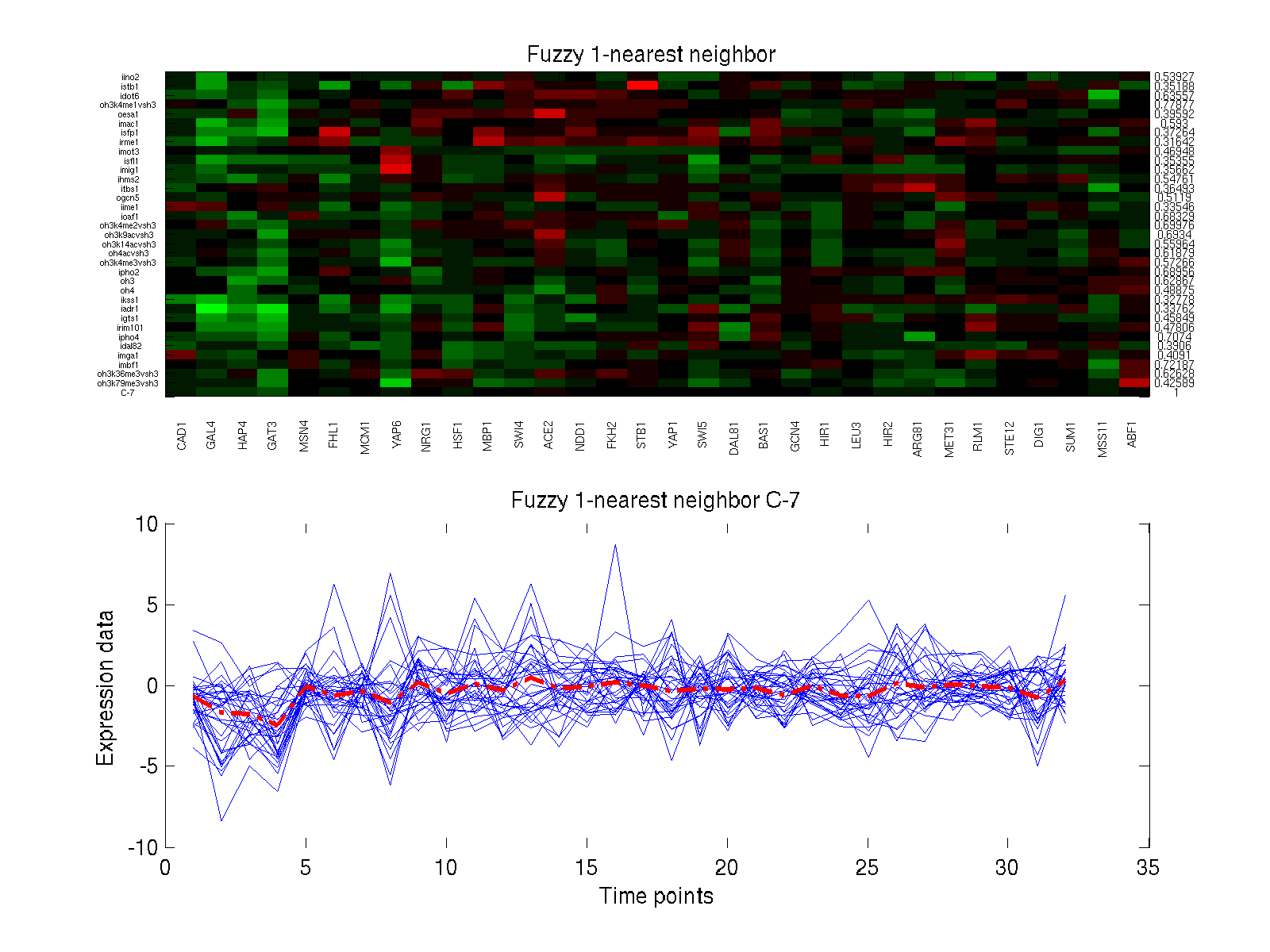

Supplement: Additional file 5 — AddFile5_18clusters_orf_nonfunctional.zip ZIP files. Protein clustering for non-functional binding target. Here contains results (8clusters_orf_unfunction.html) of 18 clusters for non-functional binding sites. [file 1471-2164-12-172-S5.ZIP › C-7.png]

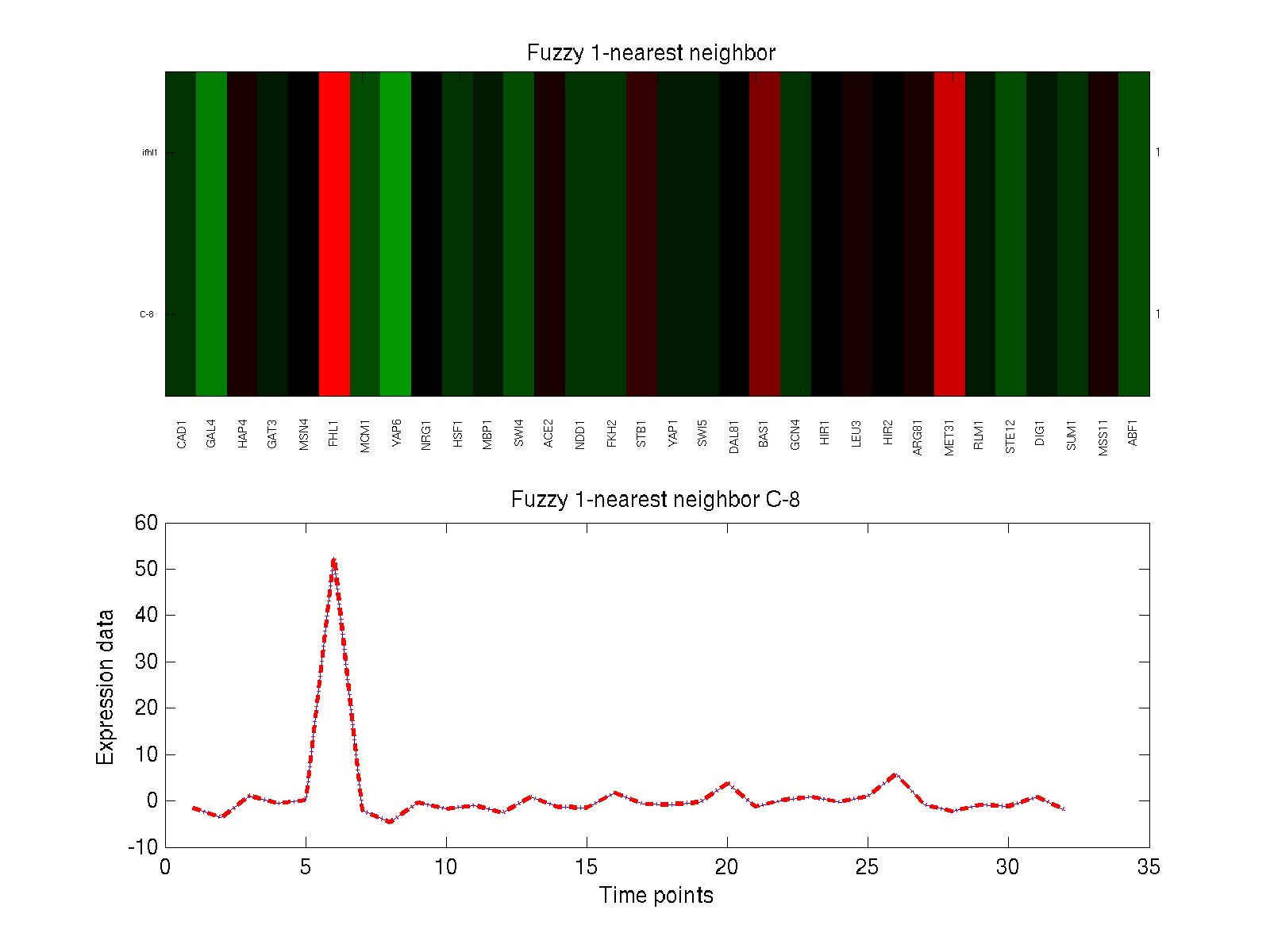

Supplement: Additional file 5 — AddFile5_18clusters_orf_nonfunctional.zip ZIP files. Protein clustering for non-functional binding target. Here contains results (8clusters_orf_unfunction.html) of 18 clusters for non-functional binding sites. [file 1471-2164-12-172-S5.ZIP › C-8.png]

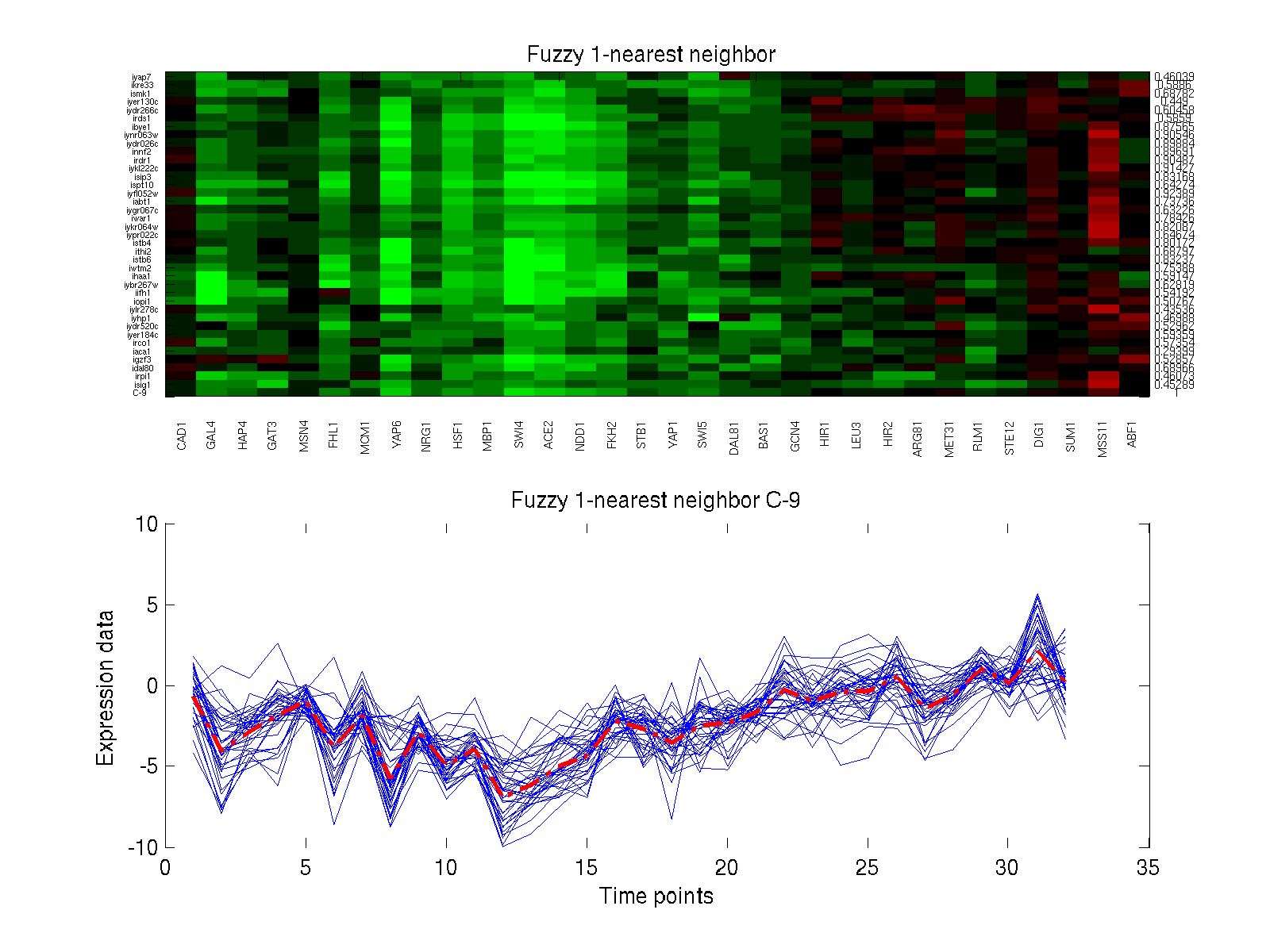

Supplement: Additional file 5 — AddFile5_18clusters_orf_nonfunctional.zip ZIP files. Protein clustering for non-functional binding target. Here contains results (8clusters_orf_unfunction.html) of 18 clusters for non-functional binding sites. [file 1471-2164-12-172-S5.ZIP › C-9.png]

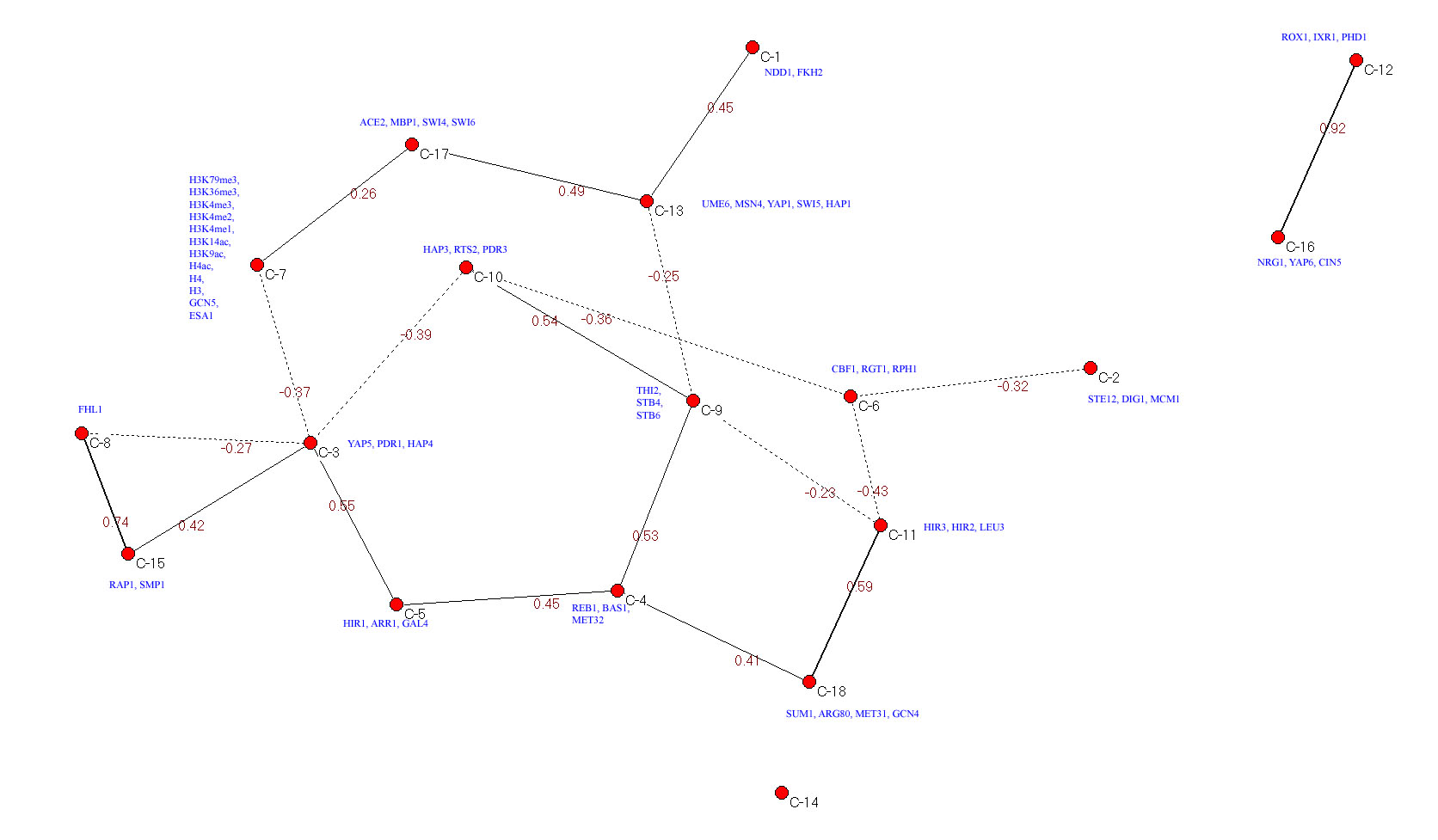

Supplement: Additional file 5 — AddFile5_18clusters_orf_nonfunctional.zip ZIP files. Protein clustering for non-functional binding target. Here contains results (8clusters_orf_unfunction.html) of 18 clusters for non-functional binding sites. [file 1471-2164-12-172-S5.ZIP › Figure6_18clusters_orf_unfunction_network_p003_GGM_unrestrict_noarraw.jpg]
